# Bioactive Naphthoquinone and Phenazine Analogs from the Endophytic *Streptomyces* sp. PH9030 as α-Glucosidase Inhibitors

**DOI:** 10.3390/molecules29153450

**Published:** 2024-07-23

**Authors:** Qingxian Ma, Yani Zhong, Pingzhi Huang, Aijie Li, Ting Jiang, Lin Jiang, Hao Yang, Zhong Wang, Guangling Wu, Xueshuang Huang, Hong Pu, Jianxin Liu

**Affiliations:** 1 China-Pakistan International Science and Technology Innovation Cooperation Base for Ethnic Medicine Development in Hunan Province, Hunan Provincial Key Laboratory for Synthetic Biology of Traditional Chinese Medicine, School of Pharmaceutical Sciences, Hunan University of Medicine, Huaihua 418000, China; 17608470326@163.com (Q.M.); 18874880070@163.com (Y.Z.); 19918037381@163.com (P.H.); 15274565209@163.com (A.L.); 15274519389@163.com (H.Y.); 13773980672@163.com (Z.W.); guangling2024@126.com (G.W.); 2 Jiangxi Drug Inspection Center, Nanchang 330029, China; 15708182636@163.com; 3 Hunan Engineering Technology Research Center for Bioactive Substance Discovery of Chinese Medicine, School of Pharmacy, Hunan University of Chinese Medicine, Changsha 410208, China; jianglin@hnucm.edu.cn

**Keywords:** *Kadsura coccinea*, *Streptomyces*, naphthoquinone, phenazine, α-glucosidase inhibitor

## Abstract

A talented endophytic *Streptomyces* sp. PH9030 is derived from the medicinal plant *Kadsura coccinea* (Lem.) A.C. Smith. The undescribed naphthoquinone naphthgeranine G (**5**) and seven previously identified compounds, **6**–**12**, were obtained from *Streptomyces* sp. PH9030. The structure of **5** was identified by comprehensive examination of its HRESIMS, 1D NMR, 2D NMR and ECD data. The inhibitory activities of all the compounds toward α-glucosidase and their antibacterial properties were investigated. The α-glucosidase inhibitory activities of **5**, **6**, **7** and **9** were reported for the first time, with IC_50_ values ranging from 66.4 ± 6.7 to 185.9 ± 0.2 μM, as compared with acarbose (IC_50_ = 671.5 ± 0.2 μM). The molecular docking and molecular dynamics analysis of **5** with α-glucosidase further indicated that it may have a good binding ability with α-glucosidase. Both **9** and **12** exhibited moderate antibacterial activity against methicillin-resistant *Staphylococcus aureus*, with minimum inhibitory concentration (MIC) values of 16 μg/mL. These results indicate that **5**, together with the naphthoquinone scaffold, has the potential to be further developed as a possible inhibitor of α-glucosidase.

## 1. Introduction

Diabetes mellitus refers to a group of metabolic illnesses that are characterized by abnormally high amounts of glucose in the bloodstream [1]. The global diabetes population is anticipated to exceed 1.3 billion by 2050 [2]. Type 2 diabetes mellitus (T2DM) is the most prevalent type of diabetes and is found on every continent [3]. α-Glucosidase inhibitors are the most effective medications for treating type 2 diabetes mellitus [4]. Currently, the genus *Streptomyces* is the leading developer of novel and advanced secondary metabolites [5]. Acarbose, derived from a strain of the *Streptomyces* genus, was the first α-glucosidase inhibitor to receive approval in Europe and the U.S. for the treatment of type 2 diabetes [6,7,8]. Voglibose is a synthetic derivative of N-substituted valiolamine produced by *Streptomyces hygroscopicus* and was chosen as a possible α-glucosidase inhibitor in 1994 [9]. In 1996, miglitol, a synthetic form of nojirimycin produced by *Streptomyces roseochromogenes*, was discovered to be a possible inhibitor of α-glucosidase [10]. It is believed that *Streptomyces* is a major source of α-glucosidase inhibitors.

**Figure 1:**
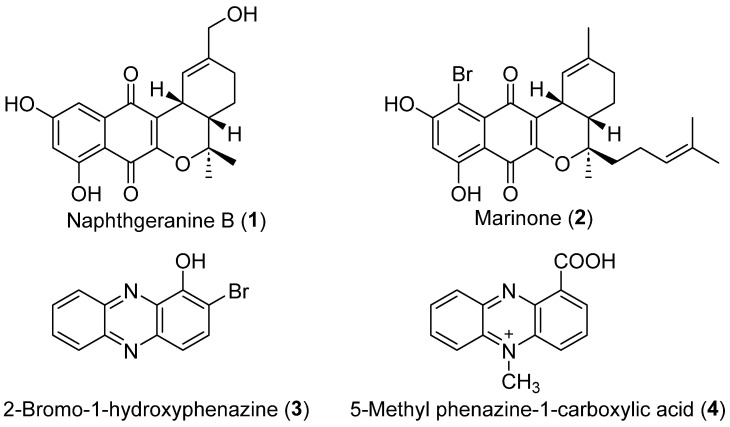
Chemical structures of the naphthoquinone derivatives **1**–**2** and the phenazine derivatives **3**–**4**.

**Figure 2:**
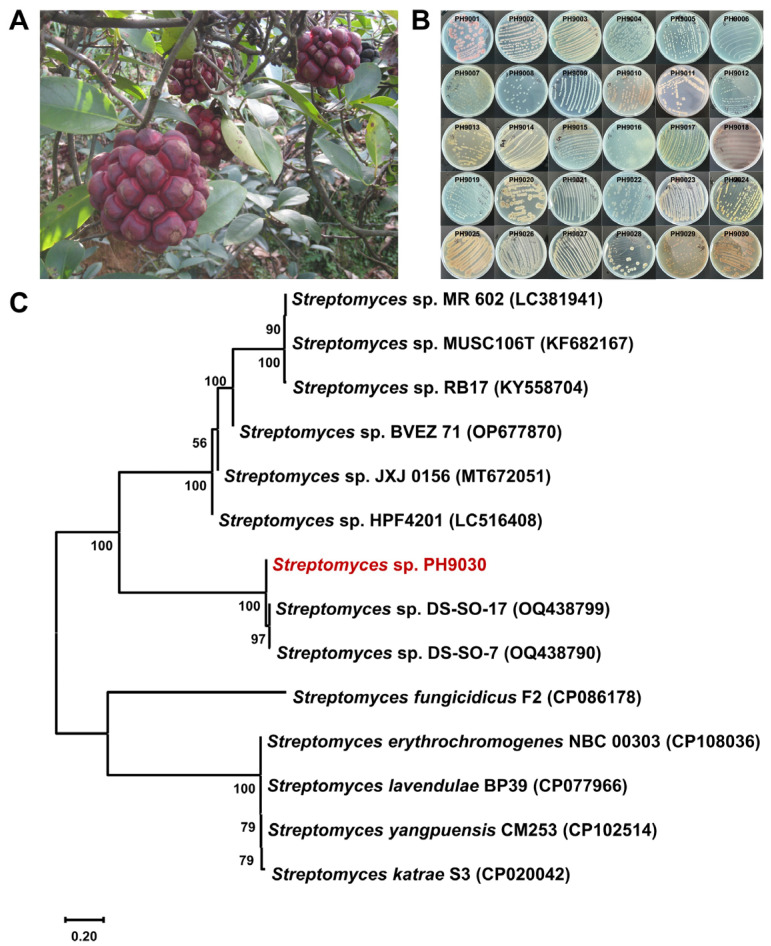
Source of *Streptomyces*. (**A**) The whole plant of *K. coccinea*. (**B**) Morphological characterization of actinobacterial isolates. Colony morphology of different actinobacterial isolates derived from the medicinal plant *K. coccinea*. (**C**) Phylogenetic tree analysis of *S.* sp. PH9030 (red).

**Figure 3:**
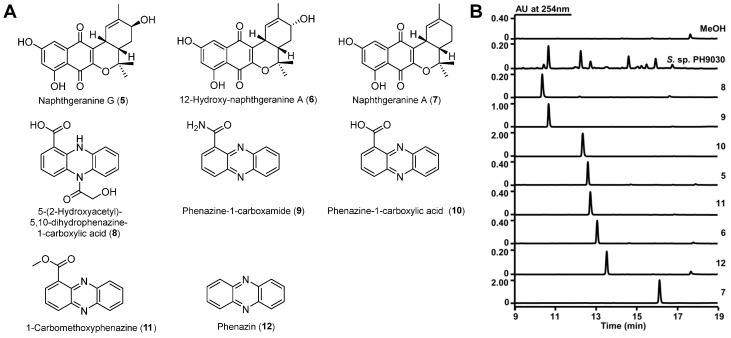
(**A**) Compounds **5**–**12** isolated from *S.* sp. PH9030; (**B**) HPLC analysis of crude extracts and isolated compounds from *S.* sp. PH9030. UV absorption was monitored at 254 nm.

**Figure 4:**
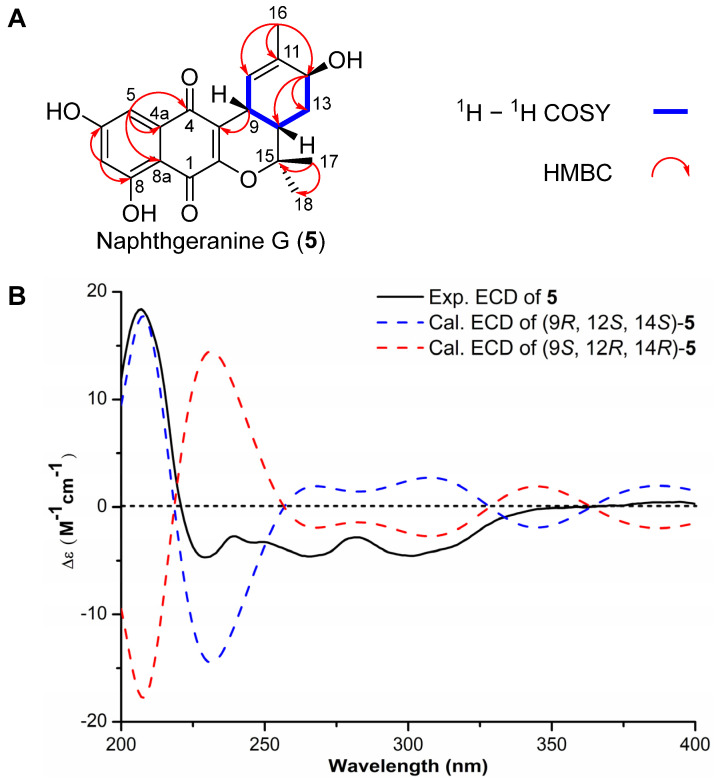
(**A**) Key ^1^H–^1^H COSY and HMBC correlations of compound **5**. (**B**) Experimental and calculated ECD spectra of the compound **5** in MeOH.

Naphthoquinone, an important class of natural products derived from microorganisms, exhibits interesting biological activities [11,12,13,14]. The naphthgeranines, naphterpins, and marinones are meroterpenoid families that share a similar naphthoquinone ring structure and have cyclized C-3 geranyl or farnesyl side chains [15]. For example, naphthgeranine B (Figure 1, **1**), which was isolated in 1990 from soil-derived *Streptomyces* sp. KO-3988, exhibited a potent cytotoxic effect on HeLa S3 cells (IC_50_ = 1.6 μg/mL) [16]. In 1992, marinone (Figure 1, **2**), which is isolated from the marine-derived *Streptomyces* sp. CNB-632, was demonstrated to exhibit inhibitory effects against *Bacillus subrifis*, with MIC of 1 μg/mL [17]. Phenazines are a diverse collection of secondary metabolites with redox activity that are synthesized by several types of bacteria, such as *Streptomyces* and *Pseudomonas*, as well as by *Methanosarcina* species [18]. The fundamental composition of phenazines consists of a pyrazine ring (1,4-diazabenzene) that contains two interconnected benzene rings [19]. Kankanamge et al. discovered two new dimeric phenazine glycosides, tepuazines A and B, and three new monomeric phenazine glycosides, tepuazines C–E, from the metabolites of *Streptomyces virginiae* CMB-CA091 [20]. Several phenazines possess antimicrobial, antifungal, insecticidal and anticancer properties [21,22,23,24,25,26]. 2-Bromo-1-hydroxyphenazine (Figure 1, **3**), a phenazine compound obtained from a marine-derived *Streptomyces* species, has been shown to possess antibacterial properties against *Staphylococcus aureus* ATCC 25923 and *Staphylococcus epidermidis* ATCC 12228 with MIC values of 1.72 µg/mL for both bacteria [27]. 5-Methyl phenazine-1-carboxylic acid (Figure 1, **4**), which is obtained from the bacterium PUW5, showed a specific ability to kill lung (A549) and breast (MDA MB-231) cancer cells with IC_50_ values of 488.7 ± 2.52 nM and 458.6 ± 2.48 nM for lung and breast cancer cells, respectively [28].

**Figure 5:**
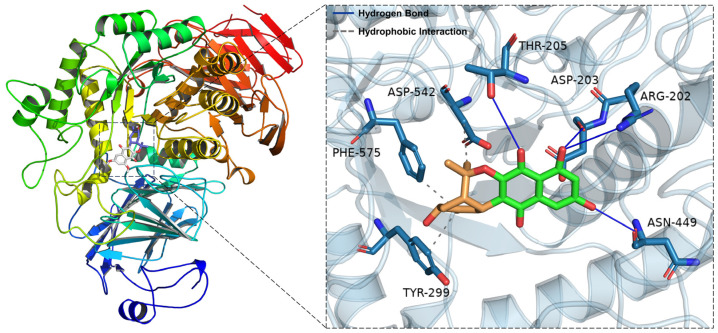
Docking poses and interactions of **5** with α-glucosidase (PDB ID: 2QMJ).

*Kadsura coccinea* (Lem.) A.C. Smith (Figure 2A), “黑老虎” in Chinese, is a perennial climbing shrub of the *Schisandraceae* family, known for its medicinal properties. The roots and stems of this plant are used in traditional Chinese medicine for the treatment of gastroenteric diseases, rheumatism, trauma and pain [29,30]. To the best of our knowledge, only a few reports of endophytes and the natural products that they produce from *K. coccinea*. In our continuous exploration of *Streptomyces* natural products [31,32,33], we have started a project to isolate natural products from endophytic *Streptomyces* strains found in the rhizosphere soil and roots of *K. coccinea*. In this study, we report the isolation of *Streptomyces* from *K. coccinea* (Figure 2B), and that bioactivity-guided natural product isolation has yielded eight compounds including a new naphthoquinone derivative, naphthgeranine G (Figure 3A, **5**), together with two known naphthoquinone derivatives (Figure 3A, **6**–**7**) and five known phenazine derivatives (Figure 3A, **8**–**12**). The ability of these compounds to inhibit α-glucosidase was tested. The data obtained suggest that the majority of these compounds exhibited significant inhibitory effects on α-glucosidase. Among these, **5** exhibited the strongest inhibition, with an IC_50_ value of 66.4 ± 6.7 μM. Molecular docking and molecular dynamics studies were performed to further investigate the interaction, orientation and conformation of **5** over the active site of α-glucosidase. Therefore, **5** is a potential α-glucosidase inhibitor. Furthermore, the evaluation of antibacterial activity revealed that both **9** and **12** had moderate antibacterial activity against methicillin-resistant *Staphylococcus aureus* (MRSA), with MIC values of 16 μg/mL.

## 2. Results and Discussion

### 2.1. Actinomycete Isolation, Small-Scale Fermentation and Antibacterial Activity Assay

Thirty isolates, designated PH9001–PH9030 (Figure 2B), were obtained from the rhizosphere soil and roots of the medicinal plant *K. coccinea*. The plant samples were collected from a mountain ditch in Tongdao County, Huaihua City, Hunan Province, China (Figure 2A). The isolates were obtained through a series of repeated pure cultures on three different agar media (Table S1, MH14, MH15 and MH16). The morphological characteristics of most of the selected isolates are shown in Figure 2B. For further fermentation and antibacterial activity testing, 18 isolates were selected on the basis of their unique characteristics. The most prevalent secondary metabolites of PH9030 in MH18 medium (Table S1) are shown in Figure S1, where they also strongly inhibited MRSA and *Staphylococcus aureus* ATCC 29213 (Table S2, Figures S1 and S2). As a result, the strain PH9030 was chosen for further study.

### 2.2. Identification and Phylogenetic Analysis of Strain PH9030

The partial 16S rRNA gene sequences (Figure S3) were identified, and a phylogenetic tree was constructed via the BLAST tool of the NCBI for the molecular identification of PH9030. These findings indicate that PH9030 is a member of the *Streptomyces* genus. The phylogenetic tree constructed using 16S rRNA sequences (GenBank ID: PP593435) indicates that *Streptomyces* sp. PH9030 exhibits a significant resemblance to *Streptomyces* sp. DS-SO-17 (OQ438799) and *Streptomyces* sp. DS-SO-7 (OQ438790) (Figure 2C). Consequently, larger-scale liquid fermentation of *S.* sp. PH9030 (123 L) was subsequently used to extract natural compounds and assess their effectiveness in inhibiting α-glucosidase and combating bacterial growth.

### 2.3. Structure Elucidation

The crude extract of *S*. sp. PH9030 was fractionated via several techniques including silica gel, MCI gel CHP20, Sephadex LH-20 chromatography and semipreparative HPLC. This process resulted in the isolation of the compounds **5**–**12**, as shown in Figure 3A,B. 12-Hydroxy-naphthgeranine A (**6**), naphthgeranine A (**7**), 5-(2-hydroxyacetyl)-5,10-dihydrophenazine-1-carboxylic acid (**8**), phenazine-1-carboxamide (**9**), phenazine-1-carboxylic acid (**10**), 1-carbomethoxyphenazine (**11**) and phenazin (**12**) are known compounds, and their structures were established on the basis of comparisons of their 1D and 2D NMR spectra, HRESIMS data and UV spectra with the literature [34,35,36,37,38] (Figure 3A and Figures S15–S62). Phenazine-1-carboxylic acid (PCA, **10**) is a physiologically active chemical that has the potential to prevent and control crop diseases. In 2011, the Ministry of Agriculture of China recognized “Shenzimycin” as a pesticide [23,39].

Naphthgeranine G (**5**) was isolated as a yellow powder. Its molecular formula was established as C_20_H_20_O_6_ on the basis of (−)-HRESIMS analysis (Figure S12) at *m*/*z* 355.1177 [M − H]^−^ (calcd for C_20_H_19_O_6_, 355.1187), suggesting eleven degrees of unsaturation. The ^13^C NMR spectrum of **5** (Table 1), DEPT-135 and DEPT-90 revealed a total of twenty signals containing two ester carbonyls (*δ*_C_ 184.2, 178.5), three phenolic carbons (*δ*_C_ 164.9, 164.8, 65.3), six nonprotonated carbons (*δ*_C_ 154.3, 136.5, 134.0, 123.0, 107.6, 79.8), three olefinic methine carbons (*δ*_C_ 136.5, 120.1, 104.3), two methine carbons (*δ*_C_ 33.9, 30.8), one methylene carbon (*δ*_C_ 29.4) and three methyl carbons (*δ*_C_ 25.5, 24.7, 21.2) (Figures S4). The ^1^H NMR spectrum (Table 1) also indicated the presence of three sp^2^ methines [*δ*_H_ 6.64 (1H, s, H-5), 6.10 (1H, d, *J* = 4.8 Hz, H-7) and 6.03 (1H, s, H-10)], three methines [*δ*_H_ 3.75 (1H, m, H-12), 3.42 (1H, m, H-9) and 2.04 (1H, ddd, *J* = 13.2, 6.2, 2.9 Hz, H-14)], one methylene [*δ*_H_ 1.83 (1H, dd, *J* = 13.6, 2.7 Hz, H-13) and 1.23 (1H, m, H-13)] and three methyl groups [*δ*_H_ 1.69 (3H, s, H-16), 1.42 (3H, s, H-17) and 1.26 (3H, s, H-18)]. The ^1^H and ^13^C NMR data of **5** are similar to those of 12-hydroxy-naphthgeranine A previously isolated from *Streptomyces* sp. XZYN-4 [34]. This was confirmed by the HMBC correlations from H-9 to C-2 (*δ*_C_ 154.3), C-3 (*δ*_C_ 123.0), C-10 (*δ*_C_ 120.1), C-11 (*δ*_C_ 136.5), C-13 (*δ*_C_ 29.4) and C-14 (*δ*_C_ 33.9), H-13 to C-9 (*δ*_C_ 30.8), C-11 (*δ*_C_ 136.5), C-12 (*δ*_C_ 65.3) and C-15 (*δ*_C_ 79.8), H-14 to C-15 (*δ*_C_ 79.8), C-12 (*δ*_C_ 65.3), C-9 (*δ*_C_ 30.8) and C-13 (*δ*_C_ 29.4), H-16 to C-10 (*δ*_C_ 120.1), C-11 (*δ*_C_ 136.5) and C-12 (*δ*_C_ 65.3), H-17 to C-14 (*δ*_C_ 33.9), C-15 (*δ*_C_ 79.8) and C-18 (*δ*_C_ 24.7) and H-18 to C-14 (*δ*_C_ 33.9), C-15 (*δ*_C_ 79.8) and C-17 (*δ*_C_ 25.5). The sequence from H-10 to H-12 through H-9, H-14 and H-13 was revealed using the COSY spectrum (Figure 4A). Electronic circular dichroism (ECD) calculations were subsequently employed to determine the absolute configuration of **5** by comparing the ECD spectra of (9*R*, 12*S*, 14*S*)-**5** and (9*S*, 12*R*, 14*R*)-**5** with the experimental results, which suggested a (9*R*, 12*S*, 14*S*)-**5** configuration (Figure 4B). Accordingly, the structure of **5** was elucidated as depicted in Figure 4.

### 2.4. In Vitro α-Glucosidase Inhibitory Activity

The in vitro α-glucosidase inhibitory activities of the compounds **5**–**12** were assessed. The α-glucosidase inhibitory activities of **5**, **6**, **7** and **9** were reported for the first time, with IC_50_ values ranging from 66.4 ± 6.7 to 185.9 ± 0.2 μM. Acarbose was used as a positive control, and the results are summarized in Table 2. These findings demonstrated that the majority of the compounds had a very promising α-glucosidase inhibitory activity. Notably, **5** exhibited the highest potency (IC_50_ = 66.4 ± 6.7 μM), surpassing the activity of acarbose (IC_50_ = 671.5 ± 0.2 μM). The investigation of the structure‒activity connection revealed that the presence of C12-OH greatly enhances the molecular framework of α-glucosidase inhibitory activity. Furthermore, the 12*S*-conformation exhibits greater strength than the 12*R*-conformation.

### 2.5. Molecular Docking Simulations of ***5*** with α-Glucosidase

The software AutoDock Vina 1.1.2 was used for molecular docking research to investigate the interactions between **5**–**12** and α-glucosidase. These compounds, with binding energies greater than that of acarbose, had blocking effects, whereas the compounds with binding energies lower than acarbose had no activity. These findings matched the results of the experiments (Table 2 and Table S6). Owing to its outstanding α-glucosidase inhibitory action, **5** was our primary focus. Figure 5 shows the molecular docking models of **5**. The docking results revealed that **5** formed four hydrogen bonds with Asp-203, Arg-202, Thr-205 and Asn-449 and three hydrophobic interactions with Asp-542, Phe-575 and Tyr-299 (Figure 5). Additionally, to compare the various interactions, we performed molecular docking of acarbose (Figure S63). The affinities of the mentioned inhibitors were calculated, and the results revealed that acarbose has a binding energy of 6.7 kcal/mol and that **5** has a binding energy of 7.2 kcal/mol (Table 3). The docking findings suggested that, compared with acarbose, **5** had a stronger influence on the binding contacts with the active pocket of α-glucosidase, impacting its inhibitory activity.

### 2.6. Molecular Dynamics Simulations

**Figure 6:**
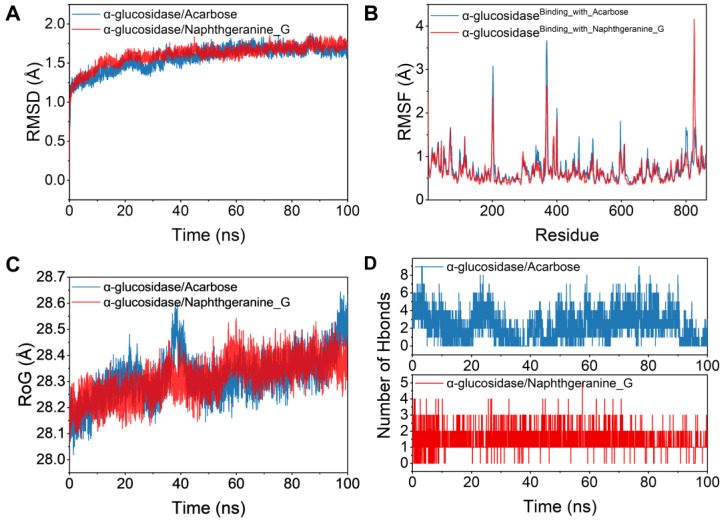
Molecular dynamics of acarbose and **5** with residues in the active pocket of α-glucosidase. (**A**) The RMSD values of the complex and protein backbone systems were calculated throughout the dynamic simulation. (**B**) RMSF changes the shapes of the residues that bond in both free proteins and complicated systems. (**C**) The gyration radius of the four systems was measured during the molecular dynamics simulation. (**D**) The quantity of hydrogen bonds present in the molecular dynamics simulation.

A molecular dynamics simulation was subsequently conducted under physiologically simulated circumstances to elucidate the binding pattern, stability and molecular interaction mode of **5** with the α-glucosidase protein complex. Structural stability is often assessed on the basis of the presence of low root-mean-square deviation (RMSD) and root-mean-square fluctuation (RMSF) values [40]. The RMSD fluctuation graphs throughout the simulation display the RMSD of the two systems, α-glucosidase/acarbose and α-glucosidase/naphthgeranine G (**5**), as shown in Figure 6A. In the first five ns of the simulation, the two systems converge gradually. In the subsequent simulations, the systems maintain relatively stable fluctuations, with the RMSD keeping the fluctuations between 1 and 2 Å. On the basis of their steady fluctuations, the two systems are stable together. As shown in Figure 6B, all proteins had minimal RMSF values after binding tiny ligands, indicating a solid core structure. Consequently, these proteins are more rigid when bound to small molecules, resulting in the inhibitory action of these small molecules. Significantly, there is a substantial overlap between the red line and the blue line, suggesting that the two tiny chemicals have comparable impacts on the proteins. The radius of gyration (RoG) is a measure of the compactness of a system and may indicate the degree of densification. Figure 6C clearly shows that the α-glucosidase/acarbose and α-glucosidase/naphthgeranine G (**5**) systems exhibit similar binding effects and vacillate accordingly. A thorough investigation revealed that the RoG of α-glucosidase/naphthgeranine G (**5**) mostly decreased during the simulation. This finding indicates that the system became more condensed, suggesting a higher level of binding affinity.

To better represent the binding modalities of small molecules and target proteins, we computed the binding energies via the MM-GBSA approach, which is based on the trajectories of the molecular dynamic simulations. According to Table S5, the binding energy of the α-glucosidase/acarbose complex was −11.8 ± 4.0 kcal/mol, and that of the α-glucosidase/naphthgeranine G (**5**) complex was −16.6 ± 1.4 kcal/mol. Smaller values suggest stronger binding, and negative values suggest that the two molecules may bind to the target proteins. Our calculations indicate that α-glucosidase/naphthgeranine G (**5**) binds more effectively and has a marginally lower value than acarbose. One of the stronger noncovalent ways to bind is through hydrogen bonds, and having more hydrogen bonds results in better binding. Figure 6D shows that the number of hydrogen bonds between α-glucosidase and acarbose remained between one and nine and mostly changed between three and four. These findings suggest that hydrogen bonds are important for keeping the binding of acarbose stable. Hydrogen bonding is among the strongest noncovalent binding interactions, and a greater number of hydrogen bonds indicates better binding. The number of hydrogen bonds in the α-glucosidase/naphthgeranine G (**5**) complex changed considerably over the simulation period (0–5) but mostly remained at 1–2. These findings suggest that hydrogen bonding plays a minor role in the interaction between α-glucosidase and naphthgeranine G (**5**). In summary, naphthgeranine G (**5**) binds to α-glucosidase more effectively than acarbose. This is in line with the observed experimental findings.

### 2.7. Antibacterial Activities of ***5***–***12***

The MIC values of **5**–**12** against *Staphylococcus aureus* ATCC 29213, MRSA, *Klebsiella pneumoniae* ATCC 13883 and *Pseudomonas aeruginosa* ATCC 9027 were determined via a broth dilution assay in 96-well plates, with levofloxacin as a control (Table S7). Both **9** and **12** demonstrated modest levels of antibacterial activity against MRSA, with MIC values of 16 μg/mL (Figure S64).

## 3. Materials and Methods

### 3.1. Sample Collection

The medicinal plant *K. coccinea* was collected from a mountain ditch in Tongdao County, Huaihua City, Hunan Province, China (E109°25′53″, N25°52′00″). The plant was identified by the Department of Chinese Pharmacy of the School of Pharmaceutical Sciences, Hunan University of Medicine.

### 3.2. Isolation of Endophytes

The separation methods used for endophytic actinomycetes are detailed in the Supplementary Materials.

### 3.3. Genomic DNA Extraction, 16S rRNA Gene Sequencing and Phylogenetic Tree Construction

*S.* sp. PH9030 was selected for cultivation in 50 mL of TSB medium. The mixture was agitated at 220 rpm for two days at 30 °C. The resulting mycelium biomass was then obtained by separating it via centrifugal precipitation. In accordance with the instructions provided by the manufacturer, the mycelium biomass that was collected was used in the process of extracting genomic DNA via the Ezup Column Bacteria Genomic DNA Purification Kit. Genomic DNA was extracted via conventional procedures [41]. The verified DNA was preserved at a temperature of −20 °C for future use. The 16S rRNA gene was amplified via the universal primer pair 27F/1492R under these conditions [42]. The resulting PCR products were subsequently cloned and sequenced [42]. The NCBI-BLAST database was used to perform sequence similarity searches and ascertain pairwise similarity values. The GenBank database has been updated with the partial sequences of the 16S rRNA gene that were obtained from *S.* sp. PH9030. Additionally, the accession code PP593435 was allocated to this sequence. A phylogenetic tree was created via the neighbor joining technique via MEGA 11.0 software. The 13 strains that were closest to each other at the genus level were chosen on the basis of the 16S rRNA sequence, which was compared with the database [43]. Bootstrap values (expressed as percentages of 1000 replications) over 50% are shown at branching nodes. The bar was 0.20 substitutions per nucleotide position.

### 3.4. General Methods

The equipment, including those used for optical rotation, HRESIMS, NMR and ECD, as well as the usual reagents used for chemical separation and biological assessment, were identical to those previously reported [32]. The details are listed in the Supplementary Materials.

### 3.5. Large-Scale Fermentation and Extraction

The *S*. sp. PH9030 strain was grown on MH16 (Table S1) agar plates and incubated at 30 °C to obtain spores. *S*. sp. PH9030 was subsequently grown in 250 mL Erlenmeyer flasks containing 50 mL sterile seed medium TSB and incubated at 30 °C on a rotary shaker (220 rpm) for 48 h. Finally, the seed culture (50 mL) was transferred into 2 L baffled Erlenmeyer flasks containing 500 mL sterile seed medium MH13 (Table S1) containing 4% microporous resins D1300 at 30 °C for 7 days. After fermentation, the culture (123 L) was filtered with EtOAc/MeOH (*v*/*v*, 1:1) (5 × 3 L) to yield D1300 resins. The EtOAc/MeOH extract was subsequently evaporated in vacuo to afford 159.4 g of crude extract.

### 3.6. Isolation of Compounds ***5***–***12***

The EtOAc/MeOH extract fraction (159.4 g) was chromatographed on silica gel columns with petroleum/EtOAc (*v*/*v*, 19:1 → 9:1 → 7:3 → 1:1 → 3:7 → 1:9), EtOAc, EtOAc/MeOH (*v*/*v*, 9:1 → 7:3 → 1:1 → 3:7 → 1:9 → 0:1) to yield twenty combined fractions (Fr. A to T). Fr. K (1.459 g) was run through an MCI column (H_2_O/MeOH, *v*/*v*, 9:1 → 4:1 → 7:3 → 3:2 → 1:1 → 2:3 → 7:13 → 3:7 → 1:3 → 1:4 → 1:9 → 0:1) to obtain five fractions altogether (Fr. K1 to K5). Fr. K3 (0.198 g) was separated by Sephadex LH-20 with MeOH as the mobile phase, obtaining three fractions altogether (Fr. K3-1 to Fr. K3-3). Fr. K3-1 (0.183 g) was run through an ODS column (H_2_O/MeOH, *v*/*v*, 9:1 → 4:1 → 7:3 → 3:2 → 1:1 → 2:3 → 7:13 → 3:7 → 1:4 → 1:9 → 0:1) to obtain six fractions altogether (Fr. K3-1-1 to Fr. K3-1-6). Fr. K3-1-4 (0.114 g) was purified using semipreparative HPLC with 50% MeCN/H_2_O (containing 0.1% formic acid) for 17 min at a flow rate of 3.0 mL/min to yield **5** (9.8 min, 2.797 mg) and **6** (11.1 min, 2.173 mg). Fr. G (10.872 g) was run through an MCI column (H_2_O/MeOH, *v*/*v*, 9:1 → 4:1 → 7:3 → 3:2 → 1:1 → 2:3 → 3:7 → 1:4 → 1:9 → 0:1) to obtain ten fractions altogether (Fr. G1 to G10). Fr. G5 (0.097 g) was run through an ODS column (H_2_O/MeOH, *v*/*v*, 9:1 → 4:1 → 7:3 → 3:2 → 1:1 → 2:3 → 3:7 → 1:4 → 1:9 → 0:1) to obtain six fractions altogether (Fr. G5-1 to Fr. G5-6). Fr. G5-4 (0.049 g) was purified using semipreparative HPLC with 70% MeCN/H_2_O (containing 0.1% formic acid) for 14 min at a flow rate of 3.0 mL/min to yield **7** (13.2 min, 9.399 mg). Fr. G5-2 (0.008 g) was purified using semipreparative HPLC with 40% MeCN/H_2_O (containing 0.1% formic acid) for 12 min at a flow rate of 3.0 mL/min to yield **9** (11.4 min, 0.952 mg). Fr. G7 (0.187 g) was run through an ODS column (H_2_O/MeOH, *v*/*v*, 9:1 → 4:1 → 7:3 → 3:2 → 1:1 → 2:3 → 3:7 → 1:4 → 1:9 → 0:1) to obtain six fractions altogether (Fr. G7-1 to Fr. G7-6). Fr. G7-1 (0.016 g) was purified using semipreparative HPLC with 50% MeCN/H_2_O (containing 0.1% formic acid) for 14 min at a flow rate of 3.0 mL/min to yield **10** (13.0 min, 0.957 mg). Fr. G7-4 (0.031 g) was purified using semipreparative HPLC with 50% MeCN/H_2_O (containing 0.1% formic acid) for 15 min at a flow rate of 3.0 mL/min to yield **11** (14.5 min, 2.300 mg). Fr. R (10.020 g) was run through an ODS column (H_2_O/MeOH, *v*/*v*, 19:1 → 9:1 → 4:1 → 7:3 → 3:2 → 1:1 → 2:3 → 3:7 → 1:4 → 1:9 → 1:19 → 0:1) to obtain ten fractions altogether (Fr. R1 to Fr. R10). Fr. R2 (0.191 g) was run through an MCI column (H_2_O/MeOH, *v*/*v*, 9:1 → 17:3 → 4:1 → 7:3 → 3:2 → 1:1 → 2:3 → 3:7 → 1:4 → 1:9 → 0:1) to obtain ten fractions altogether (Fr. R2-1 to Fr. R2-10). Fr. R2-4 (0.050 g) was separated by Sephadex LH-20 with MeOH as the mobile phase to yield **8** (6.795 mg). Fr. D (9.675 g) was run through an MCI column (H_2_O/MeOH, *v*/*v*, 19:1 → 9:1 → 4:1 → 7:3 → 3:2 → 1:1 → 2:3 → 3:7 → 1:4 → 1:9 → 0:1) to obtain three fractions altogether (Fr. D1 to D3). Fr. D3 (5.618 g) was separated by Sephadex LH-20 with MeOH as the mobile phase, obtaining three fractions altogether (Fr. D3-1 to Fr. D3-3). Fr. D3-2 (0.015 g) was purified using semipreparative HPLC with 55% MeCN/H_2_O (containing 0.1% formic acid) for 12 min at a flow rate of 3.0 mL/min to yield **12** (11.2 min, 6.830 mg).

#### Naphthgeranine G (**5**)

Yellow powder; LC-UV (MeCN/H_2_O/0.1% formic acid) λmax 213.5, 265.5, 311.9, 389.6; [α]^20.0^ _D_ −276.67 (c 0.006, MeOH); ^1^H, ^13^C and 2D NMR spectroscopic data are shown in Table 1 and Figures S4; HRESIMS *m*/*z* 355.1177 [M − H]^−^ (calcd for C_20_H_19_O_6_, 355.1187).

### 3.7. ECD Calculation Methods

The ECD spectrum of **5** was calculated via the Gaussian 09 program [44]. The B3LYP/6-31G (d) level was used to optimize those configurations. With the CPCM model in methanol solution, the ECD spectrum was computed via TDDFT at the B3LYP/6–311+ +G (2d, p) level [45]. The details are provided in the Supplementary Materials.

### 3.8. α-Glucosidase Inhibition Assay

The Worawalai technique was employed to evaluate the inhibitory activity of **5**–**12** against α-glucosidase [Sigma-Aldrich (Shanghai) Trading Co., Ltd., Shanghai, China, Product No. G5003] with minor modifications [46]. The levels of α-glucosidase were detected at 405 nm for a spectrophotometric in vitro α-glucosidase inhibitory activity test. The Supplementary Materials provide a detailed description of the reaction system.

### 3.9. Molecular Docking Analysis

The approach is outlined in the Supplementary Materials.

### 3.10. Molecular Dynamic Simulations

The approach is described in the Supplementary Materials.

### 3.11. Antibacterial Assay

The broth dilution method was used to determine the minimum inhibitory concentrations (MICs) [47]. The details are described in the Supplementary Materials.

## 4. Conclusions

In conclusion, a genus strain was identified from 30 strains of *Streptomyces* endophyticus of *K. coccinea*, named *S.* sp. PH9030. An undescribed naphthoquinone analog, naphthgeranine G (**5**), together with seven known compounds, **6**–**12,** were isolated from *S.* sp. PH9030. NMR, HRESIMS and ECD spectra were used to establish the structures of all the compounds. Naphthgeranine G (**5**), 12-hydroxy-naphthgeranine A (**6**), naphthgeranine A (**7**) and phenazine-1-carboxamide (**9**) showed α-glucosidase inhibitory activities with IC_50_ values of 66.4 ± 6.7 μM, 115.6 ± 4.4 μM, 185.9 ± 0.2 μM and 105.4 ± 10.5 μM, respectively. Molecular docking and molecular dynamics further suggest that **5** is a potential α-glucosidase inhibitor. Evaluations of their inhibitory activities against *Staphylococcus aureus* ATCC 29213, MRSA, *Klebsiella pneumoniae* ATCC 13883 and *Pseudomonas aeruginosa* ATCC 9027 revealed that **9** and **12** both exhibited moderate antibacterial activity against MRSA, with MIC values of 16 μg/mL. Considering the above results, the discovery of naphthoquinone and phenazine analogs enriches the secondary metabolites derived from endophytic *Streptomyces* of *K. coccinea* and, more importantly, provides lead compounds for the development of α-glucosidase inhibitors.

## Figures and Tables

**Table 1:** ^1^H NMR (600 MHz) and ^13^C NMR (150 MHz) data of naphthgeranine G (**5**) in DMSO-*d*_6_ (*δ* in ppm, *J* in Hz).

Position	Naphthgeranine G (5)	
	*δ*_C_, Type	*δ*_H_ (*J* in Hz)
1	178.5, C	
2	154.3, C	
3	123.0, C	
4	184.2, C	
4a	134.0, C	
5	105.9, CH	6.64 (s)
6	164.9, C	
7	104.3, CH	6.10 (d, 4.8)
8	164.8, C	
8a	107.6, C	
9	30.8, CH	3.42 (m)
10	120.1, CH	6.03 (s)
11	136.5, C	
12	65.3, CH	3.75 (m)
13	29.4, CH_2_	1.83 (dd, 2.7, 13.6); 1.23 (m)
14	33.9, CH	2.04 (ddd, 2.9, 6.2, 13.2)
15	79.8, C	
16	21.2, CH_3_	1.69 (s)
17	25.5, CH_3_	1.42 (s)
18	24.7, CH_3_	1.26 (s)

**Table 2:** α-Glucosidase inhibitory activity of the compounds **5**–**12.**

Compounds	IC_50_ (µM) ^a^	Compounds	IC_50_ (µM) ^a^
**5**	66.4 ± 6.7	**10**	>800
**6**	115.6 ± 4.4	**11**	NA ^b^
**7**	185.9 ± 0.2	**12**	NA ^b^
**8**	NA ^b^	Acarbose	671.5 ± 0.2
**9**	105.4 ± 10.5		

^a^ Data are presented as means ± SDs; ^b^ NA: not active.

**Table 3:** Logarithms of free binding energies (FBE, kcal/mol) of naphthgeranine G (**5**) and acarbose to the active cavities of α-glucosidase (PDB ID: 2QMJ) and targeting residues of the binding site located on the mobile flap.

Compound	−log (FBE)	Targeting Residues
Naphthgeranine G (**5**)	−7.2	Phe-575, Asp-542, Thr-205
		Asp-203, Arg-202, Asn-449, Tyr-299
Acarbose	−6.7	Trp-406, Tyr-299, Tyr-605
		Thr-205, Arg-526, Asp-443

## Data Availability

Data generated in the process of this research are available in the Supporting Information.

## Supplementary Materials

The following supporting information can be downloaded at: https://www.mdpi.com/article/10.3390/molecules29153450/s1, Table S1: The media used in the experiment; Table S2: The activities of 18 strains; Table S3: Gibbs free energies and equilibrium populations of low-energy conformers of **5**; Table S4: Cartesian coordinates for the low-energy reoptimized random research conformers of **5** at B3LYP-D3(BJ)/6-31G* level of theory in methanol; Table S5: Antibacterial activity (MIC, μg/mL) of **5**–**12**; Table S6: Docking output of **5**–**12** and acarbose; Table S7**:** Antibacterial activity (MIC, μg/mL) of **5**–**12**; Figure S1: HPLC analysis of the culture broths from the *Streptomyces* sp. PH9001–PH9030; Figure S2: The Antibacterial activity of *Streptomyces* sp. PH9001–PH9030; Figure S3: The partial 16S rRNA gene sequences data of *Streptomyces* sp. PH9030; Figure S4: ^1^H NMR spectrum of **5** in DMSO-*d*_6_ (600 MHz); Figure S5: ^13^C NMR spectrum of **5** in DMSO-*d*_6_ (150 MHz); Figure S6: DEPT-90 spectrum of **5**; Figure S7: DEPT-135 spectrum of **5**; Figure S8: HSQC spectrum of **5**; Figure S9: HMBC spectrum of **5**; Figure S10: ^1^H–^1^H COSY spectrum of **5**; Figure S11: NOESY spectrum of **5**; Figure S12: HRESIMS spectrum of **5**; Figure S13: UV spectrum of **5**; Figure S14: Optical rotation spectrum of **5**; Figure S15: ^1^H NMR spectrum of **6** in DMSO-*d*_6_ (600 MHz); Figure S16: ^13^C NMR spectrum of **6** in DMSO-*d*_6_ (150 MHz); Figure S17**:** DEPT-90 spectrum of **6**; Figure S18: DEPT-135 spectrum of **6**; Figure S19: HSQC spectrum of **6**; Figure S20: HMBC spectrum of **6**; Figure S21: ^1^H–^1^H COSY spectrum of **6**; Figure S22: NOESY spectrum of **6**; Figure S23: HRESIMS spectrum of **6**; Figure S24: UV spectrum of **6**; Figure S25: ^1^H NMR spectrum of **7** in DMSO-*d*_6_ (600 MHz); Figure S26: ^13^C NMR spectrum of **7** in DMSO-*d*_6_ (150 MHz); Figure S27: DEPT-135 spectrum of **7**; Figure S28: HSQC spectrum of **7**; Figure S29: HMBC spectrum of **7**; Figure S30: ^1^H–^1^H COSY spectrum of **7**; Figure S31: HRESIMS spectrum of **7**; Figure S32: UV spectrum of **7**; Figure S33: ^1^H NMR spectrum of **8** in DMSO-*d*_6_ (600 MHz); Figure S34: ^13^C NMR spectrum of **8** in DMSO-*d*_6_ (150 MHz); Figure S35: DEPT-135 spectrum of **8**; Figure S36: HSQC spectrum of **8**; Figure S37: HMBC spectrum of **8**; Figure S38: HRESIMS spectrum of **8**; Figure S39: UV spectrum of **8**; Figure S40: ^1^H NMR spectrum of **9** in DMSO-*d*_6_ (600 MHz); Figure S41: ^13^C NMR spectrum of **9** in DMSO-*d*_6_ (600 MHz); Figure S42: HRESIMS spectrum of **9**; Figure S43: UV spectrum of **9**; Figure S44: ^1^H NMR spectrum of **10** in DMSO-*d*_6_ (600 MHz); Figure S45: ^13^C NMR spectrum of **10** in DMSO-*d*_6_ (150 MHz); Figure S46: HMBC spectrum of **10**; Figure S47: HRESIMS spectrum of **10**; Figure S48: UV spectrum of **10**; Figure S49: ^1^H NMR spectrum of **11** in DMSO-*d*_6_ (500 MHz); Figure S50: ^13^C NMR spectrum of **11** in DMSO-*d*_6_ (125 MHz); Figure S51: DEPT-90 spectrum of **11**; Figure S52: DEPT-135 spectrum of **11**; Figure S53: HSQC spectrum of **11**; Figure S54: HMBC spectrum of **11**; Figure S55: ^1^H–^1^H COSY spectrum of **11**; Figure S56: NOESY spectrum of **11**; Figure S57: HRESIMS spectrum of **11**; Figure S58: UV spectrum of **11**; Figure S59: ^1^H NMR spectrum of **12** in DMSO-*d*_6_ (500 MHz); Figure S60: ^13^C NMR spectrum of **12** in DMSO-*d*_6_ (125 MHz); Figure S61: HRESIMS spectrum of **12**; Figure S62: UV spectrum of **12**; Figure S63: Docking poses and interactions of acarbose with α-glucosidase (PDB ID: 2QMJ); Figure S64: 96-well plate assay of **5**–**12** against *Staphylococcus aureus* ATCC 29213 (A), MRSA (B), *Klebsiella pneumoniae* ATCC 13883 (C) and *Pseudomonas aeruginosa* ATCC 9027 (D) using the microbroth dilution method. Refs. [48,49,50,51,52,53,54,55,56,57,58,59,60,61,62] are listed in Supporting Materials.
