# Supplementary material for: Bioactive Naphthoquinone and Phenazine Analogs from the Endophytic Streptomyces sp. PH9030 as α-Glucosidase Inhibitors
Source: Molecules. 2024 Jul 23;29(15):3450. doi: 10.3390/molecules29153450 (PMC11313965; doi:10.3390/molecules29153450)
Supplement: Supplementary file 1 [file molecules-29-03450-s001.zip › molecules-3109886-supplementary.pdf]

Supporting Information

Bioactive Naphthoquinone and Phenazine Analogs from the Endophytic *Streptomyces* sp. PH9030 as α-Glucosidase Inhibitors

Qingxian Ma [1,†], Yani Zhong [1,†], Pingzhi Huang [1,†], Aijie Li [1], Ting Jiang [2], Lin Jiang [3], Hao Yang [1], Zhong Wang [1], Guangling Wu [1], Xueshuang Huang [1,*], Hong Pu [1,*], and Jianxin Liu [1,*]

[1]China-Pakistan International Science and Technology Innovation Cooperation Base for Ethnic Medicine Development in Hunan Province, Hunan Provincial Key Laboratory for Synthetic Biology of Traditional Chinese Medicine, School of Pharmaceutical Sciences, Hunan University of Medicine, Huaihua 418000, China;

[2]Jiangxi Provincial Drug Inspection Center, Nanchang 330029, China;

[3]Hunan Engineering Technology Research Center for Bioactive Substance Discovery of Chinese Medicine, School of Pharmacy, Hunan University of Chinese Medicine, Changsha 410208, China.

***Corresponding author.**

E-mail address: xueshuanghuang@126.com (X. Huang); ph0745@126.com (H. Pu); liujianxin3385@126.com (J. Liu).

[†] These authors contributed equally to this work.

Table of Contents

Experimental Procedures 4
General experimental procedures 4
Isolation of endophytes 4
ECD calculations methods 4
α-Glucosidase inhibition assay 5
Molecular docking 5
Molecular dynamic simulations 6
Antibacterial assay 6
MM/GBSA binding free energy calculation 6
Table S1 The media used in the experiment 8
Table S2 The activities of 18 strains. 9
Table S3 Gibbs free energies[a] and equilibrium populations[b] of low-energy conformers of **5**. 10
Table S4 Cartesian coordinates for the low-energy reoptimized random reseach conformers of **5** at B3LYP-D3(BJ)/6-31G* level of theory in methanol. 11
Table S5 Binding free energies and energy components predicted by MM/GBSA (kcal/mol). 19
Table S6 Docking output of compounds **5** – **12** and acarbose. 20
Table S7 Antibacterial activity (MIC, μg/mL) of compounds **5** – **12**. 21
Figure S1 HPLC analysis of the culture broths from the *Streptomyces* sp. PH9001 – PH9030 22
Figure S2 The Antibacterial activity of *Streptomyces* sp. PH9001 – PH9030 23
Figure S3 The partial 16S rRNA gene sequences data of *Streptomyces* sp. PH9030 24
Figure S4 ^{1}H NMR spectrum of **5** in DMSO-*d6* (600 MHz) 25
Figure S5 ^{13}C NMR spectrum of **5** in DMSO-*d6* (150 MHz) 26
Figure S6 DEPT-90 spectrum of **5** 27
Figure S7 DEPT-135 spectrum of **5** 28
Figure S8 HSQC spectrum of **5** 29
Figure S9 HMBC spectrum of **5** 30
Figure S10 ^{1}H-^{1}H COSY spectrum of **5** 31
Figure S11 NOESY spectrum of **5** 32
Figure S12 HRESIMS spectrum of **5** 33
Figure S13 UV spectrum of **5** 34
Figure S14 Optical rotation spectrum of **5** 35
Figure S15 ^{1}H NMR spectrum of **6** in DMSO-d_6 (600 MHz) 36
Figure S16 ^{13}C NMR spectrum of **6** in DMSO-d_6 (150 MHz) 37
Figure S17 DEPT-90 spectrum of **6** 38
Figure S18 DEPT-135 spectrum of **6** 39
Figure S19 HSQC spectrum of **6** 40
Figure S20 HMBC spectrum of **6** 41
Figure S21 ^{1}H-^{1}H COSY spectrum of **6** 42
Figure S22 NOESY spectrum of **6** 43
Figure S23 HRESIMS spectrum of **6** 44
Figure S24 UV spectrum of **6** 45
Figure S25 ^{1}H NMR spectrum of **7** in DMSO-d_6 (600 MHz) 46
Figure S26 ^{13}C NMR spectrum of **7** in DMSO-d_6 (150 MHz) 47
Figure S27 DEPT-135 spectrum of **7** 48
Figure S28 HSQC spectrum of **7** 49
Figure S29 HMBC spectrum of **7** 50
Figure S30 ^{1}H-^{1}H COSY spectrum of **7** 51
Figure S31 HRESIMS spectrum of **7** 52
Figure S32 UV spectrum of **7** 53
Figure S33 ^{1}H NMR spectrum of **8** in DMSO-d_6 (600 MHz) 54
Figure S34 ^{13}C NMR spectrum of **8** in DMSO-d_6 (150 MHz) 55
Figure S35 DEPT-135 spectrum of **8** 56
Figure S36 HSQC spectrum of **8** 57
Figure S37 HMBC spectrum of **8** 58
Figure S38 HRESIMS spectrum of **8** 59
Figure S39 UV spectrum of **8** 60
Figure S40 ^{1}H NMR spectrum of **9** in DMSO-d_6 (600 MHz) 61

Figure S41 ^{13}C NMR spectrum of **9** in DMSO-d_6 (600 MHz) 62
Figure S42 HRESIMS spectrum of **9** 63
Figure S43 UV spectrum of **9** 64
Figure S44 ^{1}H NMR spectrum of **10** in DMSO-d_6 (600 MHz) 65
Figure S45 ^{13}C NMR spectrum of **10** in DMSO-d_6 (150 MHz) 66
Figure S46 HMBC spectrum of **10** 67
Figure S47 HRESIMS spectrum of **10** 68
Figure S48 UV spectrum of **10** 69
Figure S49 ^{1}H NMR spectrum of **11** in DMSO-d_6 (500 MHz) 70
Figure S50 ^{13}C NMR spectrum of **11** in DMSO-d_6 (125 MHz) 71
Figure S51 DEPT-90 spectrum of **11** 72
Figure S52 DEPT-135 spectrum of **11** 73
Figure S53 HSQC spectrum of **11** 74
Figure S54 HMBC spectrum of **11** 75
Figure S56 NOESY spectrum of **11** 77
Figure S57 HRESIMS spectrum of **11** 78
Figure S58 UV spectrum of **11** 79
Figure S59 ^{1}H NMR spectrum of **12** in DMSO-d_6 (500 MHz) 80
Figure S60 ^{13}C NMR spectrum of **12** in DMSO-d_6 (125 MHz) 81
Figure S61 HRESIMS spectrum of **12** 82
Figure S62 UV spectrum of **12** 83
Figure S63 Docking poses and interactions of acarbose with α-glucosidase (PDB ID: 2QMJ) 84
Figure S64 96-well plate assay of **5 – 12** against *Staphylococcus aureus* ATCC 29213 (A), MRSA (B), *Klebsiella pneumoniae* ATCC 13883 (C) and *Pseudomonas aeruginosa* ATCC 9027 (D) using the microbroth dilution method. 85

Experimental Procedures

General experimental procedures

Extract genomic DNA though Ezup Column Bacteria Genomic DNA Purification Kit [Sangon Biotech (Shanghai) Co., Ltd., Shanghai, China]. Microporous resin D1300 (Tianjin Haoju Resin Technology Co., LTD., Tianjin, China). UV spectra were measured on a Waters 2998 PDA Detector [Waters Technology (Shanghai) Co., Ltd., Shanghai, China]. Semipreparative reversed-phase high performance liquid chromatography (RP- HPLC) was performed using a Waters 1525 Binary HPLC pump equipped with a Waters 2489 UV/visible detector [Waters Technology (Shanghai) Co., Ltd., Shanghai, China] and using a Welch Ultimate AQ-C18 column (250 × 10 mm, 5 μm) [Welch Technology (shanghai) Co., Ltd., Shanghai, China]. HRESIMS spectra were recorded on a Q-Exactive Focus Orbitrap MS (Thermo Electron, Bremen, Germany) connected to the Thermo Scientific Dionex Ultimate 3000 RS (Thermo Fisher Scientific, California, USA). NMR spectra were acquired using a Bruker 500 MHz or 600 MHz spectrometer (Bruker Corporation, Massachusetts, USA). The Chemical shifts in ^{1}H NMR and ^{13}C NMR spectra were referenced to the solvents for DMSO-d_6 (δ_H 2.50 and δ_C 39.6) (Cambridge Isotope Laboratories, Inc., Massachusetts, USA). Column chromatography (CC) was carried out on silica gel (200–300 mesh, Yantai Jiangyou Silica Gel Development Co., Ltd., Yantai, China), MCI GEL CHP20/P120 (Mitsubishi Chemical Group Corporation, Tokyo, Japan), RP-C18 (AAG12S50, YMC Co., Ltd., Kyoto, Japan) and Sephadex LH-20 (Cytiva Sweden AB SE-751 84 Uppsala, Sweden).

Isolation of endophytes

Disinfection of plant materials Fresh roots of *Kadsura coccinea* (Lem.) A. C. Smith were first rinsed with tap water after necrotic rhizomes were removed. Next, they were soaked in detergent (3 min) and rinsed with distilled water (20 min). After washed with sterile water, the prepared roots were cut into 5-8 mm sections in a biosafety hood. These plant sections were next soaked in 75% ethanol (1 min), washed with sterile water, soaked in 15% sodium hypochlorite solution (15 min), washed with sterile water.

Isolation of endophytic actinomycetes The isolation medium was augmented with nystatin 50 mg/L and nalidixic acid 25 mg/L, to prevent the development of fungus and bacteria. The rhizosphere soil of *K. coccinea* were ground and crushed in a mortar with a pestle on a sterile laboratory bench. The resulting mixture was made into a homogenate by adding approximately 5 mL of sterile distilled water. The homogenate was then aspirated and diluted into a suspension using gradients of 10^{-1}, 10^{-2}, and 10^{-3} fold. Finally, 0.2 mL of each gradient was applied to three different selective isolation media (MH14, MH15, MH16, **Table S1**). The isolation medium was incubated at a temperature of 30 °C for a duration of 7 days with regular inversion. Subsequently, it was moved to room temperature. Throughout this period, daily observations were conducted, and individual colonies were selected based on their growth and morphological characteristics. These selected colonies were then purified under the same temperature and on the same medium. The colonies were scrutinized and chosen based on their distinct attributes and physical appearance. The refined strains were preserved as glycerol suspensions in water (20%, v/v) at a temperature of -80 °C.

ECD calculations methods

In general, conformational analyses were performed for (9*R*, 12*S*, 14*S*)-**5** and (9*S*, 12*R*, 14*R*)-**5** via random searching in the Sybyl-X 2.0 using the MMFF94S force field with an energy cutoff of 5 kcal/moL[1]. The results

showed eight lowest energy conformers for (9*R*, 12*S*, 14*S*)-**5** and (9*S*, 12*R*, 14*R*)-**5**, respectively. Subsequently, geometry optimizations and frequency analyses were implemented at the B3LYP-D3(BJ)/6-31G* level in CPCM methanol using ORCA5.0.1[2]. All conformers used for property calculations in this work were characterized to be stable point on potential energy surface (PES) with no imaginary frequencies. The excitation energies, oscillator strengths, and rotational strengths (velocity) of the first 60 excited states were calculated using the TD-DFT methodology at the PBE0/def2-TZVP level in CPCM methanol using ORCA5.0.1[2]. The ECD spectra were simulated by the overlapping Gaussian function (half the bandwidth at 1/e peak height, sigma = 0.30 for all)[3]. Gibbs free energies for conformers were determined by using thermal correction at B3LYP-D3(BJ)/6-31G* level and electronic energies evaluated at the wB97M-V/def2-TZVP level in CPCM methanol using ORCA5.0.1[2]. To get the final spectra, the simulated spectra of the conformers were averaged according to the boltzmann distribution theory and their relative Gibbs free energy (ΔG). The comparison of the experimental ECD spectrum of **5** with the calculated ones revealed that the Cotton effects (CEs) of (9*R*, 12*S*, 14*S*)-**5** were in good accordance with the experimental CEs of **5** in the region 200~400 nm.

α-Glucosidase inhibition assay

The inhibitory activity of compounds **5** – **12** against α-glucosidase [Sigma-Aldrich (Shanghai) Trading Co., Ltd., Shanghai, China, Product No. G5003] was investigated according to the method of Worawalai with a slight modification[4]. Those compounds were dissolved in DMSO (Shanghai Macklin Biochemical Technology Co., Ltd., Shanghai, China). α-glucosidase enzyme and 4-Nitrophenyl α-D-glucopyranoside (PNPG) (Shanghai Aladdin Biochemical Technology Co., Ltd., Shanghai, China) were dissolved in 0.1 M potassium phosphate buffer (Shanghai Aladdin Biochemical Technology Co., Ltd., Shanghai, China) (pH 6.8) independently. Four groups were set up, namely the test group with the test compound and the enzyme, control group with only the enzyme, sample group with only the test compound, and blank group without anything, while every group was added with potassium phosphate buffer and PNPG to build a reaction system of 200 μL. In the test group, 10 μL of α-glucosidase (final concentration 0.1 U/mL), 130 μL of phosphate buffer and 10 μL of the test compounds were added into 96 well plates (Corning Incorporated, New York, USA), in succession. After incubated at 37 °C for 20 min, 50 μL of PNPG (final concentration 1 mmol/L) was added into the mixture, followed by incubation at 37 °C for 40 min. Finally, 100 μL of Na_2CO_3 (0.5 M) (Shanghai Aladdin Biochemical Technology Co., Ltd., Shanghai, China) was added into the mixture to stop reaction. The enzymatic activity was quantified by measuring the absorbance at 405 nm using multi-mode microplate reader (BMG Labtech, Ortenberg, Germany). The inhibition percentage was calculated as follows: % of inhibition = $[1-(A_s-A_t)/(A_c-A_b)] \times 100\%$, where As is the absorbance of the sample group; At is the absorbance of the test group; Ac is the absorbance of the control group; Ab is the absorbance of the blank group.

Molecular docking

Molecular docking studies were performed to investigate the binding mechanism between the naphthgeranine G (**5**) and the N-terminal subunit of Human Maltase-Glucoamylase in Complex with acarbose (PDB ID: 2QMJ) using Autodock vina 1.1.2 [5]. The AutoDockTools 1.5.6 package[6,7] had been used for generating the docking input documents. The search grid of a-glucosidase was identified as center_x: -21.727, center_y: -6.323, and center_z: -5.281 with dimensions size_x: 15, size_y: 15, and size_z: 16.5. Vina docking generally used default parameters unless otherwise indicated. The optimal scoring pose was determined by the Vina docking score and PyMOL 1.8

software (https://www.pymol.org) was utilized to make visual analysis.

Molecular dynamic simulations

Molecular dynamics simulations were performed using AMBER 18 software for simulation [8]. Before the simulation, the system was energy optimized, including the steepest descent method with 2500 steps and the conjugate gradient method with 2500 steps. After the energy optimization of the system was completed, the temperature of the system was slowly increased from 0 K to 298.15 K by heating the system for 200 ps at a fixed volume and constant heating speed. Under the condition of the system maintenance temperature of 298.15 K, the NVT (isothermal isobody) system simulation was performed for 500 ps, so that the solvent molecules were further evenly distributed in the solvent box. Finally, the equilibrium simulation of the whole system was carried out for 500 ps in the case of NPT (isothermal and isopressure). Finally, the NPT (isothermal and isopressure) system simulation was carried out for 100 ns under periodic boundary conditions for the two composite systems. For simulations, the nonbonded cutoff distance was set to 10 Å, the Particle mesh Ewald (PME) method was used to calculate long-range electrostatic interactions[9], the SHAKE method was used to limit the length of hydrogen atomic bonds[10], and the Langevin algorithm was used for temperature control[11], where the collision frequency γ was set to 2 ps^{-1}. The system pressure was 1 atm, the integration step was 2 fs, and the trajectories were saved at 10 ps intervals for subsequent analysis.

Antibacterial assay

Compounds **5 – 12** were tested for antibacterial activity against *Staphylococcus aureus* ATCC 29213, methicillin-resistant *Staphylococcus aureus* (MRSA), *Klebsiella pneumoniae* ATCC 13883 and *Pseudomonas aeruginosa* ATCC 9027. The MICs were determined using broth dilution assay. The bacterial strains were cultured overnight and diluted to 106 CFU/mL in Luria-Bertani (LB, **Table S1**) broth. **5 – 12** were dissolved in DMSO, serially diluted to 10 different concentrations (64, 32, 16, 8, 4, 2, 1, 0.5. 0.25, 0.125 μg/mL) using LB broth on the 96 well plate. Levofloxacin (Bide Pharmatech Ltd., Shanghai, China) was dissolved in DMSO, serially diluted to 10 concentrations (0.125–64 μg/mL) using LB broth on each 96-well plate. Then 100 μL LB broth containing compounds **5 – 12** or levofloxacin and 100 μL of bacterial solution were mixed per well. The plates were incubated at 37 °C for 18 h. Finally, 50 μL resazurin (Shanghai Macklin Biochemical Technology Co., Ltd., Shanghai, China) was added into each well to visualize the result. **5 – 12** and levofloxacin were tested in duplicate on each 96 well plate.

MM/GBSA binding free energy calculation

The binding free energies between protein and ligand for all systems were calculated by the MM/GBSA method [12–15]. Long time molecular dynamics simulation may not be conducive to the accuracy of MM/GBSA calculation[12], so in this study, the MD trajectory of 45-50 ns is used for calculation, and the specific formula is as follows:

$$\Delta G_{bind} = \Delta G_{complex} - (\Delta G_{receptor} + \Delta G_{ligand}) = \Delta E_{internal} + \Delta E_{VDW} + \Delta E_{elec} + \Delta G_{GB} + \Delta G_{SA} \quad (1)$$

In equation (1), denotes the internal energy, denotes the van der Waals action and denotes the electrostatic interaction. The internal energy includes the bond energy (Ebond), the Angle energy (Eangle) and the torsion energy (Etorsion). And are collectively referred to as solvation free energies. Here, GGB is the polar solvation free energy

and GSA is the non-polar solvation free energy. For, this paper uses the GB model developed by Nguyen et al. [16]for calculation (igb = 2). The non-polar solvation free energy (GSA) was calculated based on the product of the surface tension (γ) and the solvent accessible surface area (SA), GSA= 0.0072 × SASA[17]. Entropy change is ignored in this study due to high computational resource consumption and low accuracy[12,13].

Table S1 The media used in the experiment.

Media	Compositions
MH13	soluble starch [a] 20 g/L, sucrose [b] 5 g/L, fish meal [e] 20 g/L, $CuSO_4·5H_2O$ [b] 0.1 g/L, NaI [a] 0.005 g/L, $CaCO_3$ [b] 2 g/L
MH14	soluble starch [a] 20 g/L, NaCl [b] 0.5 g/L, KNO_3 [b] 1 g/L, $K_2HPO_4·3H_2O$ [b] 0.5 g/L, $MgSO_4·7H_2O$ [b] 0.5 g/L, $FeSO_4·7H_2O$ [b] 0.01 g/L, agar powder [c] 20 g/L
MH15	soluble starch [a] 10 g/L, $K_2HPO_4·3H_2O$ [b] 1 g/L, $MgSO_4·7H_2O$ [b] 1 g/L, NaCl [b] 1 g/L, $(NH_4)_2SO_4$ [b] 2 g/L, $CaCO_3$ [b] 2 g/L, $FeSO_4·7H_2O$ [b] 0.001 g/L, $MnCl_2·4H_2O$ [b] 0.001 g/L, agar powder [c] 20 g/L
MH16	glucose [b] 0.5 g/L, yeast extract [d] 0.5 g/L, soya peptone [d] 0.5 g/L, soluble starch [a] 0.5 g/L, caseein hydrolysate [c] 0.5 g/L, sodium pyruvate [b] 0.5 g/L, $K_2HPO_4·3H_2O$ [b] 0.3 g/L, $MgSO_4·7H_2O$ [b] 0.024 g/L, agar powder [c] 20 g/L
MH18	soluble starch [a] 20 g/L, corn flour [f] 20 g/L, KH_2PO_4 [b] 0.5 g/L, $MgSO_4·7H_2O$ [b] 0.25 g/L, $ZnSO_4·7H_2O$ [b] 0.001 g/L, $FeSO_4·7H_2O$ [b] 0.001 g/L, $MnCl_2·4H_2O$ [b] 0.001 g/L, $CaCl_2$ 0.001 [b] g/L, $CaCO_3$ [b] 5 g/L
LB	yeast extract [d] 5 g/L, tryptone [d] 10 g/L, NaCl [b] 10 g/L

[a] Tianjin Kemiou Chemical Reagent Co., Ltd., Tianjin, China

[b] Sinopharm Chemical Reagent Co., Ltd., Shanghai, China

[c] Guangdong Huankai Microbial Sci. & Tech. Co., Ltd., Guangzhou, China

[d] Thermo Scientific Oxoid, Massachusetts, USA

[e] Consorcio Malla S.A., Peru

[f] Hunan Jiahui Department Store Co., Ltd., Huaihua, China

Table S2 The activities of 18 strains.

Strain	*S. aureus* ATCC 29213	MRSA	*P. aeruginosa* ATCC 9027	Strain	*S. aureus* ATCC 29213	MRSA	*P. aeruginosa* ATCC 9027
PH9001	-	+	+	PH9015	-	+	+
PH9002	+	-	-	PH9016	-	-	+
PH9003	-	+	-	PH9018	-	-	-
PH9006	++	+	+	PH9019	-	-	-
PH9008	-	-	+	PH9021	++	++	+
PH9009	-	-	+	PH9022	-	-	-
PH9010	-	-	+	PH9028	-	-	-
PH9013	++	++	+	PH9029	++	++	-
PH9014	++	++	+	PH9030	++++	+++	-

Diameter of inhibitory zone: “-”: no inhibitory zone; “+”: 1~8 mm width of inhibition zone; “++”: 9~15 mm width of inhibition zone; “+++”: 16~20 mm width of inhibition zone; “++++”: more than 20 mm width of inhibition zone.

Table S3 Gibbs free energies[a] and equilibrium populations[b] of low-energy conformers of **5**.

Conformers	ΔG(a.u.)	P(%)/100	G(a.u.)
napG000001_tddft_	0.00000	19.58	-1225.296539
napG000002_tddft_	0.00001	19.46	-1225.296533
napG000003_tddft_	0.00016	16.6	-1225.296383
napG000004_tddft_	0.00036	13.34	-1225.296176
napG000005_tddft_	0.00240	1.55	-1225.294143
napG000006_tddft_	0.00229	1.73	-1225.294245
napG000007_tddft_	0.00016	16.45	-1225.296375
napG000008_tddft_	0.00052	11.31	-1225.29602

[a]wB97M-V/def2-TZVP, in a.u.

[b]From ΔG values at 298.15K.

Table S4 Cartesian coordinates for the low-energy reoptimized random reseach conformers of **5** at B3LYP-D3(BJ)/6-31G* level of theory in methanol.

napG000001_en_		Standard Orientation (A.U.)			
Center number	Atomic number	Atomic Type	X	Y	Z
1	6	C	-11.993191	-4.675609	-1.889981
2	6	C	-9.628232	-3.421217	-1.667965
3	6	C	-7.385163	-4.742241	-2.287422
4	6	C	-7.463973	-7.223943	-3.10169
5	6	C	-9.822629	-8.45664	-3.314868
6	6	C	-12.060017	-7.198761	-2.717745
7	6	C	-9.556841	-0.819221	-0.799593
8	6	C	-7.01423	0.41111	-0.568145
9	6	C	-4.827974	-0.816826	-1.164312
10	6	C	-4.874934	-3.435917	-2.098347
11	8	O	-7.153681	2.781834	0.253787
12	6	C	-4.843981	3.995027	1.160929
13	6	C	-2.734918	3.410565	-0.784439
14	6	C	-2.323879	0.552783	-1.125984
15	6	C	-0.183951	4.713036	-0.366187
16	6	C	1.402296	3.679081	1.825043
17	6	C	1.241817	0.825883	2.057514
18	6	C	-0.436276	-0.535856	0.730732
19	6	C	3.044441	-0.36083	3.906131
20	6	C	-5.494998	6.80011	1.197062
21	6	C	-4.441004	3.005697	3.849652
22	8	O	-2.924364	-4.513298	-2.724274
23	8	O	-11.484751	0.380495	-0.245468
24	8	O	-14.149499	-3.520003	-1.332805
25	8	O	-9.790157	-10.873883	-4.116809
26	1	H	-1.509803	0.248525	-3.029342
27	1	H	-3.501335	4.141502	-2.57903
28	8	O	3.945762	4.52599	1.616873
29	1	H	-5.721883	-8.211563	-3.580532
30	1	H	-13.896447	-8.128849	-2.875312
31	1	H	-0.386812	6.771355	-0.167572
32	1	H	0.928482	4.410925	-2.105566
33	1	H	0.763245	4.512837	3.62687
34	1	H	-0.4543	-2.592258	0.921529
35	1	H	5.014732	0.133844	3.457443
36	1	H	2.694952	0.364321	5.832626
37	1	H	2.853979	-2.429204	3.940375
38	1	H	-7.230615	7.101204	2.297973
39	1	H	-3.963381	7.898362	2.069349
40	1	H	-5.813335	7.509451	-0.730814
41	1	H	-6.15712	3.384643	4.958968
42	1	H	-4.071671	0.966289	3.877662
43	1	H	-2.849841	3.960599	4.774277
44	1	H	-13.696491	-1.773282	-0.790464
45	1	H	-11.495332	-11.525757	-4.19804
46	1	H	4.650934	3.776932	0.104783

napG000002_en_		Standard Orientation (A.U.)			
Center number	Atomic number	Atomic Type	X	Y	Z
1	6	C	-12.055058	-4.685815	-1.747888
2	6	C	-9.688667	-3.427202	-1.571262
3	6	C	-7.458998	-4.733115	-2.265152
4	6	C	-7.551946	-7.205186	-3.106914
5	6	C	-9.911517	-8.442988	-3.272735
6	6	C	-12.136413	-7.199277	-2.60308
7	6	C	-9.600344	-0.83809	-0.665755
8	6	C	-7.054455	0.391373	-0.47428
9	6	C	-4.880359	-0.819068	-1.145348
10	6	C	-4.947506	-3.421827	-2.125405
11	8	O	-7.175002	2.744242	0.401551
12	6	C	-4.846547	3.927698	1.297248
13	6	C	-2.775465	3.403037	-0.705846
14	6	C	-2.37828	0.55579	-1.140282
15	6	C	-0.221498	4.703271	-0.304825
16	6	C	1.408649	3.608329	1.806749
17	6	C	1.301586	0.74243	1.906649
18	6	C	-0.426244	-0.584934	0.614009
19	6	C	3.238762	-0.510882	3.565746
20	6	C	-5.496522	6.729782	1.436534
21	6	C	-4.386507	2.853187	3.944396
22	8	O	-3.013664	-4.485654	-2.821356
23	8	O	-11.516873	0.34886	-0.047932
24	8	O	-14.200014	-3.543499	-1.121798
25	8	O	-9.893272	-10.850861	-4.103287
26	1	H	-1.617013	0.308212	-3.073257
27	1	H	-3.577758	4.1865	-2.461968
28	8	O	3.903973	4.516748	1.357838
29	1	H	-5.819902	-8.180773	-3.643382
30	1	H	-13.973704	-8.133165	-2.723707
31	1	H	-0.434633	6.751151	-0.027455
32	1	H	0.88577	4.464088	-2.051801
33	1	H	0.758529	4.338009	3.661754
34	1	H	-0.41267	-2.647452	0.715639
35	1	H	3.132516	0.207398	5.525421
36	1	H	2.98144	-2.571044	3.621379
37	1	H	5.166495	-0.123257	2.872976
38	1	H	-3.945098	7.801185	2.306783
39	1	H	-5.858585	7.498603	-0.46046
40	1	H	-7.206907	6.993904	2.585474
41	1	H	-6.084878	3.18022	5.096688
42	1	H	-3.997636	0.817618	3.897738
43	1	H	-2.785587	3.793245	4.867425
44	1	H	-13.737329	-1.802175	-0.570264
45	1	H	-11.598411	-11.506241	-4.148382
46	1	H	4.904559	4.233717	2.854968

napG000003_en_		Standard Orientation (A.U.)			
Center number	Atomic number	Atomic Type	X	Y	Z
1	6	C	-12.165569	-4.488953	-1.87961
2	6	C	-9.762922	-3.298109	-1.646341
3	6	C	-7.557278	-4.688571	-2.222217
4	6	C	-7.709756	-7.180613	-3.004635
5	6	C	-10.099842	-8.347701	-3.225909
6	6	C	-12.304945	-7.016434	-2.671648
7	6	C	-9.614261	-0.688185	-0.808986
8	6	C	-7.032375	0.462564	-0.567583
9	6	C	-4.879477	-0.839618	-1.128223
10	6	C	-5.007005	-3.466072	-2.026719
11	8	O	-7.104604	2.844192	0.226458
12	6	C	-4.766344	3.996333	1.140977
13	6	C	-2.66045	3.325911	-0.780108
14	6	C	-2.334182	0.452775	-1.086606
15	6	C	-0.075293	4.556377	-0.353683
16	6	C	1.460714	3.499357	1.861936
17	6	C	1.216666	0.654086	2.119427
18	6	C	-0.492021	-0.67111	0.794571
19	6	C	2.971792	-0.566629	3.99137
20	6	C	-5.331983	6.820031	1.142755
21	6	C	-4.415188	3.023551	3.842754
22	8	O	-3.090866	-4.624759	-2.619124
23	8	O	-11.503574	0.582189	-0.288398
24	8	O	-14.288662	-3.255233	-1.361296
25	8	O	-10.334071	-10.771861	-3.977001
26	1	H	-1.51684	0.103961	-2.981053
27	1	H	-3.389692	4.059232	-2.589136
28	8	O	4.028602	4.272387	1.670922
29	1	H	-5.963448	-8.190944	-3.443337
30	1	H	-14.143275	-7.927848	-2.848861
31	1	H	-0.219566	6.621621	-0.177288
32	1	H	1.042587	4.203982	-2.08015
33	1	H	0.827973	4.367508	3.64961
34	1	H	-0.571057	-2.724141	1.005401
35	1	H	2.725362	-2.628807	4.039303
36	1	H	4.958035	-0.128125	3.55505
37	1	H	2.626549	0.182167	5.909514
38	1	H	-3.777212	7.879858	2.021489
39	1	H	-5.607909	7.520002	-0.795047
40	1	H	-7.069452	7.184725	2.221339
41	1	H	-6.128671	3.462468	4.933882
42	1	H	-4.104841	0.974829	3.894954
43	1	H	-2.804956	3.942284	4.770924
44	1	H	-13.792664	-1.518544	-0.837455
45	1	H	-8.679661	-11.475277	-4.302787
46	1	H	4.725235	3.495087	0.169207

napG000004_en_		Standard Orientation (A.U.)			
Center number	Atomic number	Atomic Type	X	Y	Z
1	6	C	-12.107481	-4.604755	-1.952374
2	6	C	-9.723038	-3.394279	-1.699758
3	6	C	-7.493217	-4.776188	-2.225292
4	6	C	-7.603386	-7.275389	-2.980627
5	6	C	-9.981353	-8.46375	-3.226866
6	6	C	-12.206448	-7.145806	-2.719639
7	6	C	-9.618484	-0.772605	-0.897229
8	6	C	-7.058465	0.412527	-0.637847
9	6	C	-4.882197	-0.875674	-1.131774
10	6	C	-4.960772	-3.518867	-1.997181
11	8	O	-7.171129	2.811544	0.106482
12	6	C	-4.867105	3.99886	1.054083
13	6	C	-2.707524	3.316126	-0.802788
14	6	C	-2.351302	0.44203	-1.056069
15	6	C	-0.140415	4.570282	-0.333453
16	6	C	1.356461	3.553063	1.92601
17	6	C	1.169567	0.712121	2.179506
18	6	C	-0.5303	-0.636931	0.870755
19	6	C	2.967535	-0.478198	4.026824
20	6	C	-5.46213	6.816402	0.9938
21	6	C	-4.56807	3.075918	3.779266
22	8	O	-3.019396	-4.655568	-2.537952
23	8	O	-11.534299	0.479044	-0.419764
24	8	O	-14.251691	-3.390199	-1.479945
25	8	O	-9.979857	-10.900559	-3.96866
26	1	H	-1.489774	0.072814	-2.92657
27	1	H	-3.393956	4.01634	-2.641444
28	8	O	3.958823	4.195537	1.688084
29	1	H	-5.870598	-8.310435	-3.387739
30	1	H	-14.057343	-8.041952	-2.903044
31	1	H	-0.333975	6.638831	-0.187454
32	1	H	1.046249	4.203698	-2.004358
33	1	H	0.628062	4.38755	3.707132
34	1	H	-0.588265	-2.687562	1.104309
35	1	H	2.736995	0.355047	5.927115
36	1	H	2.666672	-2.529013	4.166695
37	1	H	4.935744	-0.114192	3.460543
38	1	H	-7.232078	7.179148	2.019092
39	1	H	-3.944474	7.909523	1.896779
40	1	H	-5.693682	7.481351	-0.962176
41	1	H	-4.225162	1.033679	3.871472
42	1	H	-2.993172	4.033199	4.729104
43	1	H	-6.313713	3.504146	4.822845
44	1	H	-13.774018	-1.640566	-0.968297
45	1	H	-11.696069	-11.518122	-4.079303
46	1	H	4.101799	6.011678	1.790426

napG000005_en_		Standard Orientation (A.U.)			
Center number	Atomic number	Atomic Type	X	Y	Z
1	6	C	-11.873266	-4.262772	-2.353556
2	6	C	-9.51293	-3.062117	-1.872461
3	6	C	-7.27149	-4.510415	-1.968791
4	6	C	-7.348485	-7.066574	-2.522411
5	6	C	-9.697517	-8.241616	-2.992662
6	6	C	-11.936659	-6.855545	-2.90993
7	6	C	-9.446606	-0.383815	-1.265562
8	6	C	-6.913474	0.770015	-0.728893
9	6	C	-4.726213	-0.586155	-0.834898
10	6	C	-4.758952	-3.285554	-1.493487
11	8	O	-7.05269	3.209799	-0.146513
12	6	C	-4.85722	4.407756	1.012367
13	6	C	-2.459083	3.579693	-0.438411
14	6	C	-2.197998	0.658167	-0.475028
15	6	C	-0.046377	4.915547	0.537594
16	6	C	2.209855	3.142355	0.88764
17	6	C	1.436363	0.856657	2.440978
18	6	C	-0.697301	-0.321801	1.753044
19	6	C	3.087716	0.058214	4.603363
20	6	C	-5.36629	7.225623	0.695649
21	6	C	-4.880916	3.643087	3.80368
22	8	O	-2.796277	-4.503061	-1.655541
23	8	O	-11.372046	0.934342	-1.15932
24	8	O	-14.029107	-2.978667	-2.283213
25	8	O	-9.85932	-10.727347	-3.534717
26	1	H	-1.065992	0.201562	-2.162398
27	1	H	-2.780864	4.162115	-2.40845
28	8	O	3.076348	2.459692	-1.574643
29	1	H	-5.573962	-8.120057	-2.58758
30	1	H	-13.743638	-7.774144	-3.274255
31	1	H	-0.398012	5.817564	2.376257
32	1	H	0.513727	6.429095	-0.772156
33	1	H	3.720781	4.188584	1.890003
34	1	H	-1.352182	-2.017334	2.733452
35	1	H	3.245475	1.574849	6.029301
36	1	H	2.352128	-1.642612	5.54457
37	1	H	5.033827	-0.347611	3.965217
38	1	H	-5.388964	7.737239	-1.318961
39	1	H	-7.217429	7.699757	1.51165
40	1	H	-3.912705	8.356962	1.654429
41	1	H	-6.636156	4.318704	4.687607
42	1	H	-4.78358	1.582456	4.028301
43	1	H	-3.269754	4.464711	4.821951
44	1	H	-13.585752	-1.201652	-1.855579
45	1	H	-8.187109	-11.463173	-3.539544
46	1	H	4.059098	0.928521	-1.426595

napG000006_en_		Standard Orientation (A.U.)			
Center number	Atomic number	Atomic Type	X	Y	Z
1	6	C	-11.94249	-4.569861	-2.157125
2	6	C	-9.614845	-3.311282	-1.697062
3	6	C	-7.320972	-4.650093	-2.019169
4	6	C	-7.313973	-7.152348	-2.772593
5	6	C	-9.63618	-8.38897	-3.223477
6	6	C	-11.922577	-7.114213	-2.920457
7	6	C	-9.634542	-0.687395	-0.889894
8	6	C	-7.133097	0.547969	-0.407465
9	6	C	-4.899023	-0.69448	-0.71791
10	6	C	-4.844728	-3.342845	-1.566522
11	8	O	-7.348732	2.938185	0.339333
12	6	C	-5.148116	4.167813	1.450791
13	6	C	-2.800055	3.542112	-0.172662
14	6	C	-2.413015	0.644372	-0.412636
15	6	C	-0.397773	4.926151	0.760698
16	6	C	1.953263	3.247142	0.847762
17	6	C	1.382107	0.830679	2.287974
18	6	C	-0.738166	-0.401563	1.655095
19	6	C	3.206789	-0.029671	4.280396
20	6	C	-5.794347	6.972992	1.335242
21	6	C	-4.982007	3.240107	4.186698
22	8	O	-2.839182	-4.445511	-1.903435
23	8	O	-11.610668	0.523224	-0.583659
24	8	O	-14.144311	-3.398179	-1.8813
25	8	O	-9.521585	-10.828511	-3.950455
26	1	H	-1.363192	0.350612	-2.186904
27	1	H	-3.253574	4.22867	-2.082123
28	8	O	2.688575	2.771296	-1.704283
29	1	H	-5.531191	-8.152589	-3.019021
30	1	H	-13.732538	-8.04703	-3.261903
31	1	H	-0.678213	5.672672	2.679446
32	1	H	0.00871	6.557415	-0.461497
33	1	H	3.47808	4.297051	1.825076
34	1	H	-1.254166	-2.186927	2.55567
35	1	H	5.119069	-0.30649	3.48936
36	1	H	3.400837	1.397792	5.7916
37	1	H	2.60674	-1.81871	5.151645
38	1	H	-7.615178	7.315144	2.276194
39	1	H	-4.337231	8.110835	2.280883
40	1	H	-5.952376	7.600545	-0.640162
41	1	H	-4.745438	1.180469	4.280178
42	1	H	-3.377056	4.104428	5.179069
43	1	H	-6.728905	3.746167	5.192444
44	1	H	-13.7481	-1.639186	-1.332528
45	1	H	-11.209464	-11.47884	-4.209398
46	1	H	3.736201	1.276638	-1.725465

napG000007_en_		Standard Orientation (A.U.)			
Center number	Atomic number	Atomic Type	X	Y	Z
1	6	C	-12.113625	-4.385642	-1.848693
2	6	C	-9.704702	-3.204116	-1.636505
3	6	C	-7.508046	-4.609884	-2.208053
4	6	C	-7.675422	-7.108062	-2.967566
5	6	C	-10.071646	-8.265748	-3.169009
6	6	C	-12.268351	-6.919505	-2.617176
7	6	C	-9.539771	-0.587896	-0.821845
8	6	C	-6.951747	0.551813	-0.601281
9	6	C	-4.805998	-0.764781	-1.154946
10	6	C	-4.950439	-3.399068	-2.03017
11	8	O	-7.008867	2.939947	0.175124
12	6	C	-4.66438	4.078194	1.088858
13	6	C	-2.557588	3.392064	-0.826774
14	6	C	-2.254347	0.515869	-1.128321
15	6	C	0.029809	4.616576	-0.403039
16	6	C	1.556938	3.556029	1.800824
17	6	C	1.338033	0.702884	2.020971
18	6	C	-0.399471	-0.616555	0.733048
19	6	C	3.173677	-0.544347	3.795692
20	6	C	-5.214892	6.904859	1.088334
21	6	C	-4.320246	3.104765	3.791597
22	8	O	-3.043253	-4.576277	-2.61375
23	8	O	-11.421287	0.695095	-0.303328
24	8	O	-14.228781	-3.136996	-1.332224
25	8	O	-10.319924	-10.695538	-3.897759
26	1	H	-1.444826	0.159844	-3.024508
27	1	H	-3.278955	4.126529	-2.638261
28	8	O	4.096717	4.351076	1.387981
29	1	H	-5.935555	-8.130508	-3.403814
30	1	H	-14.111713	-7.823647	-2.77856
31	1	H	-0.116677	6.680594	-0.217324
32	1	H	1.177731	4.265164	-2.104278
33	1	H	0.882712	4.390199	3.602292
34	1	H	-0.466742	-2.671741	0.924411
35	1	H	3.027831	0.260051	5.718952
36	1	H	2.84164	-2.590264	3.928151
37	1	H	5.135827	-0.252187	3.154775
38	1	H	-5.48524	7.604166	-0.850406
39	1	H	-6.951649	7.279385	2.164862
40	1	H	-3.654864	7.957384	1.966162
41	1	H	-6.036077	3.542885	4.879425
42	1	H	-4.008082	1.056185	3.84402
43	1	H	-2.710813	4.024539	4.720151
44	1	H	-13.721382	-1.39812	-0.826441
45	1	H	-8.670027	-11.407988	-4.226757
46	1	H	5.041618	4.105275	2.927313

napG000008_en_		Standard Orientation (A.U.)			
Center number	Atomic number	Atomic Type	X	Y	Z
1	6	C	-12.196805	-4.465848	-1.785127
2	6	C	-9.791519	-3.276897	-1.5723
3	6	C	-7.586471	-4.696641	-2.072289
4	6	C	-7.741773	-7.215041	-2.764851
5	6	C	-10.134601	-8.379135	-2.969029
6	6	C	-12.339361	-7.019925	-2.485678
7	6	C	-9.639129	-0.638233	-0.831732
8	6	C	-7.05581	0.511048	-0.612059
9	6	C	-4.901564	-0.8216	-1.086096
10	6	C	-5.032269	-3.479825	-1.889521
11	8	O	-7.126583	2.925963	0.079841
12	6	C	-4.800179	4.096445	0.995165
13	6	C	-2.657994	3.34082	-0.853616
14	6	C	-2.349149	0.456297	-1.05028
15	6	C	-0.069896	4.562974	-0.41548
16	6	C	1.416287	3.567125	1.860362
17	6	C	1.18829	0.734404	2.167222
18	6	C	-0.53643	-0.612921	0.889597
19	6	C	2.97433	-0.448075	4.030895
20	6	C	-5.351566	6.920947	0.879042
21	6	C	-4.505906	3.225436	3.737814
22	8	O	-3.116787	-4.669639	-2.415693
23	8	O	-11.528326	0.656866	-0.373515
24	8	O	-14.319084	-3.205018	-1.332266
25	8	O	-10.371896	-10.828215	-3.63423
26	1	H	-1.503189	0.036923	-2.917332
27	1	H	-3.338245	4.014864	-2.704208
28	8	O	4.027145	4.167071	1.606621
29	1	H	-5.995347	-8.248019	-3.146633
30	1	H	-14.179734	-7.929986	-2.648183
31	1	H	-0.231035	6.636702	-0.309523
32	1	H	1.106824	4.145378	-2.081461
33	1	H	0.703048	4.444988	3.62659
34	1	H	-0.623723	-2.657752	1.161749
35	1	H	4.945947	-0.119197	3.455222
36	1	H	2.758112	0.419912	5.917226
37	1	H	2.646971	-2.492114	4.206157
38	1	H	-5.577814	7.550586	-1.089168
39	1	H	-7.112985	7.33131	1.901007
40	1	H	-3.815016	8.008044	1.756906
41	1	H	-6.242491	3.69815	4.777161
42	1	H	-4.189174	1.181258	3.871451
43	1	H	-2.91583	4.181774	4.66295
44	1	H	-13.819065	-1.453177	-0.865204
45	1	H	-8.717201	-11.54928	-3.917296
46	1	H	4.196186	5.982773	1.66987

Table S5 Binding free energies and energy components predicted by MM/GBSA (kcal/mol).

System	α-glucosidase/Acarbose	α-glucosidase/naphthgeranine G
ΔE_{vdW}	-15.61 ± 2.20	-23.74 ± 2.49
ΔE_{elec}	-48.25 ± 6.50	-20.54 ± 1.25
ΔG_{GB}	55.40 ± 4.43	31.65 ± 2.19
ΔG_{SA}	-3.35 ± 0.20	-3.93 ± 0.21
ΔG_{bind}	-11.83 ± 3.96	-16.57 ± 1.42

ΔE_{vdW}: van der Waals energy.
ΔE_{elec}: electrostatic energy.
ΔG_{GB}: electrostatic contribution to solvation.
ΔG_{SA}: non-polar contribution to solvation.
ΔG_{bind}: binding free energy.

Table S6 Docking output of compounds **5** – **12** and acarbose.

Ligands	Docking parametres	Mode								
		1	2	3	4	5	6	7	8	9
**5**	Energy (kcal/mol)	-7.2	-6.8	-6.8	-6.6	-6.5	-6.5	-6.4	-6.3	-6.2
	RMSD (1.b)	0.000	2.596	1.718	2.425	1.748	2.463	2.294	1.851	4.389
	RMSD (u.b)	0.000	6.072	6.309	6.706	3.663	4.746	6.571	6.598	6.656
**6**	Energy (kcal/mol)	-7.1	-6.9	-6.8	-6.6	-6.4	-6.4	-6.4	-6.3	-6.2
	RMSD (1.b)	0.000	2.640	1.811	1.634	1.789	2.493	1.920	2.276	1.619
	RMSD (u.b)	0.000	6.132	3.750	6.278	2.403	6.566	6.487	3.458	3.680
**7**	Energy (kcal/mol)	-7.7	-6.8	-6.8	-6.7	-6.6	-6.5	-6.4	-6.3	-6.3
	RMSD (1.b)	0.000	1.581	1.595	2.063	2.241	2.302	1.838	2.109	2.390
	RMSD (u.b)	0.000	3.408	2.083	6.426	6.780	6.179	2.340	6.389	6.396
**8**	Energy (kcal/mol)	-6.4	-6	-6	-6	-5.9	-5.8	-5.8	-5.7	-5.7
	RMSD (1.b)	0.000	3.897	2.320	3.201	1.765	3.617	2.745	3.089	3.217
	RMSD (u.b)	0.000	6.656	4.227	4.400	4.729	6.172	5.408	4.063	5.083
**9**	Energy (kcal/mol)	-7.3	-6.6	-5.9	-5.8	-5.8	-5.8	-5.7	-5.6	-5.6
	RMSD (1.b)	0.000	1.659	2.074	4.304	4.118	1.573	4.359	4.454	3.677
	RMSD (u.b)	0.000	2.943	4.672	6.512	6.561	2.057	6.545	5.570	5.060
**10**	Energy (kcal/mol)	-7.0	-6.2	-5.7	-5.6	-5.5	-5.5	-5.5	-5.4	-5.3
	RMSD (1.b)	0.000	2.159	3.260	4.259	2.838	4.104	4.906	1.863	3.560
	RMSD (u.b)	0.000	3.149	4.408	6.323	5.364	6.586	6.006	2.447	4.937
**11**	Energy (kcal/mol)	-6.2	-6.0	-5.5	-5.5	-5.4	-5.4	-5.3	-5.3	-5.2
	RMSD (1.b)	0.000	2.325	3.269	2.447	3.455	3.815	3.641	4.006	4.071
	RMSD (u.b)	0.000	5.050	5.320	4.020	6.276	5.631	5.557	6.235	5.716
**12**	Energy (kcal/mol)	-6.3	-6.3	-6.3	-6.2	-5.1	-5.0	-5.0	-4.9	-4.9
	RMSD (1.b)	0.000	0.122	0.126	0.095	1.276	1.466	1.480	4.157	4.139
	RMSD (u.b)	0.000	2.139	5.230	4.775	5.463	2.279	3.101	6.915	6.059
Acarbose	Energy (kcal/mol)	-6.7	-6.5	-6.4	-6.3	-6.2	-6.1	-6.1	-6.0	-6.0
	RMSD (1.b)	0.000	3.787	4.511	1.761	2.360	5.077	2.788	3.348	4.258
	RMSD (u.b)	0.000	5.427	9.513	2.588	10.099	8.126	5.536	8.691	5.733

Table S7 Antibacterial activity (MIC, μg/mL) of compounds **5 – 12**.

Bacteria	MIC value (μg/mL)								
	LEV	**5**	**6**	**7**	**8**	**9**	**10**	**11**	**12**
Staphylococcus aureus ATCC 29213	0.25	>64	>64	>64	>64	64	32	NT	NT
MRSA	0.5	>64	>64	>64	>64	16	>64	>64	16
Klebsiella pneumoniae ATCC 13883	1	>64	>64	>64	>64	>64	>64	>64	>64
Pseudomonas aeruginosa ATCC 9027	2	>64	>64	>64	>64	>64	>64	NT	NT

Abbreviation: NT, not tested; LEV, levofloxacin.

Figure S1 HPLC analysis of the culture broths from the *Streptomyces* sp. PH9001 – PH9030

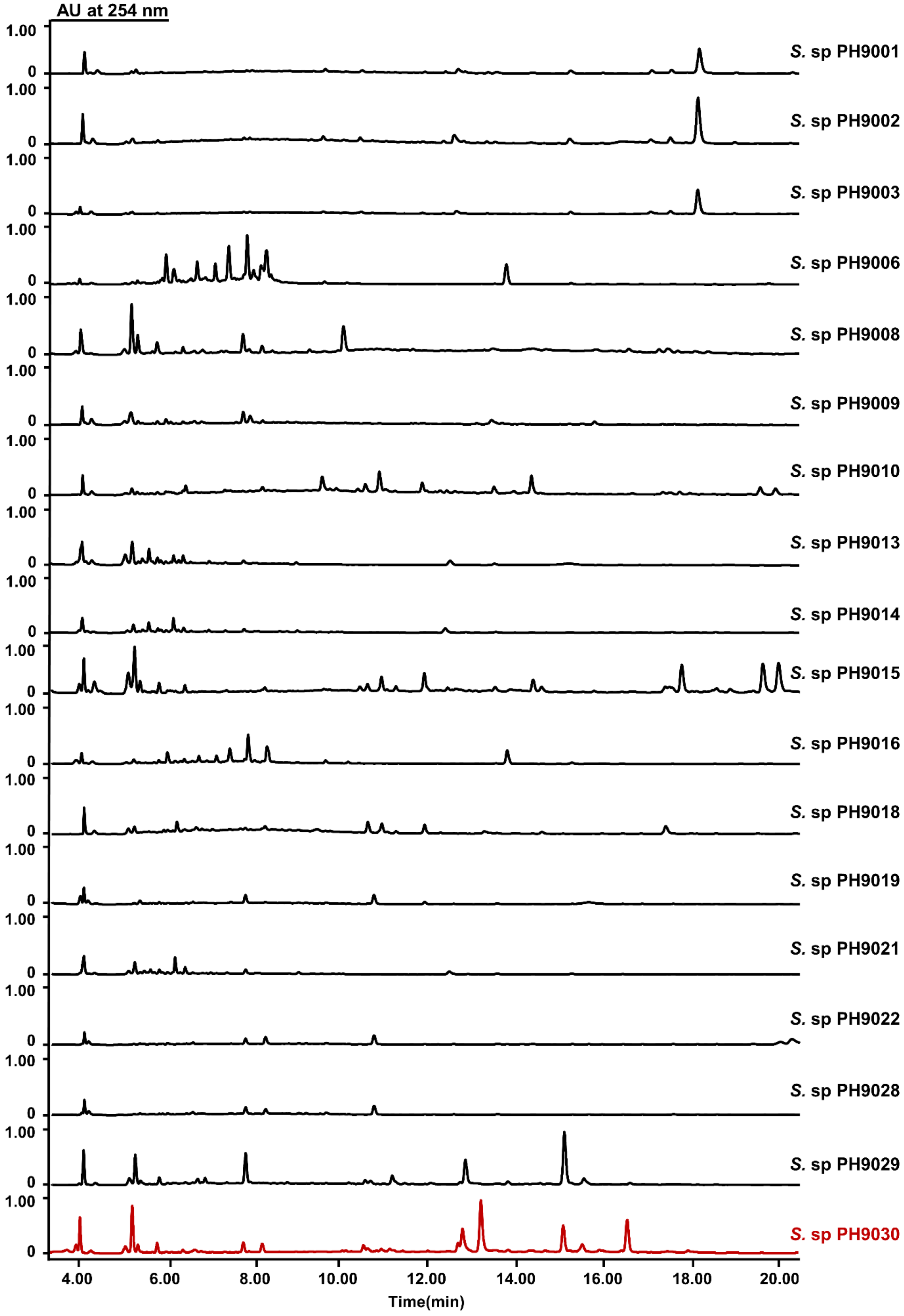

Figure S2 The Antibacterial activity of *Streptomyces* sp. PH9001 – PH9030

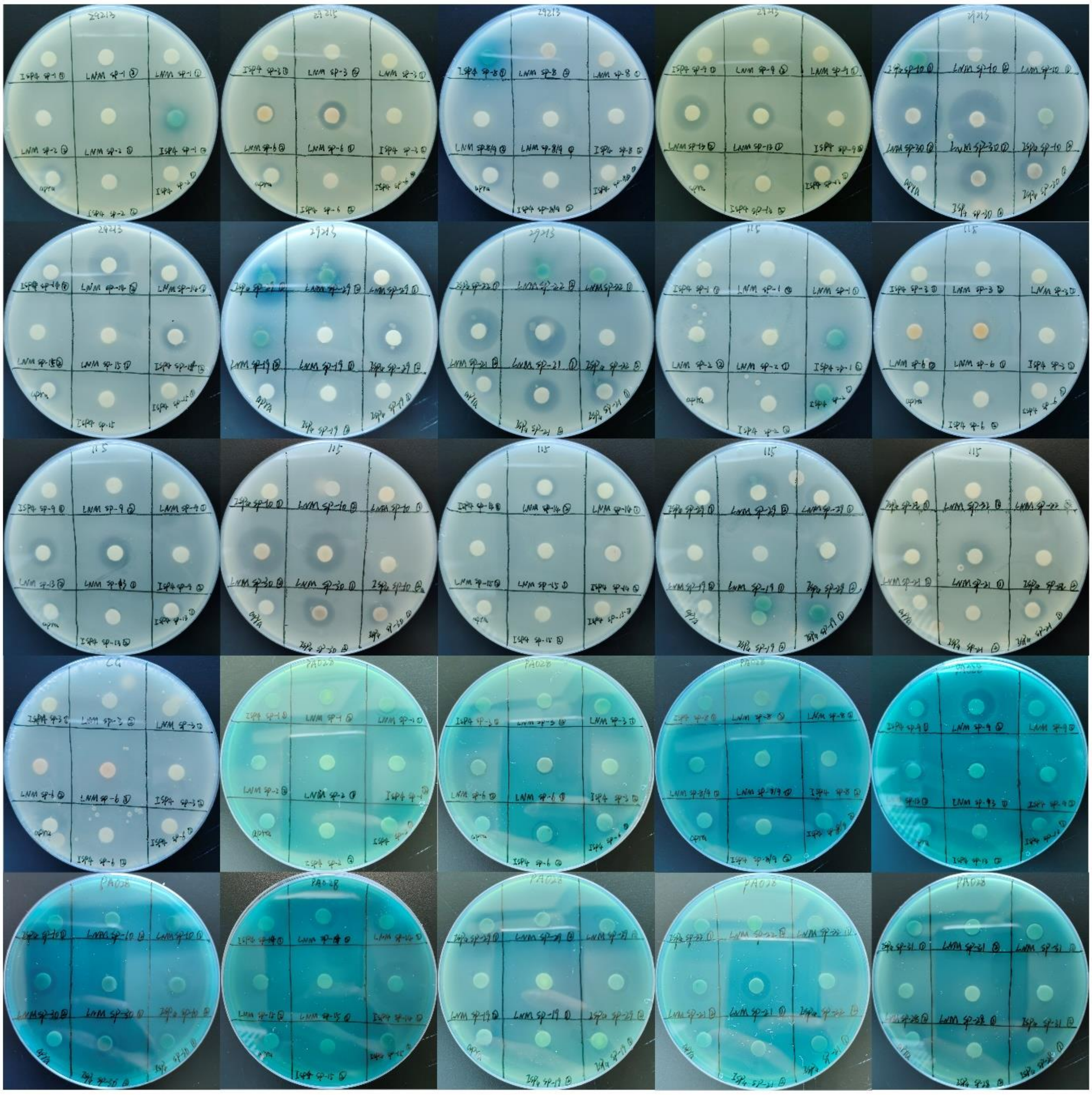

Figure S3 The partial 16S rRNA gene sequences data of *Streptomyces* sp. PH9030.

TGCAGTCGAACGATGAAGCCCTTCGGGGTGGATTAGTGGCGAACGGGTGAGTAACACGTGGGCAATC
TGCCCTTCACTCTGGGACAAGCCCTGGAAACGGGGTCTAATACCGGATATGAGCCTGGGAGGCATCTC
CCGGGTTGTAAAGCTCCGGCGGTGAAGGATGAGCCCGCGGCCTATCAGCTTGTTGGTGAGGTAATGG
CTCACCAAGGCGACGACGGGTAGCCGGCCTGAGAGGGCGACCGGCCACACTGGGACTGAGACACGG
CCCAGACTCCTACGGGAGGCAGCAGTGGGGAATATTGCACAATGGGCGAAAGCCTGATGCAGCGACG
CCGCGTGAGGGATGACGGCCTTCGGGTTGTAAACCTCTTTCAGCAGGGAAGAAGCGAAAGTGACGG
TACCTGCAGAAGAAGCGCCGGCTAACTACGTGCCAGCAGCCGCGGTAATACGTAGGGCGCAAGCGTT
GTCCGGAATTATTGGGCGTAAAGAGCTCGTAGGCGGCTTGTCACGTCGATTGTGAAAGCCCGAGGCTT
AACCTCGGGTCTGCAGTCGATACGGGCTAGCTAGAGTGTGGTAGGGGAGATCGGAATTCCTGGTGTA
GCGGTGAAATGCGCAGATATCAGGAGGAACACCGGTGGCGAAGGCGGATCTCTGGGCCATTACTGAC
GCTGAGGAGCGAAAGCGTGGGGAGCGAACAGGATTAGATACCCTGGTAGTCCACGCCGTAAACGGT
GGGAACTAGGTGTTGGCGACATTCCACGTCGTCGGTGCCGCAGCTAACGCATTAAGTTCCCCGCCTGG
GGAGTACGGCCGCAAGGCTAAAACTCAAAGGAATTGACGGGGGCCCGCACAAGCGGCGGAGCATGT
GGCTTAATTCGACGCAACGCGAAGAACCTTACCAAGGCTTGACATACACCGGAAAGCATTAGAGATA
GTGCCCCCCTTGTGGTCGGTGTACAGGTGGTGCATGGCTGTCGTCAGCTCGTGTCGTGAGATGTTGGG
TTAAGTCCCGCAACGAGCGCAACCCTTGTTCTGTGTTGCCAGCATGCCCTTCGGGGTGATGGGGACTC
ACAGGAGACCGCCGGGGTCAACTCGGAGGAAGGTGGGGACGACGTCAAGTCATCATGCCCCTTATGT
CTTGGGCTGCACACGTGCTACAATGGCCGGTACAATGAGCTGCGATACCGTGAGGTGGAGCGAATCT
CAAAAAGCCGGTCTCAGTTCGGATTGGGGTCTGCAACTCGACCCCATGAAGTCGGAGTCGCTAGTAA
TCGCAGATCAGCATTGCTGCGGTGAATACGTTCCCGGGCCTTGTACACACCGCCCGTCACGTCACGAA
AGTCGGTAACACCCGAAGCCGGTGGCCCAACCCCTTGTGGGAGGGAGCTGTCGAAGGTGGGACTGG
CGATTGGGACGAAGTCGTAACAAGGTAGCCGTACCGGAAGGTGCGGCTGGATCACCTCCT

Figure S4 ^{1}H NMR spectrum of **5** in DMSO-d_6 (600 MHz)

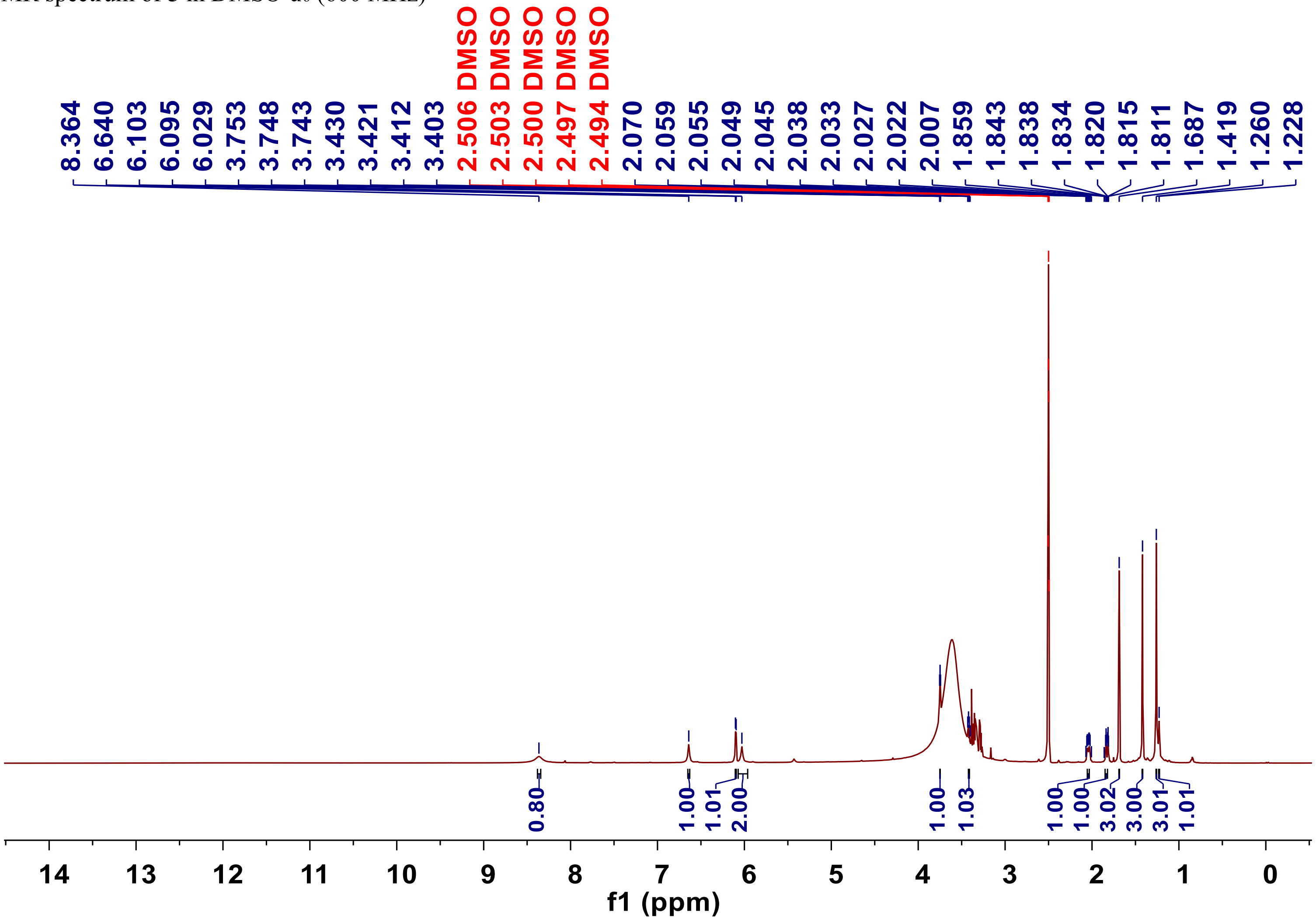

Figure S5 ^{13}C NMR spectrum of **5** in DMSO-d_6 (150 MHz)

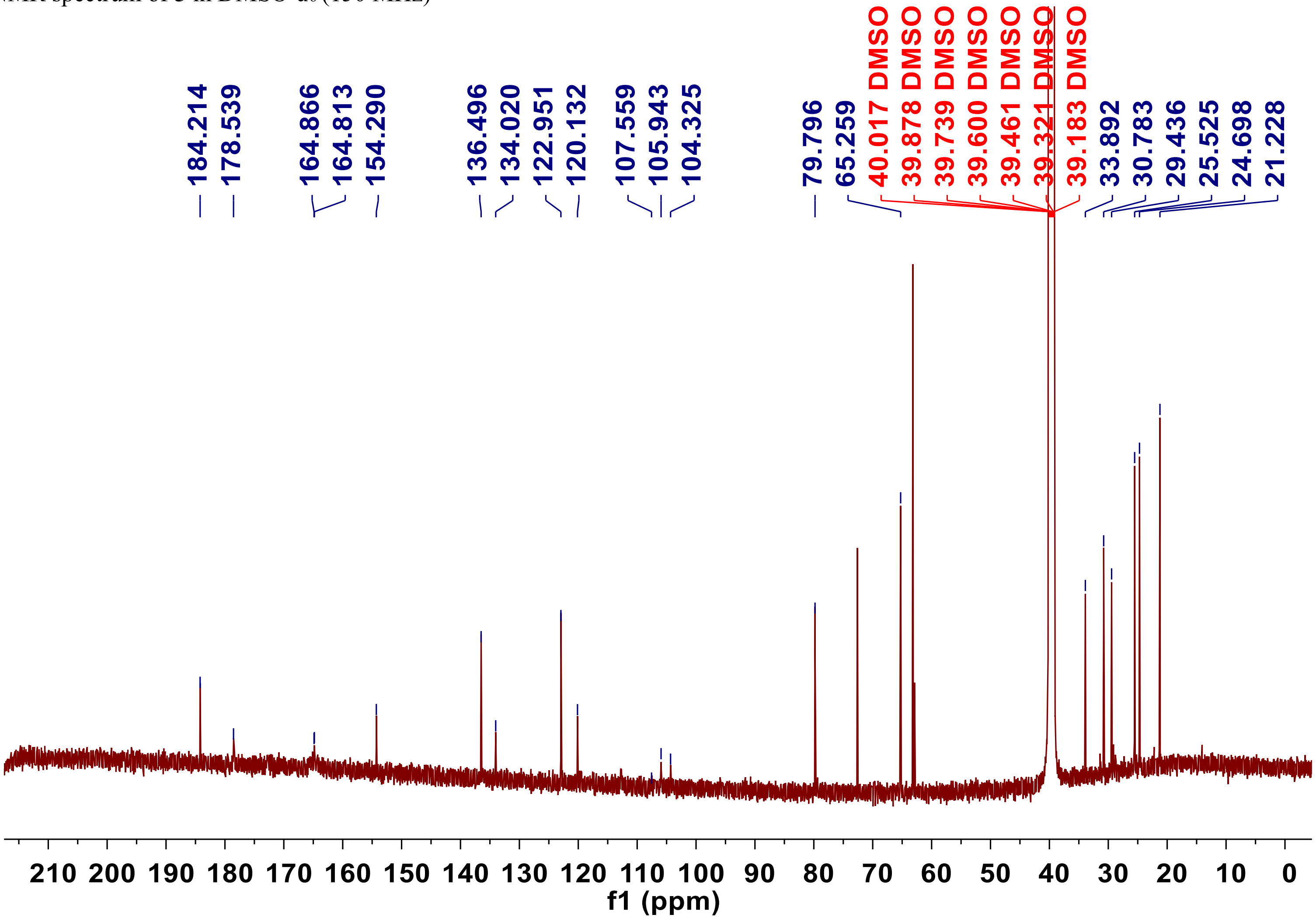

Figure S6 DEPT-90 spectrum of **5**

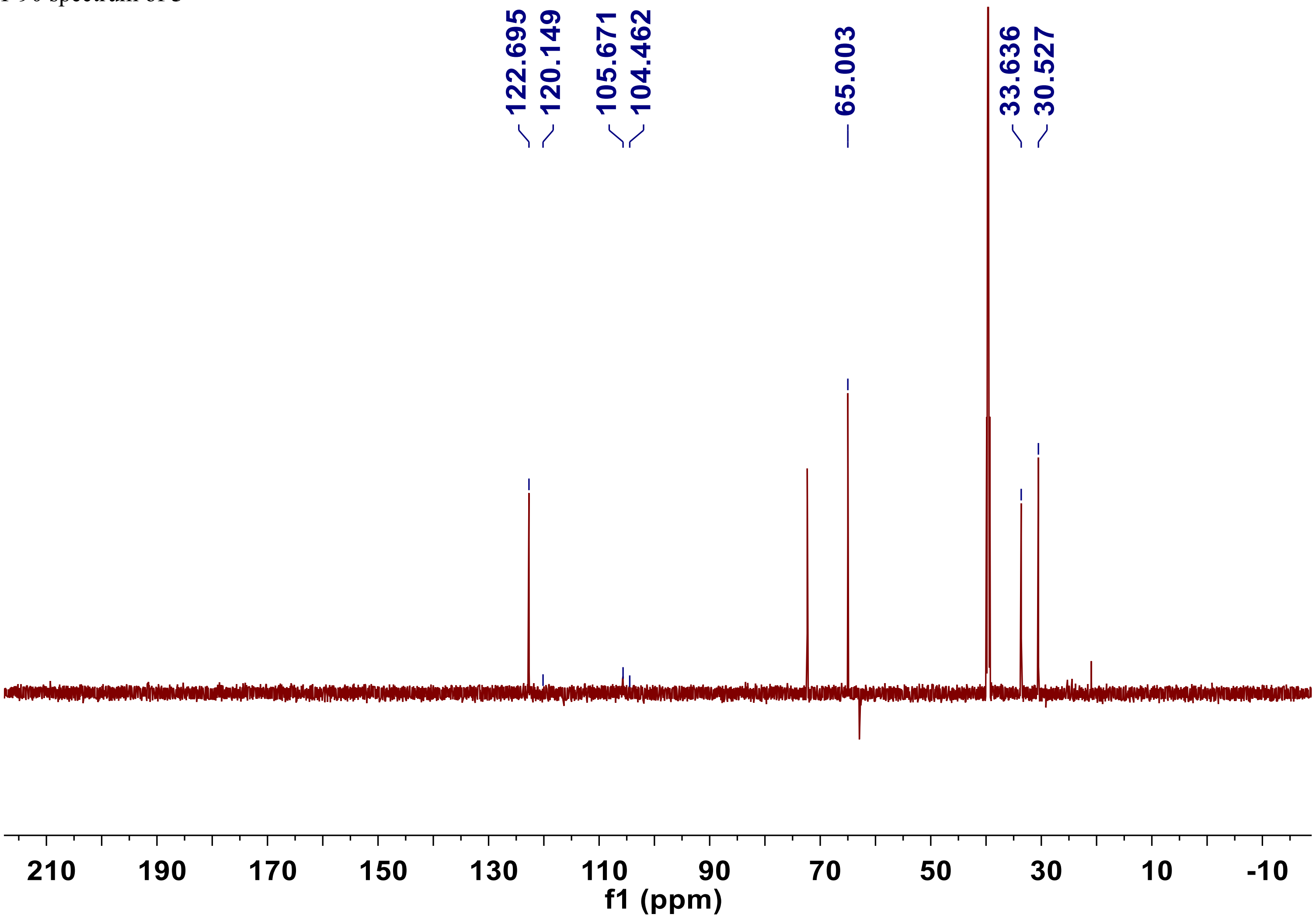

Figure S7 DEPT-135 spectrum of **5**

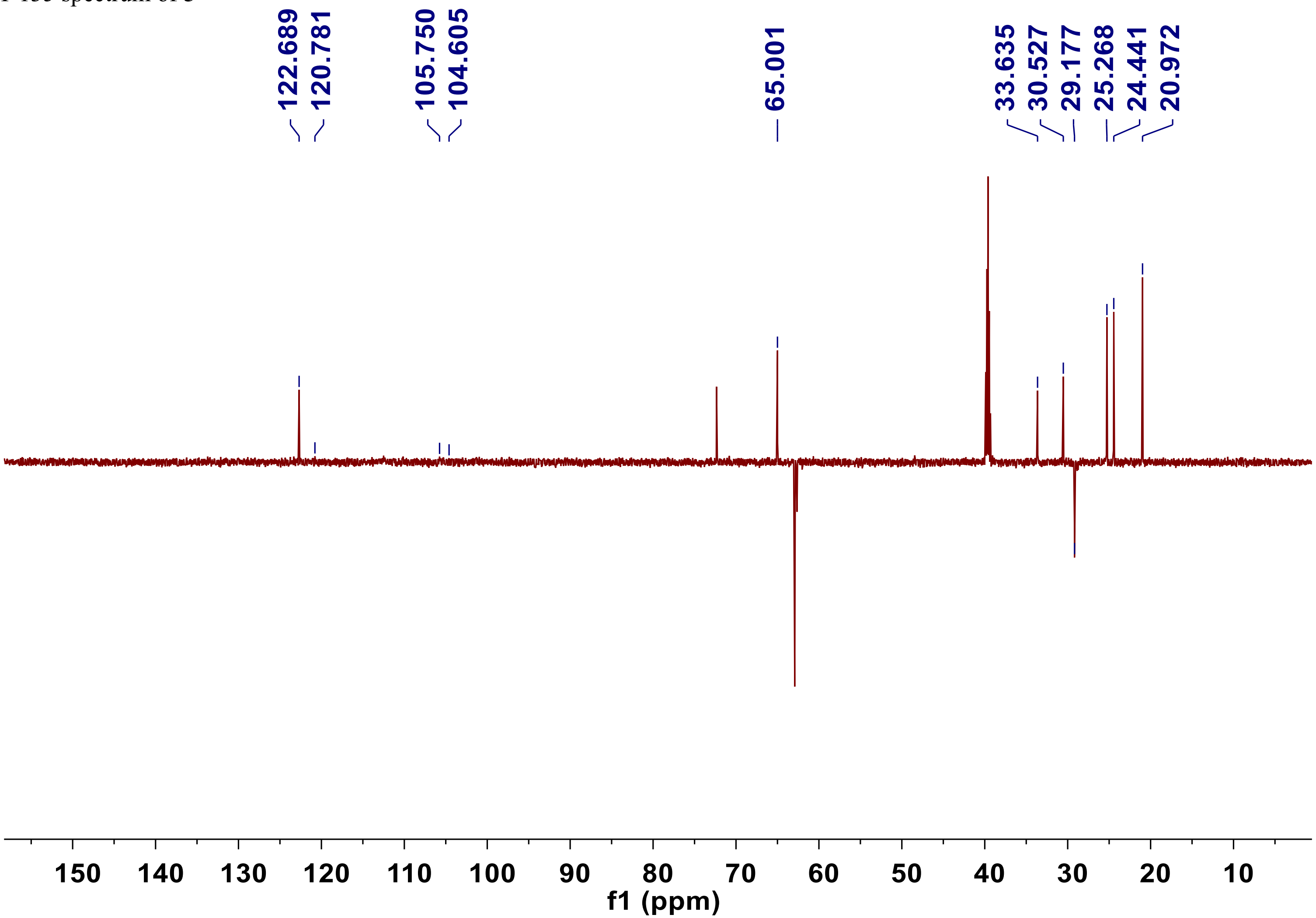

Figure S8 HSQC spectrum of **5**

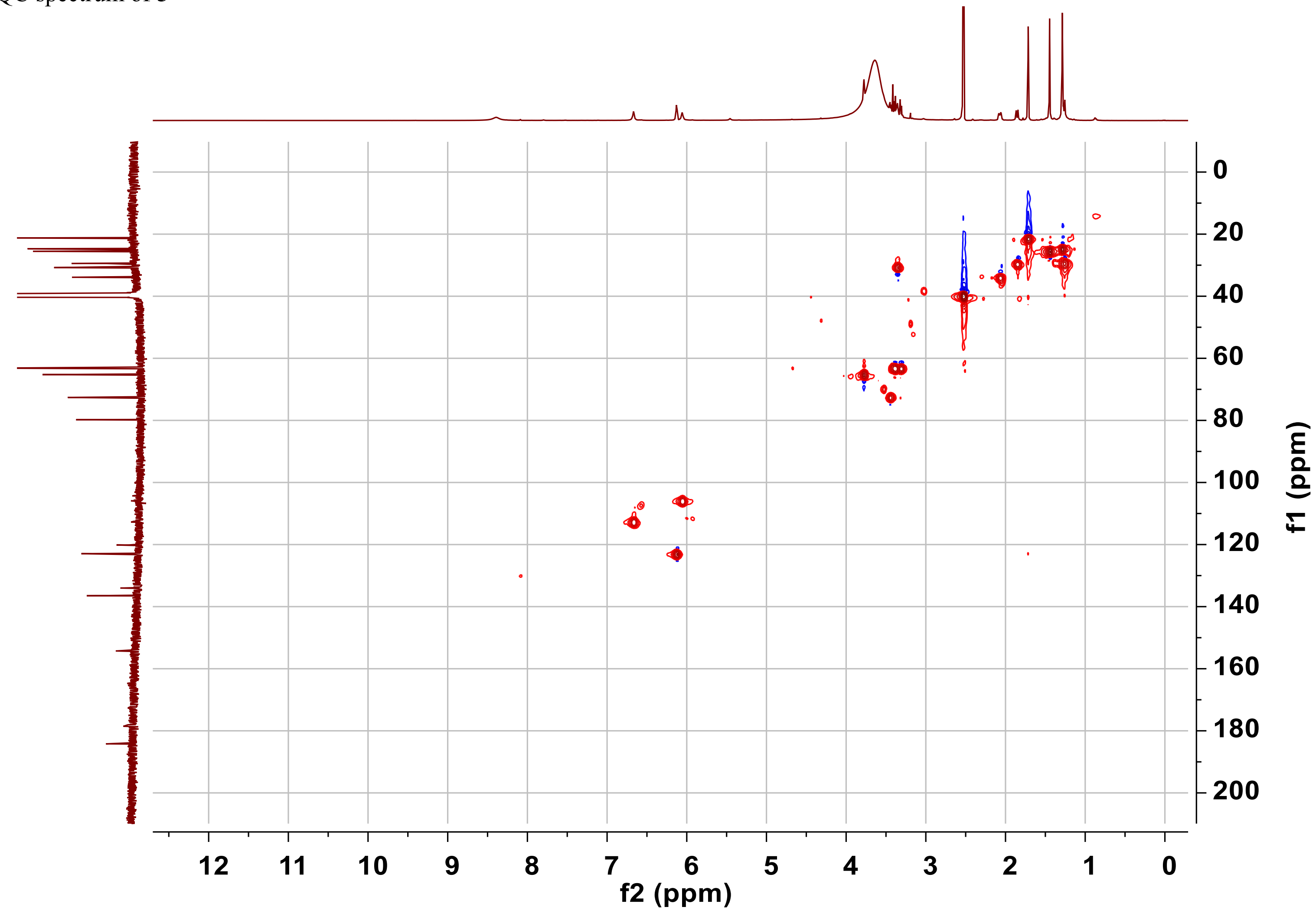

Figure S9 HMBC spectrum of **5**

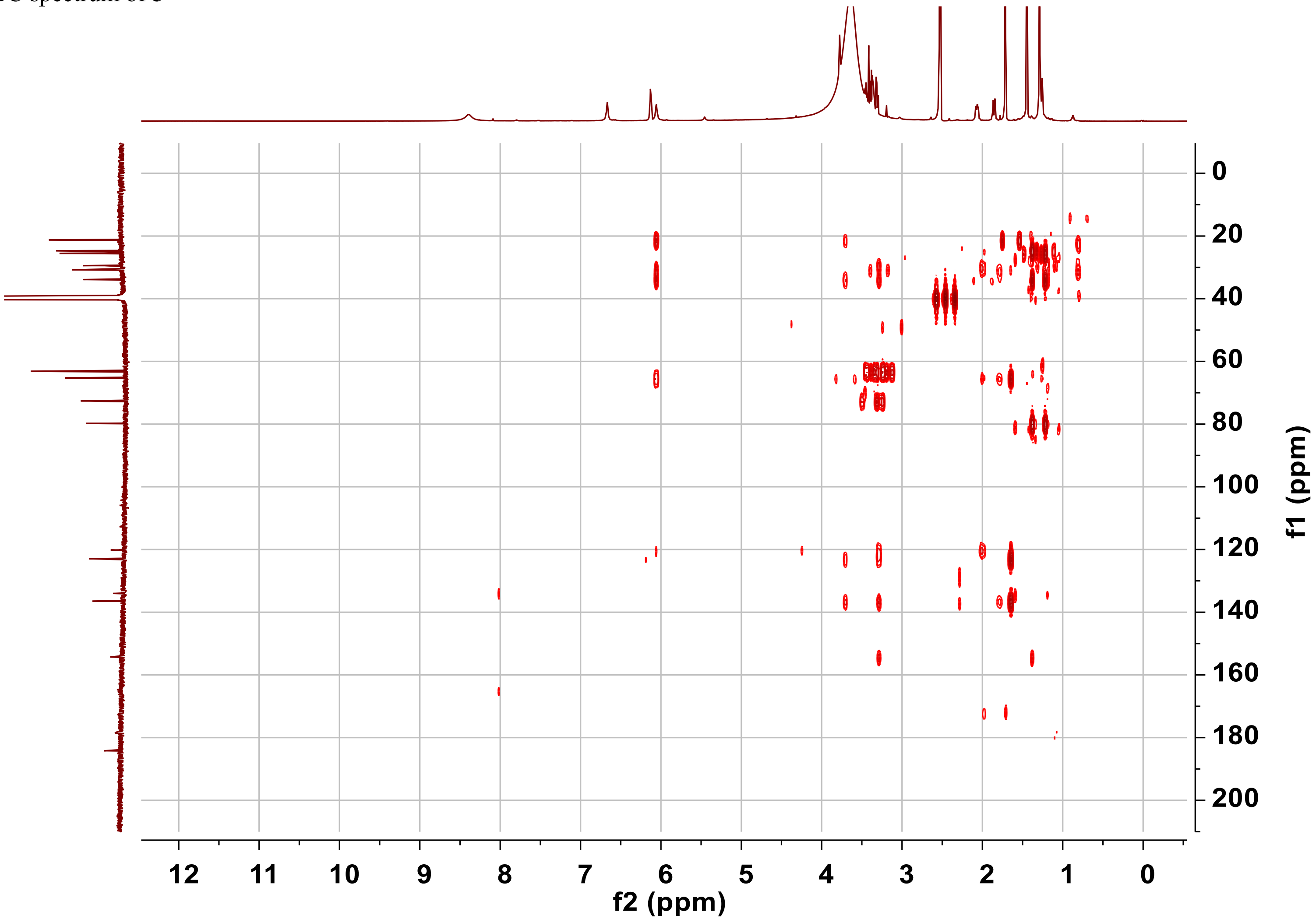

Figure S10 ^{1}H-^{1}H COSY spectrum of **5**

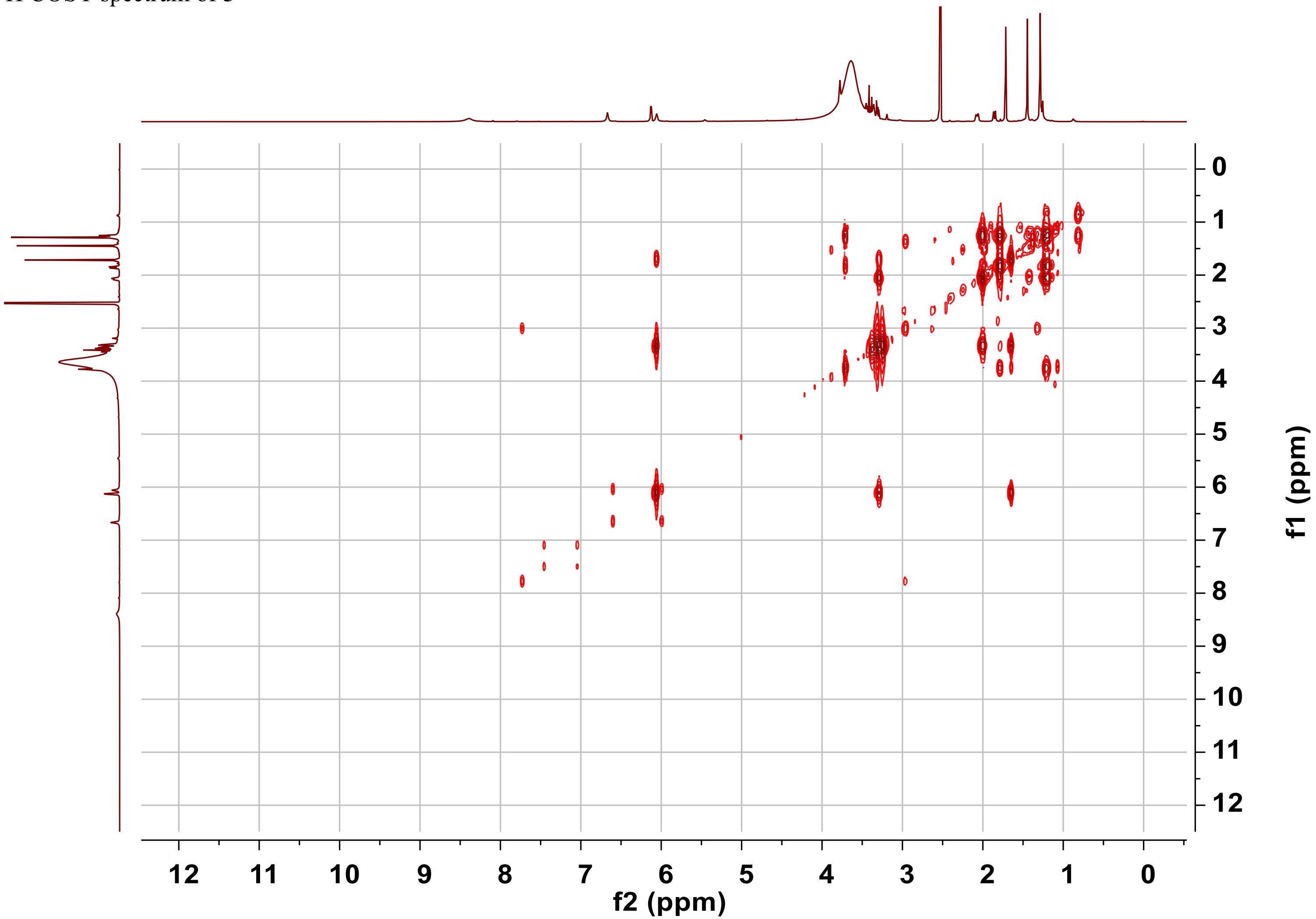

Figure S11 NOESY spectrum of **5**

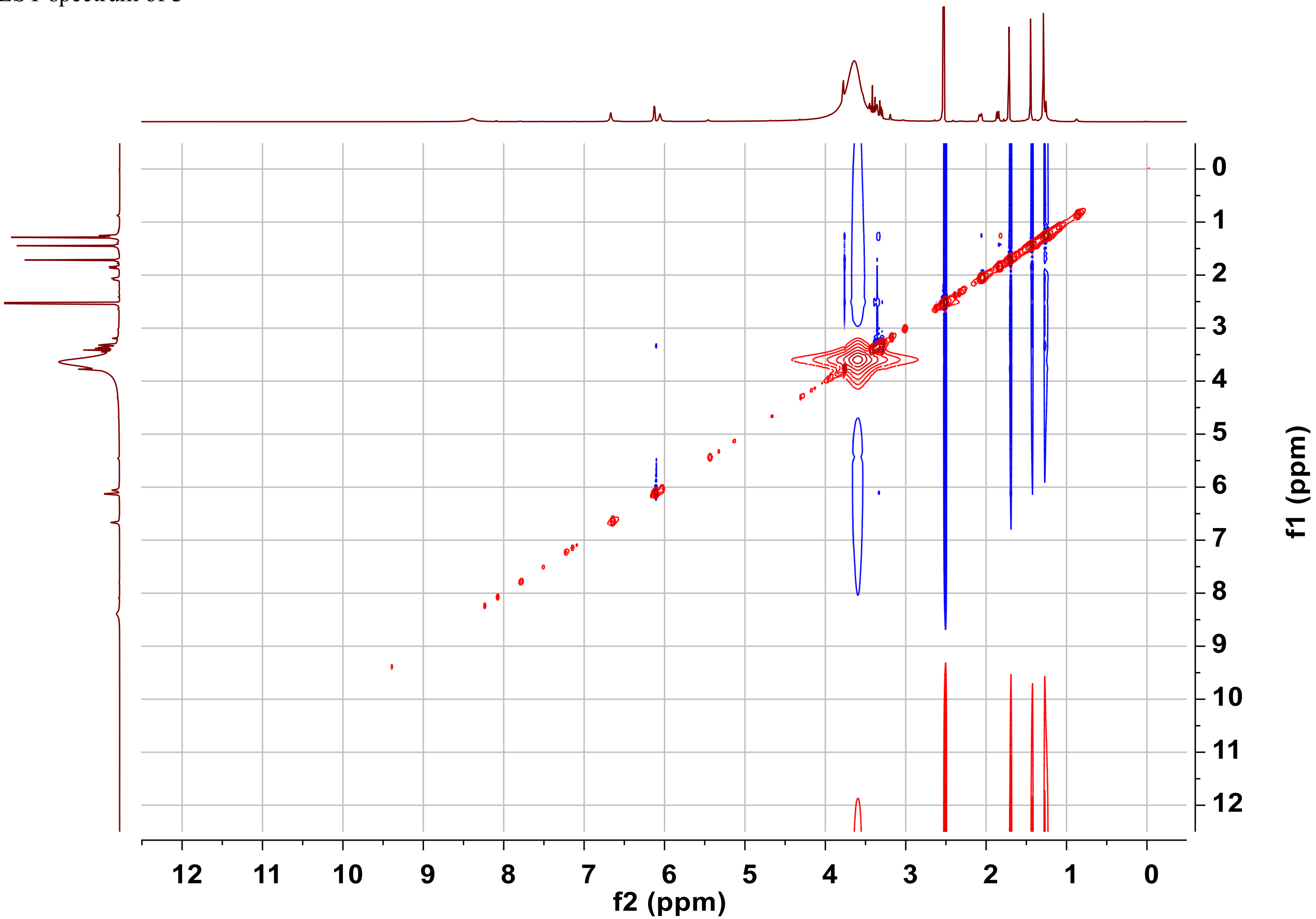

Figure S12 HRESIMS spectrum of **5**

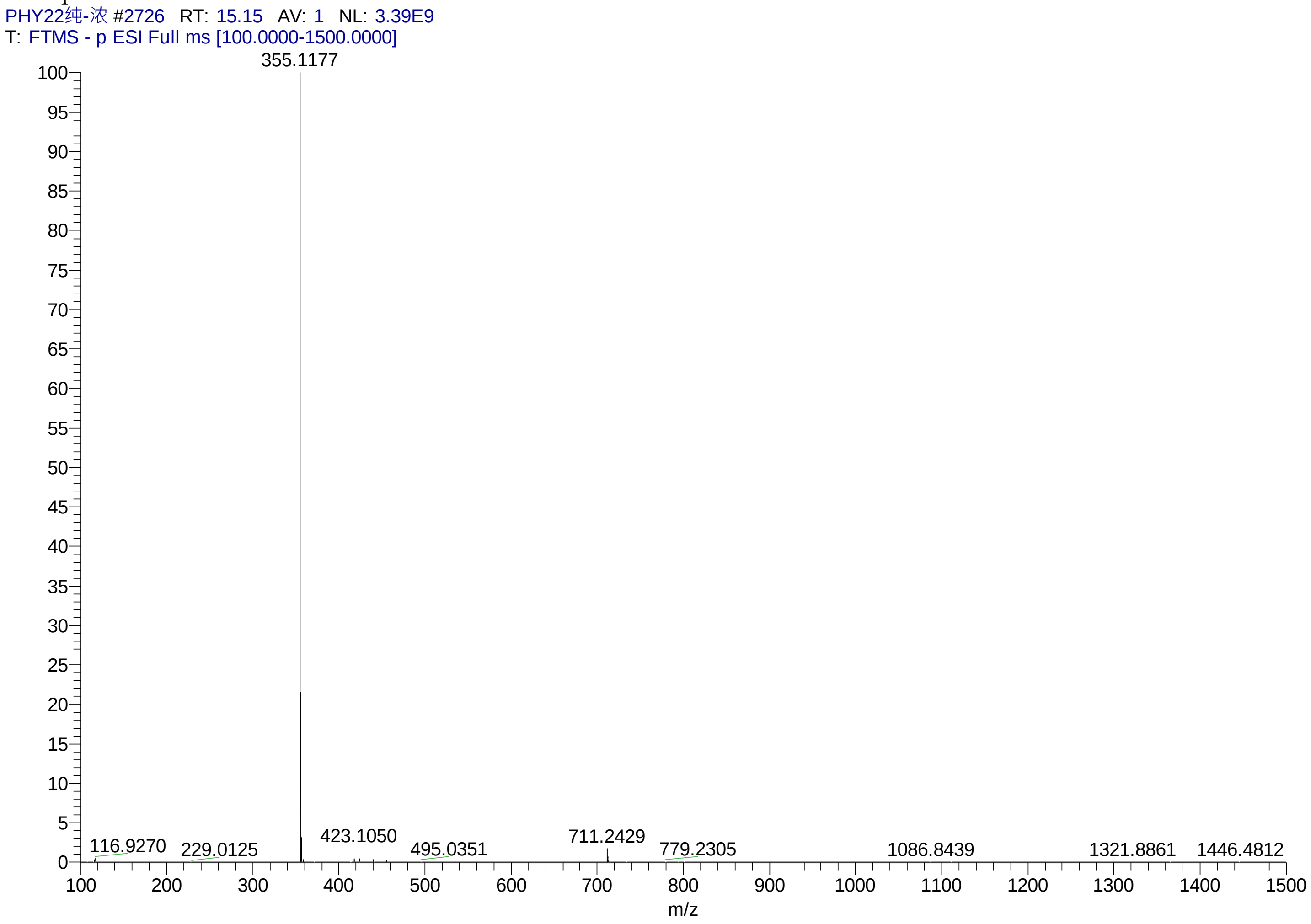

Figure S13 UV spectrum of **5**

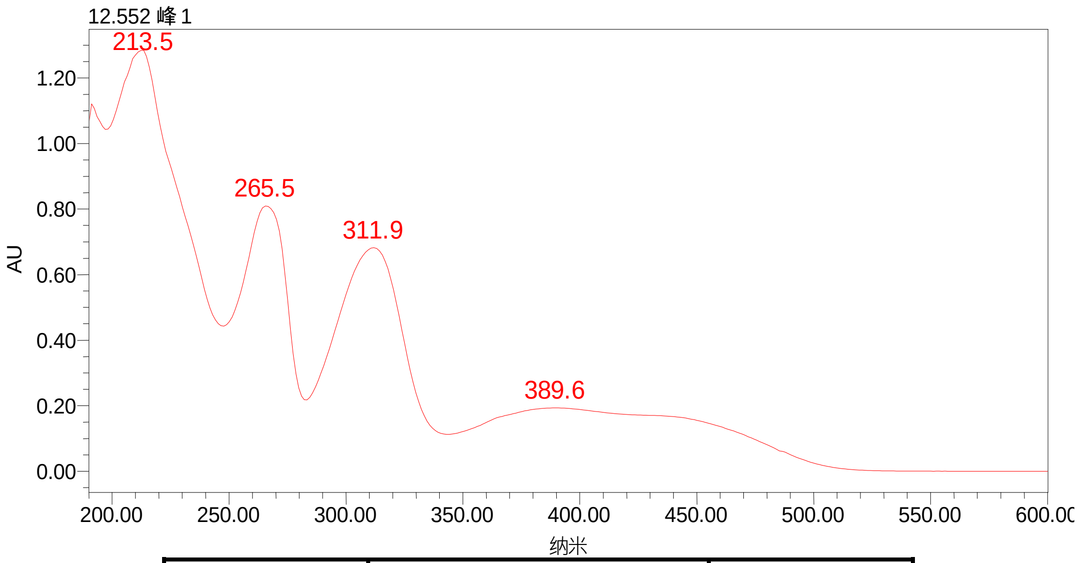

No.	Wavelength (nm)	Abs
1	213.5	1.285
2	265.5	0.809
3	312.0	0.682
4	389.6	0.193

Figure S14 Optical rotation spectrum of **5**

Rudolph Research Analytical

This sample was measured on an Autopol VI, Serial #91058
Manufactured by Rudolph Research Analytical, Hackettstown, NJ, USA.

Measurement Date : Wednesday, 31-JAN-2024

Set Temperature : 20.0

Time Delay : Disabled

Delay between Measurement : Disabled

n	Average	Std.Dev.	% RSD	Maximum	Minimum
5	-276.67	9.13	-3.29	-266.67	-283.33

S.No	Sample ID	Time	Result	Scale	OR °Arc	WLG.nm	Lg.mm	Conc.g/100ml	Temp.
1	PHY22	01:57:03 PM	-266.67	SR	-0.016	589	100.00	0.006	20.0
2	PHY22	01:57:10 PM	-283.33	SR	-0.017	589	100.00	0.006	20.0
3	PHY22	01:57:16 PM	-283.33	SR	-0.017	589	100.00	0.006	20.0
4	PHY22	01:57:22 PM	-283.33	SR	-0.017	589	100.00	0.006	20.0
5	PHY22	01:57:29 PM	-266.67	SR	-0.016	589	100.00	0.006	20.0

Figure S15 ^{1}H NMR spectrum of **6** in DMSO-d_6 (600 MHz)

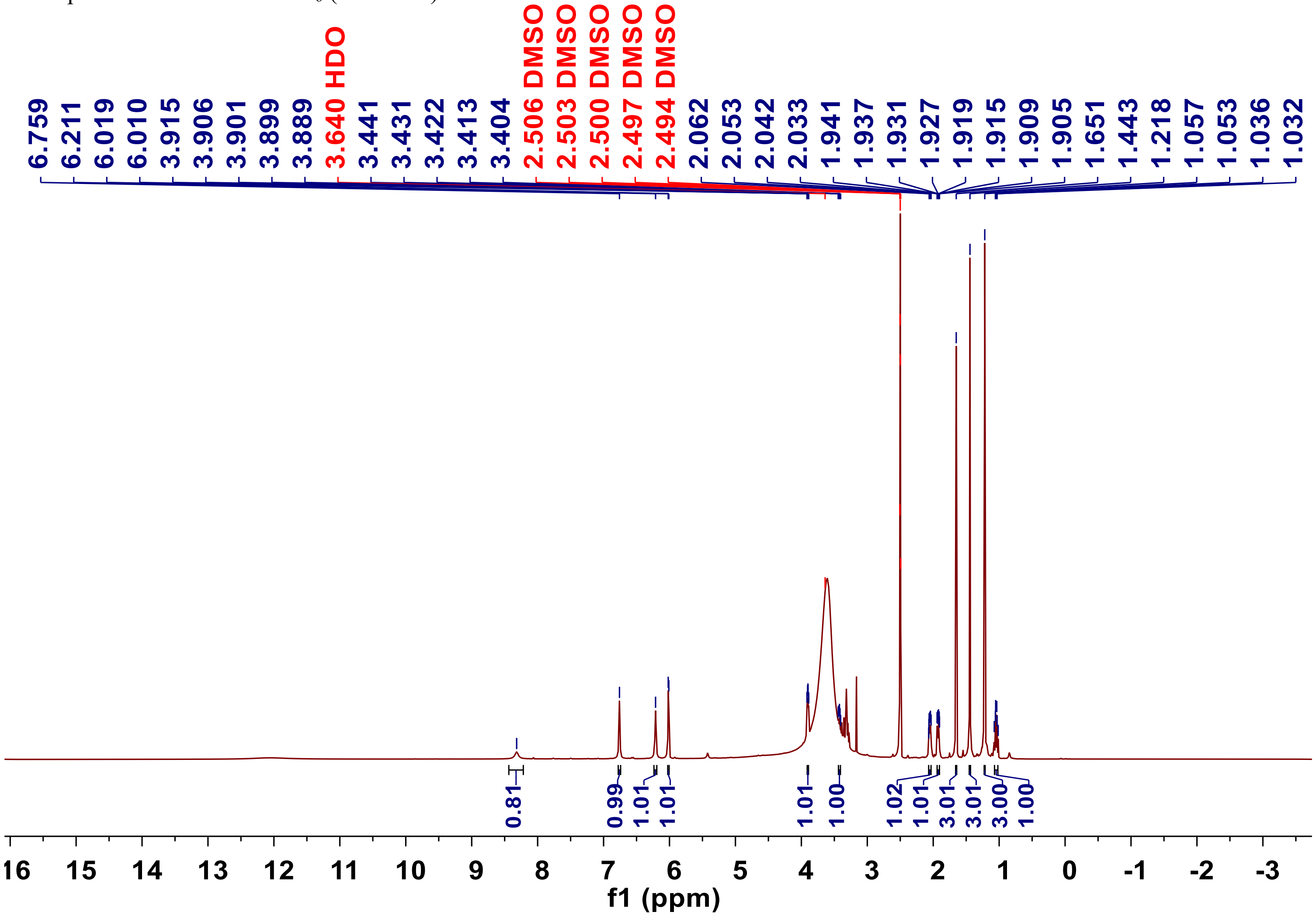

Figure S16 ^{13}C NMR spectrum of **6** in DMSO-d_6 (150 MHz)

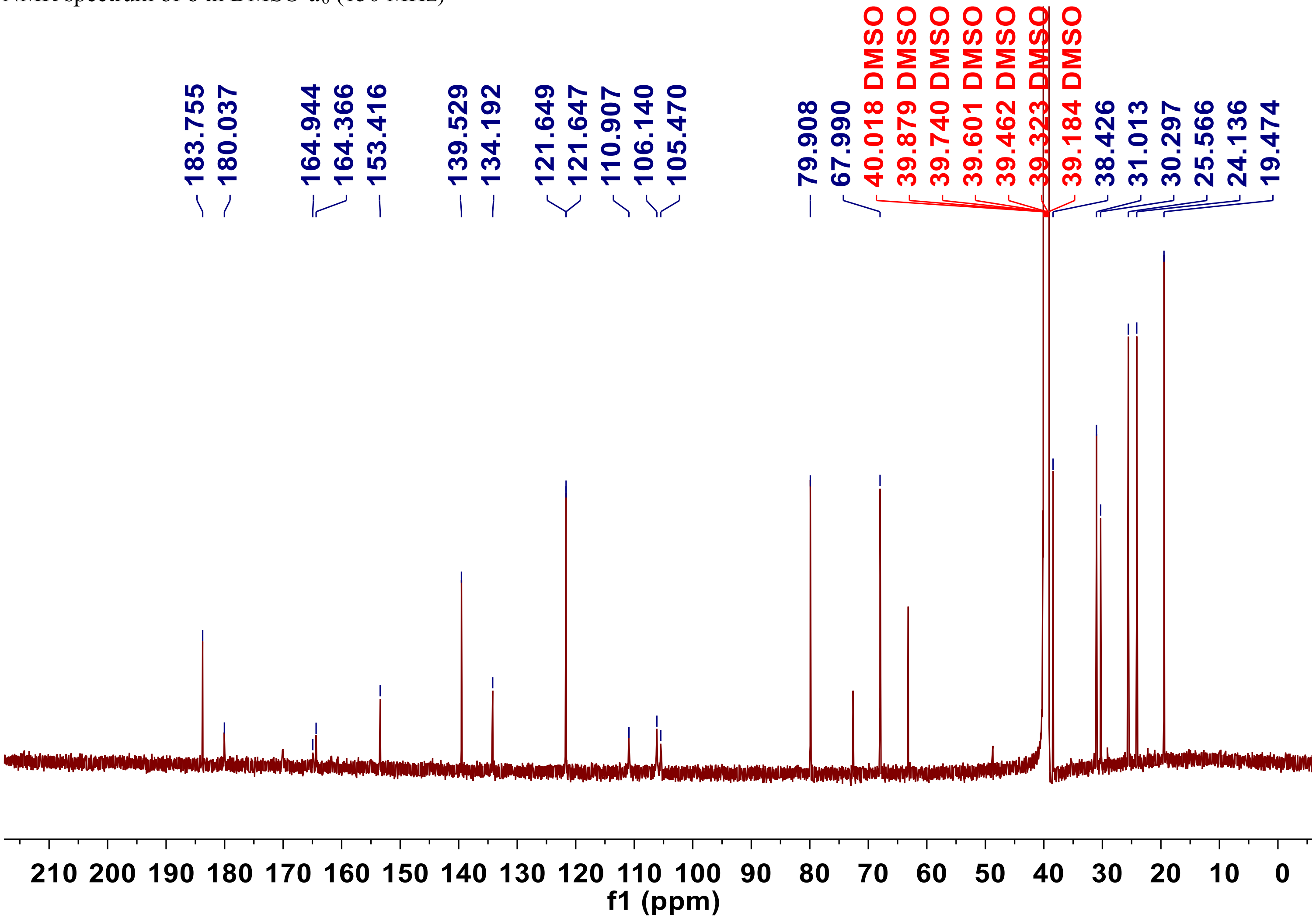

Figure S17 DEPT-90 spectrum of **6**

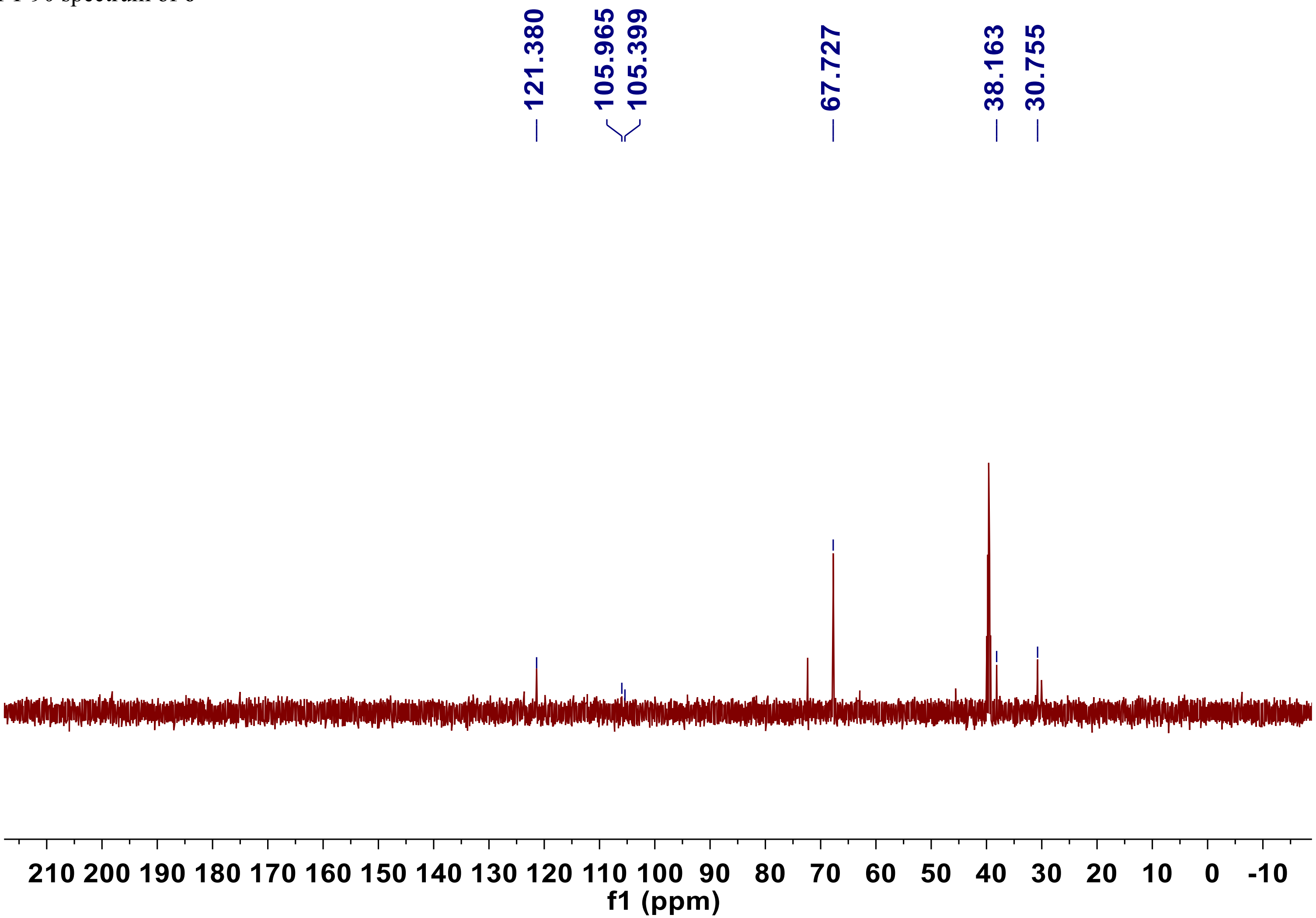

Figure S18 DEPT-135 spectrum of **6**

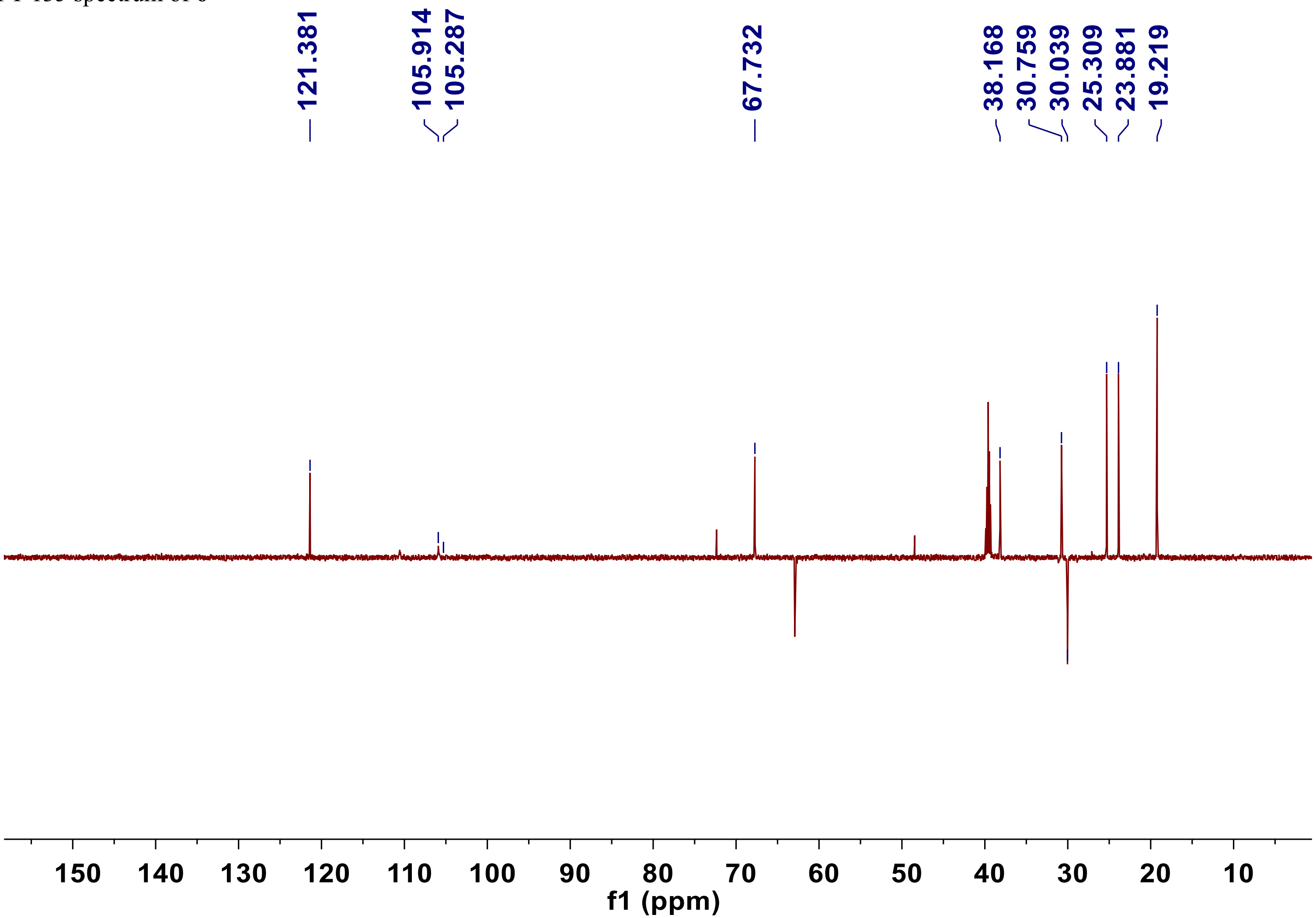

Figure S19 HSQC spectrum of **6**

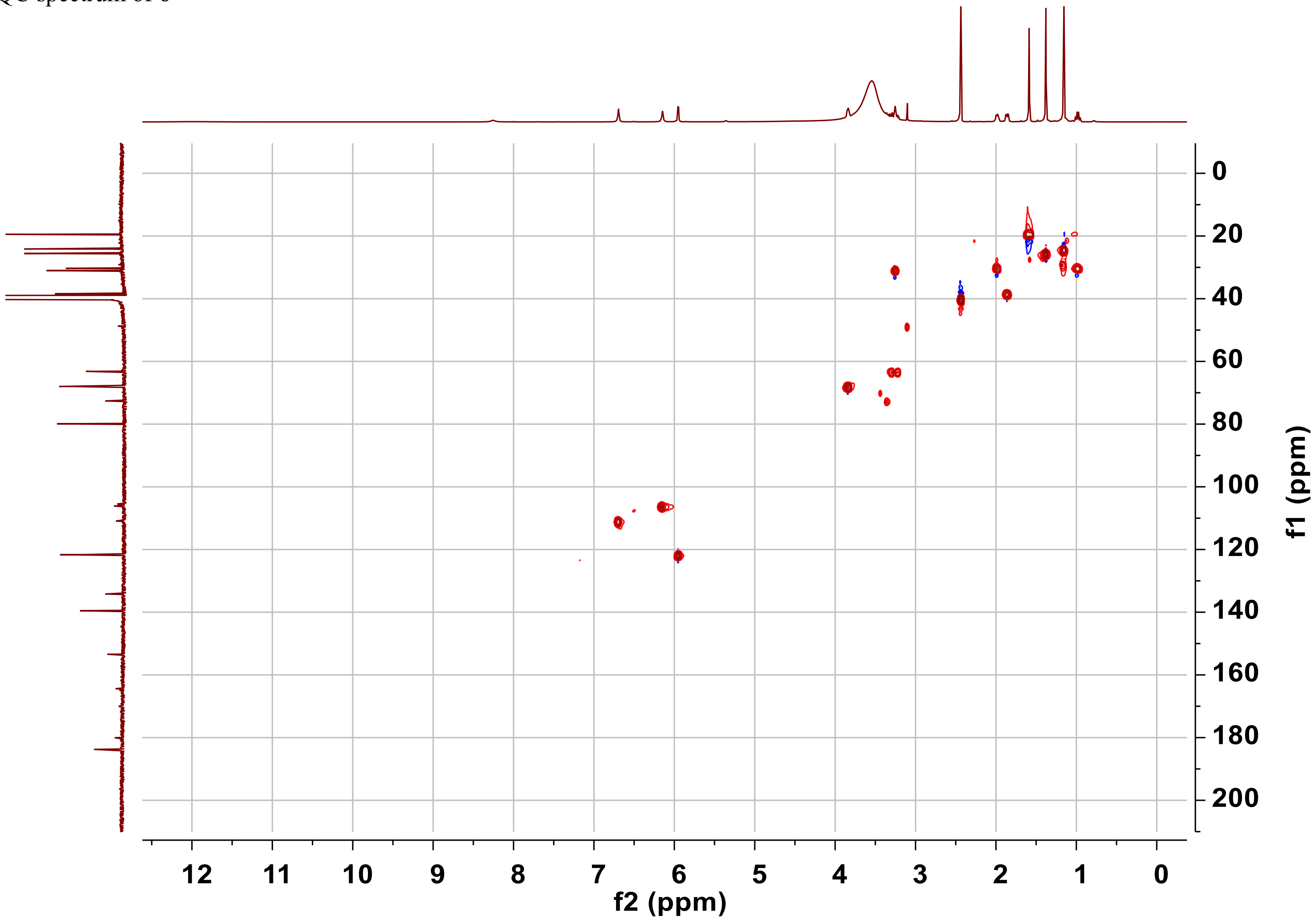

Figure S20 HMBC spectrum of **6**

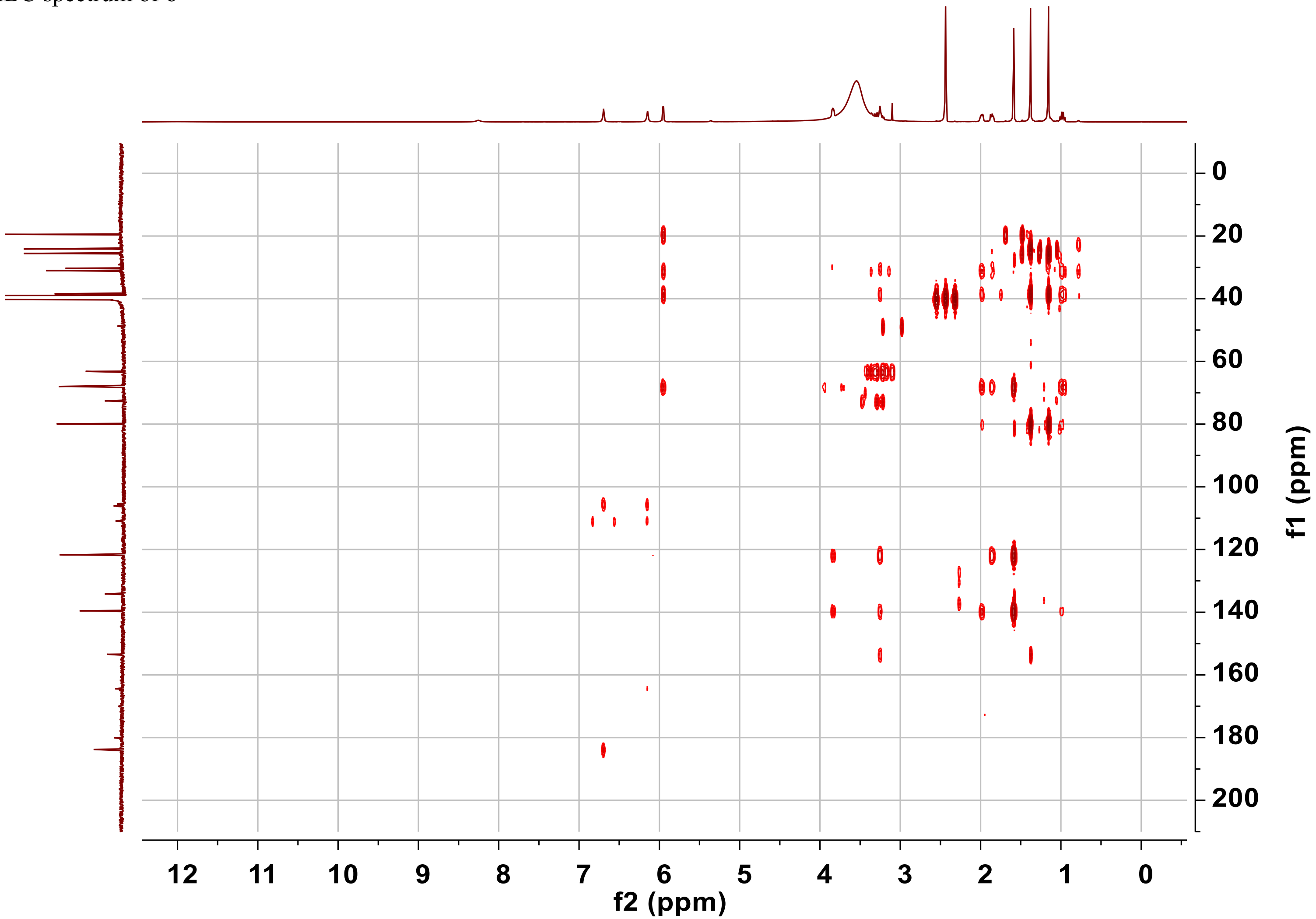

Figure S21 ^{1}H-^{1}H COSY spectrum of **6**

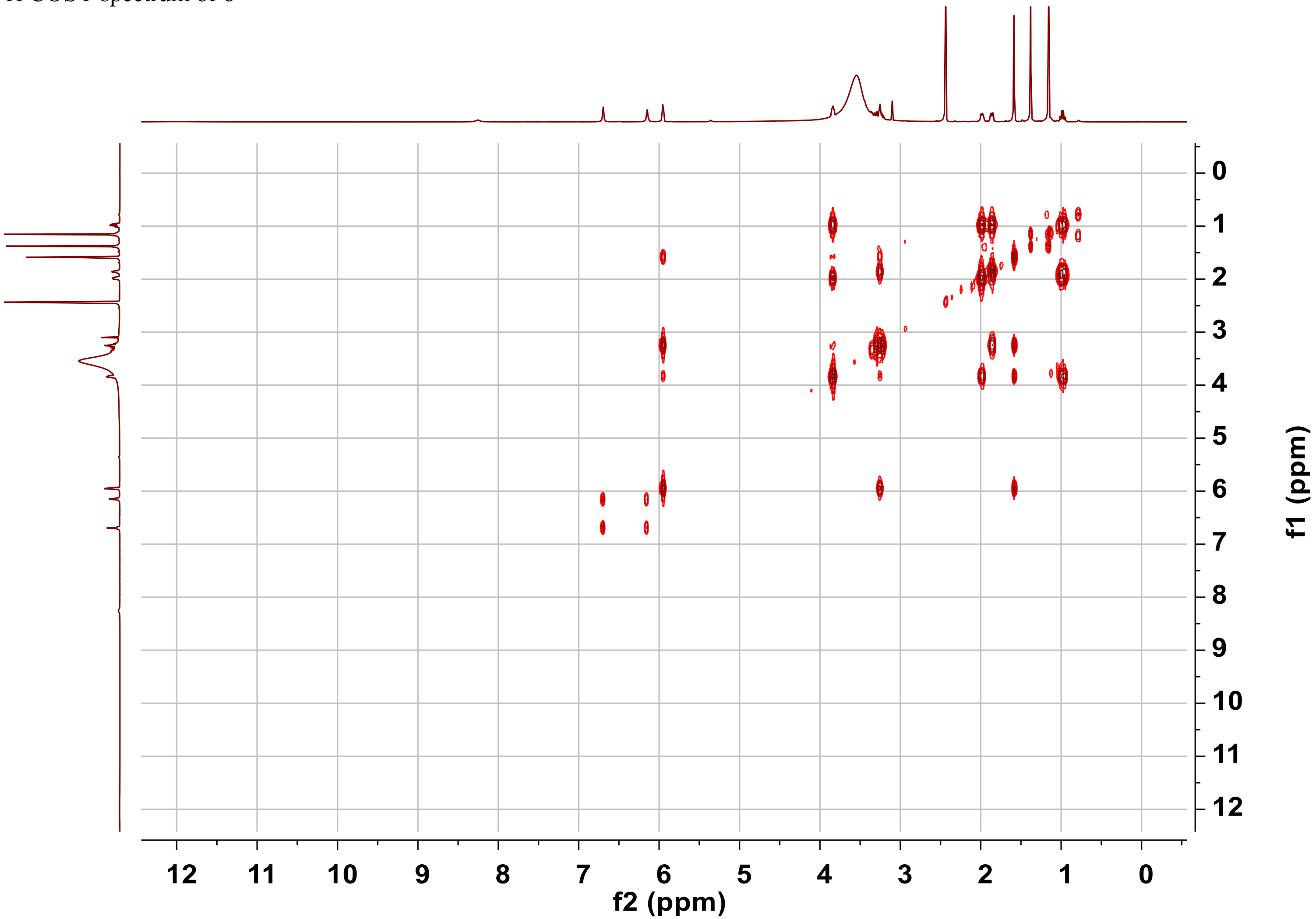

Figure S22 NOESY spectrum of **6**

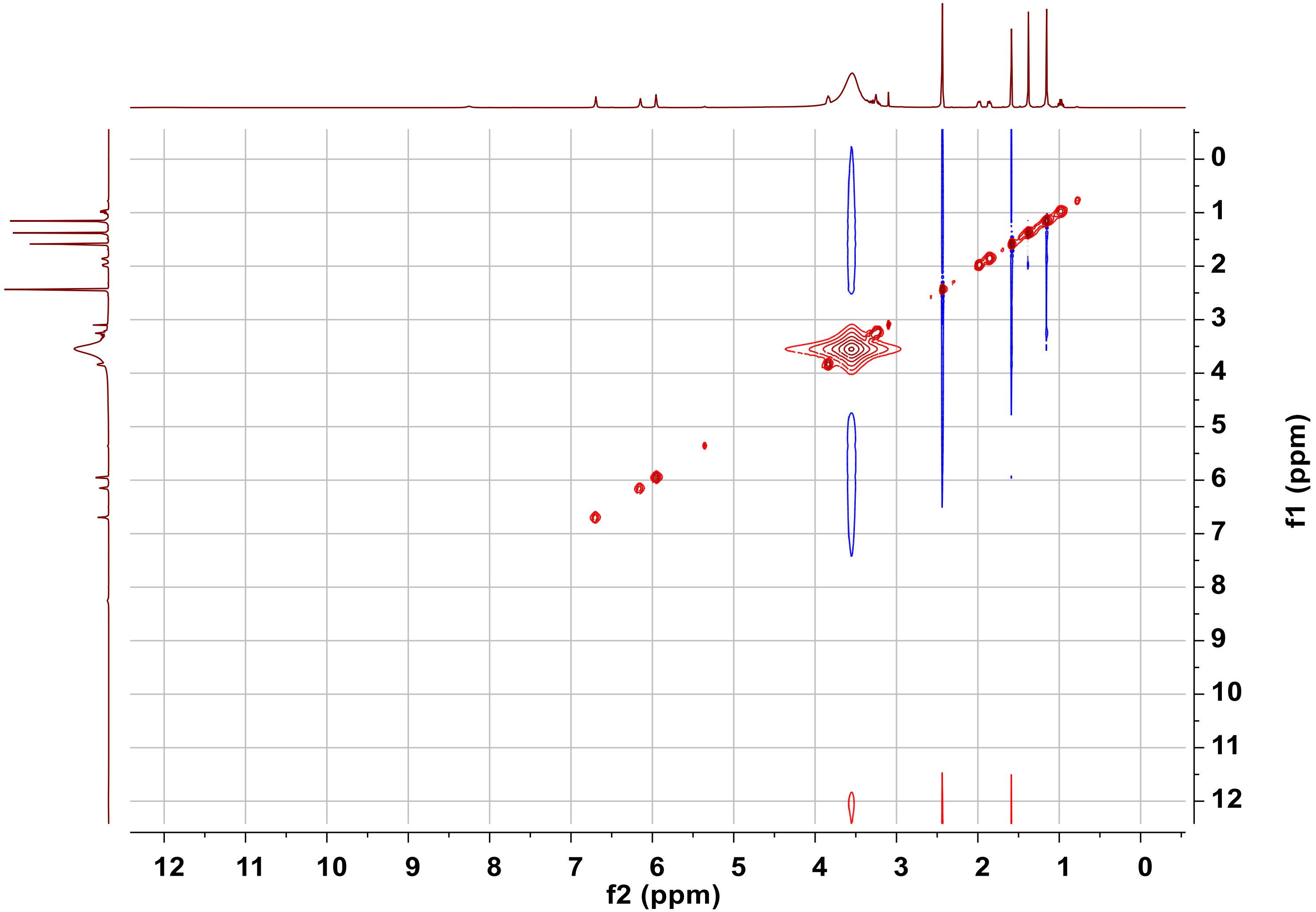

Figure S23 HRESIMS spectrum of **6**

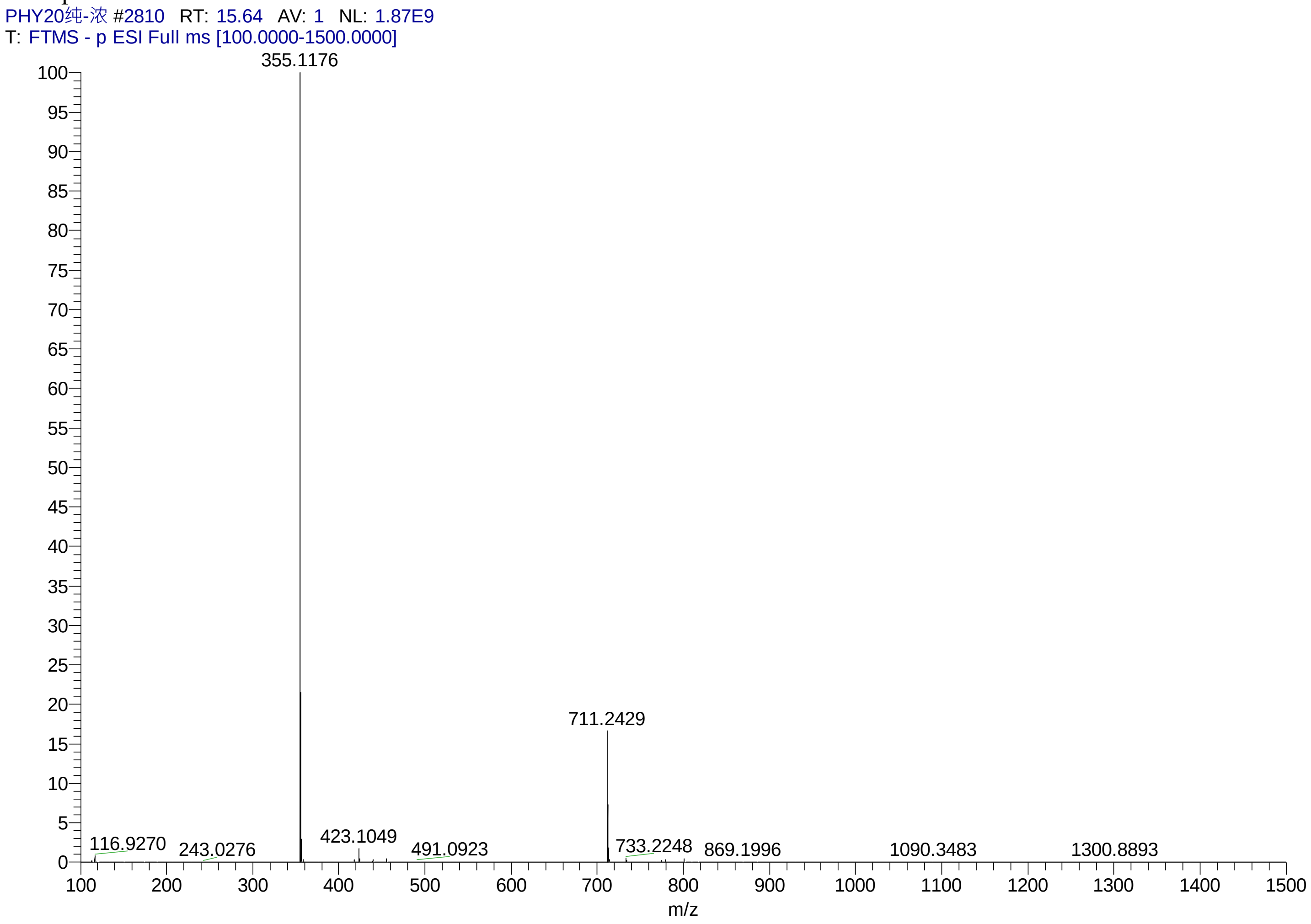

Figure S24 UV spectrum of **6**

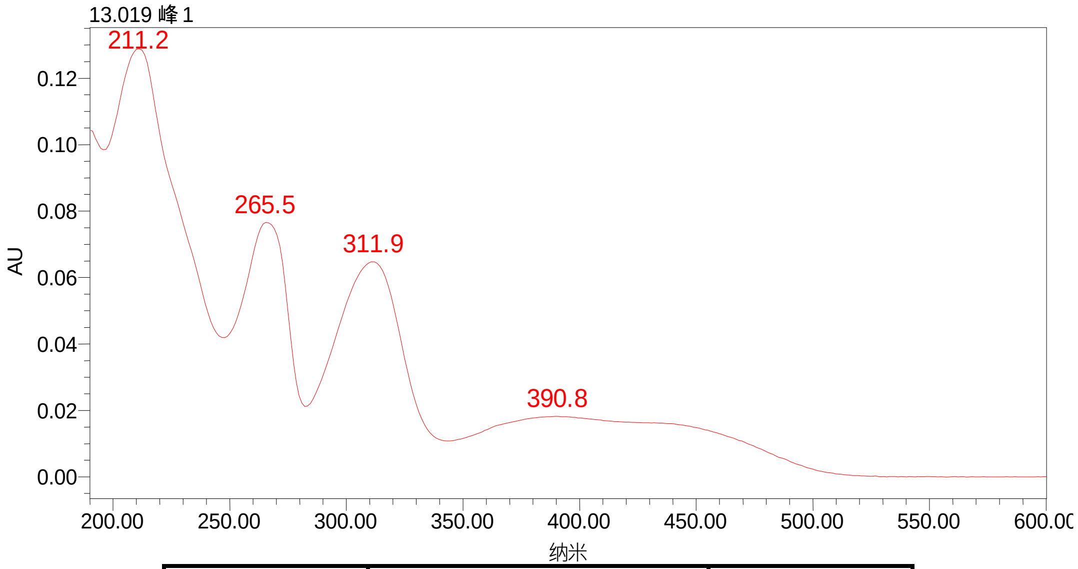

No.	Wavelength (nm)	Abs
1	211.2	0.129
2	265.5	0.077
3	312.0	0.065
4	390.8	0.018

Figure S25 ^{1}H NMR spectrum of **7** in DMSO-d_6 (600 MHz)

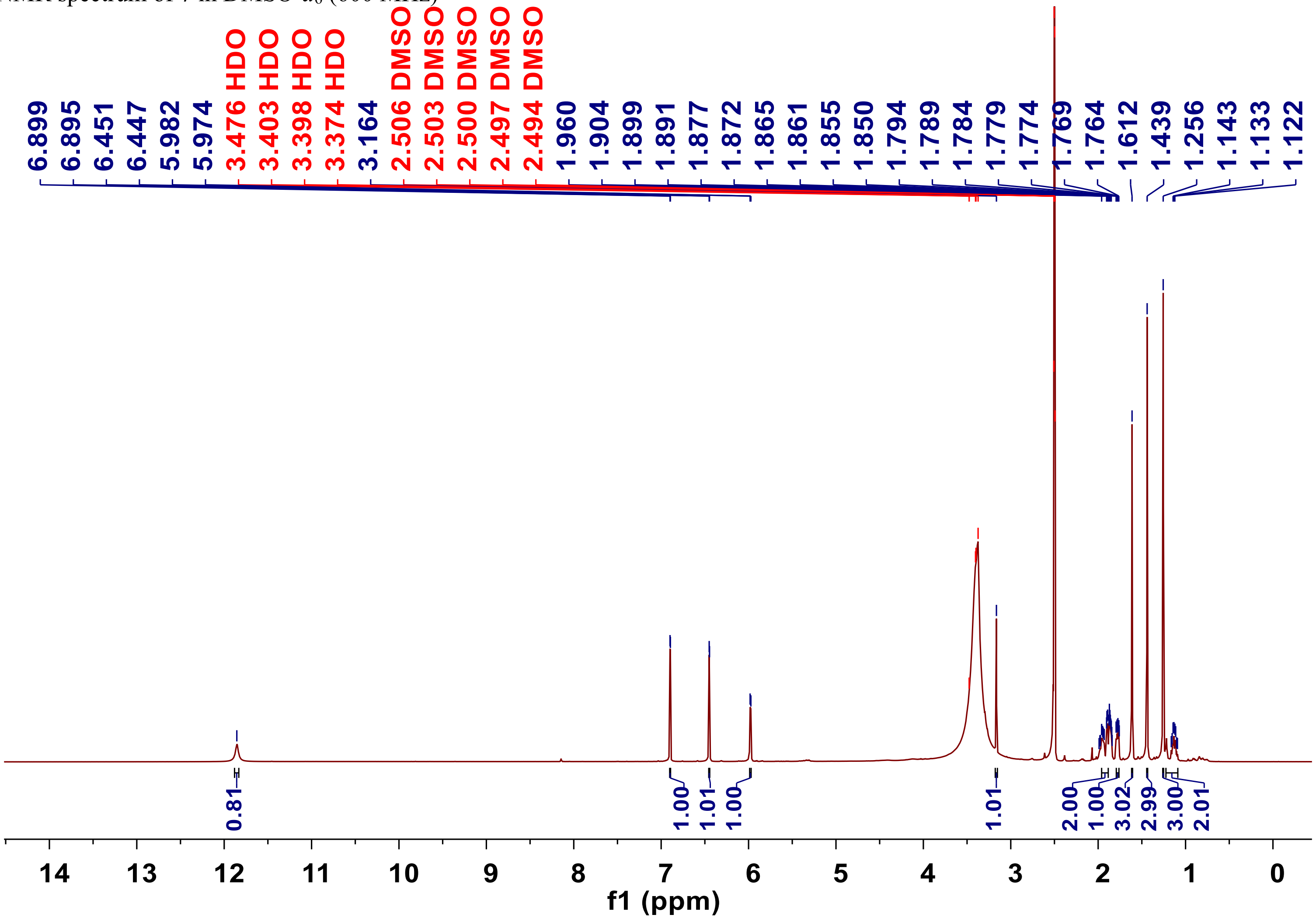

Figure S26 ^{13}C NMR spectrum of **7** in DMSO-d_6 (150 MHz)

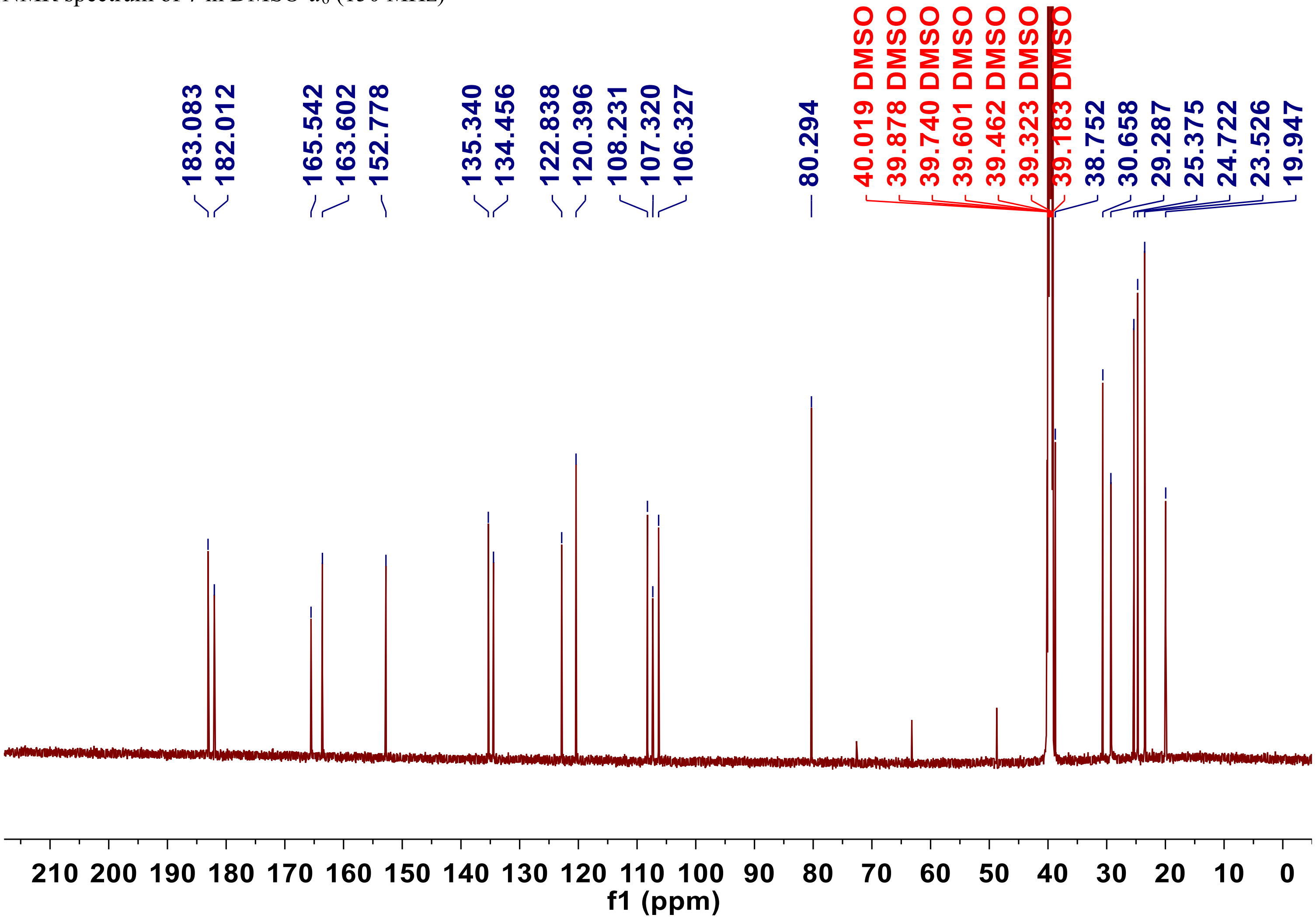

Figure S27 DEPT-135 spectrum of **7**

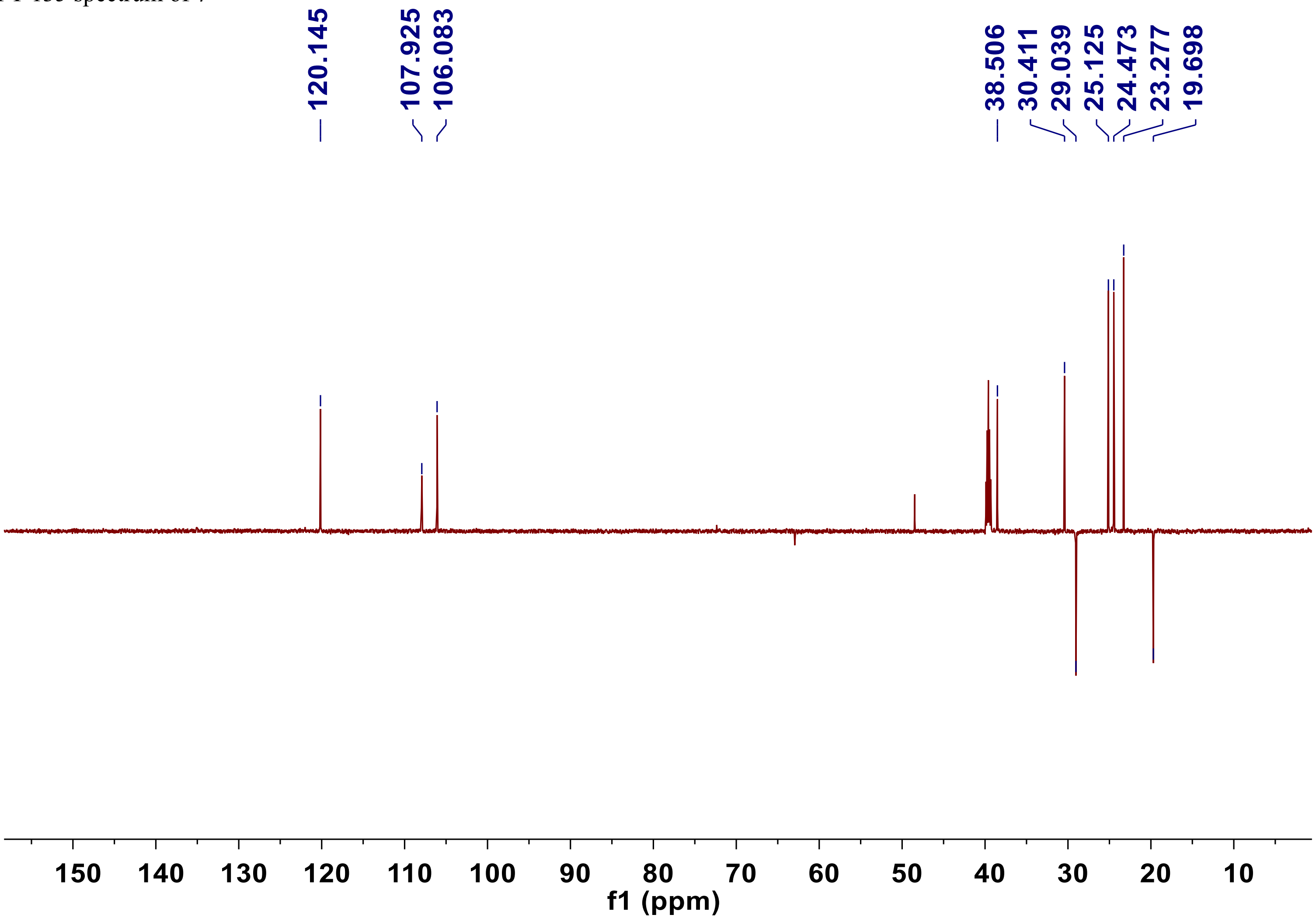

Figure S28 HSQC spectrum of **7**

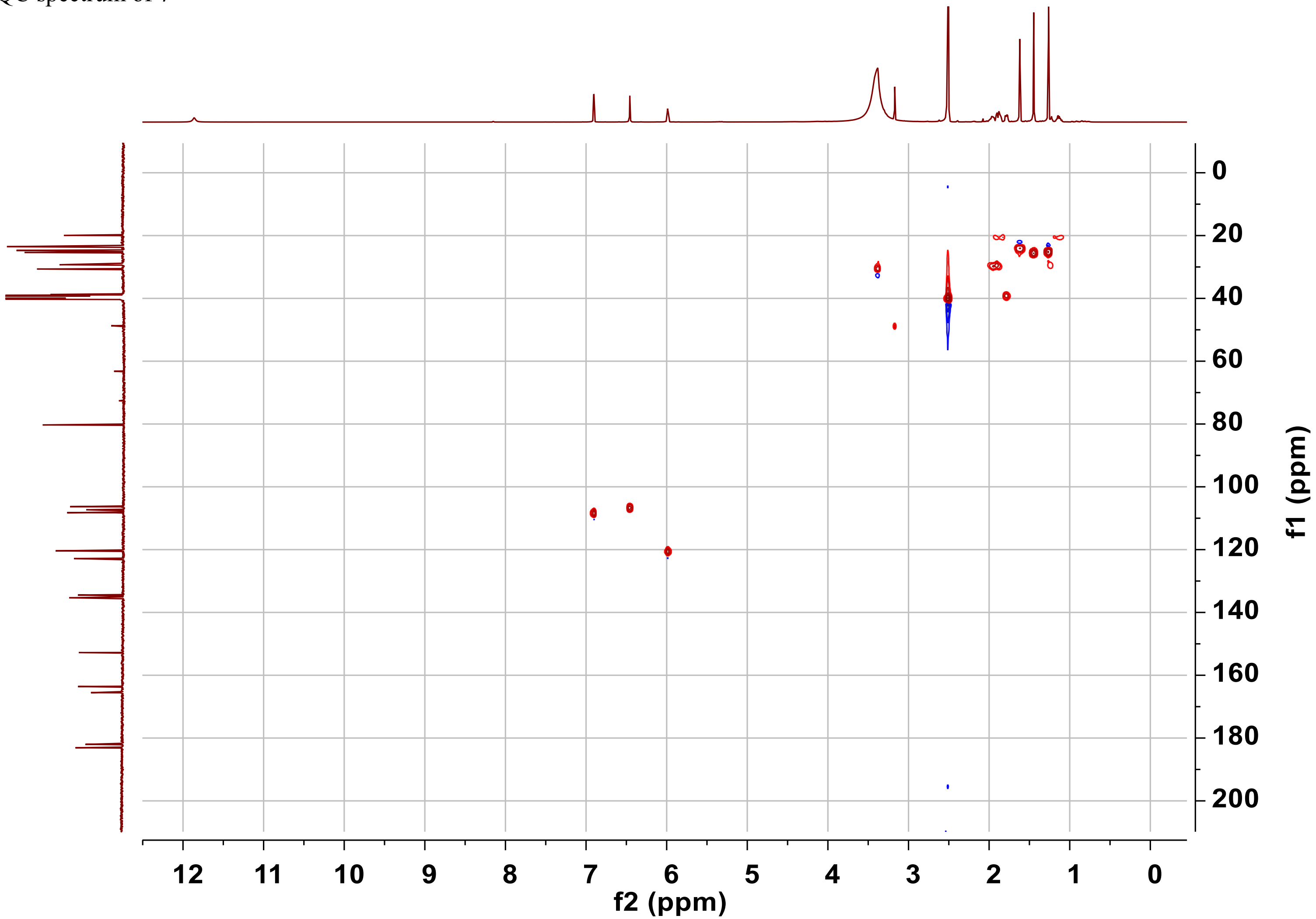

Figure S29 HMBC spectrum of **7**

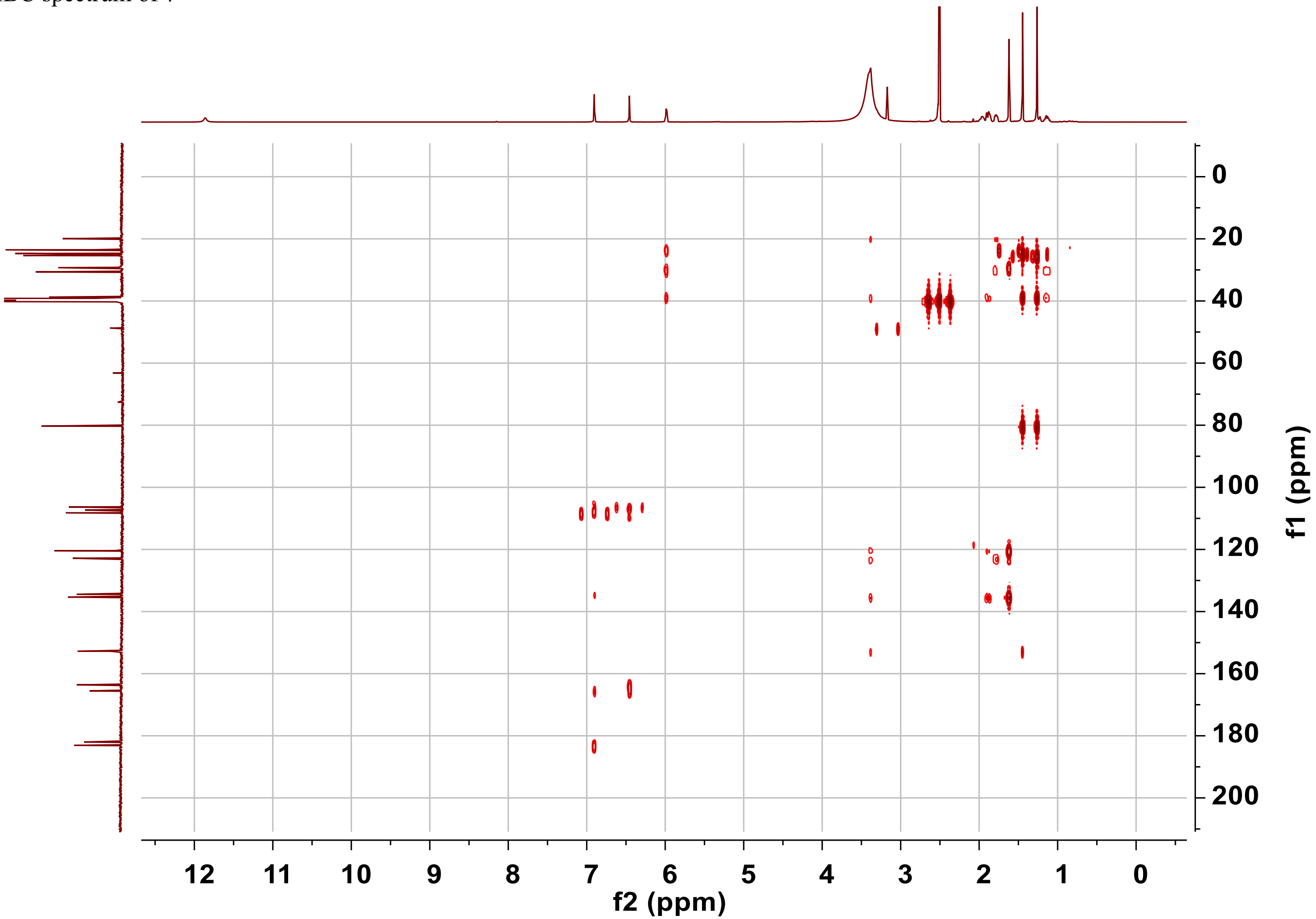

Figure S30 ^{1}H-^{1}H COSY spectrum of **7**

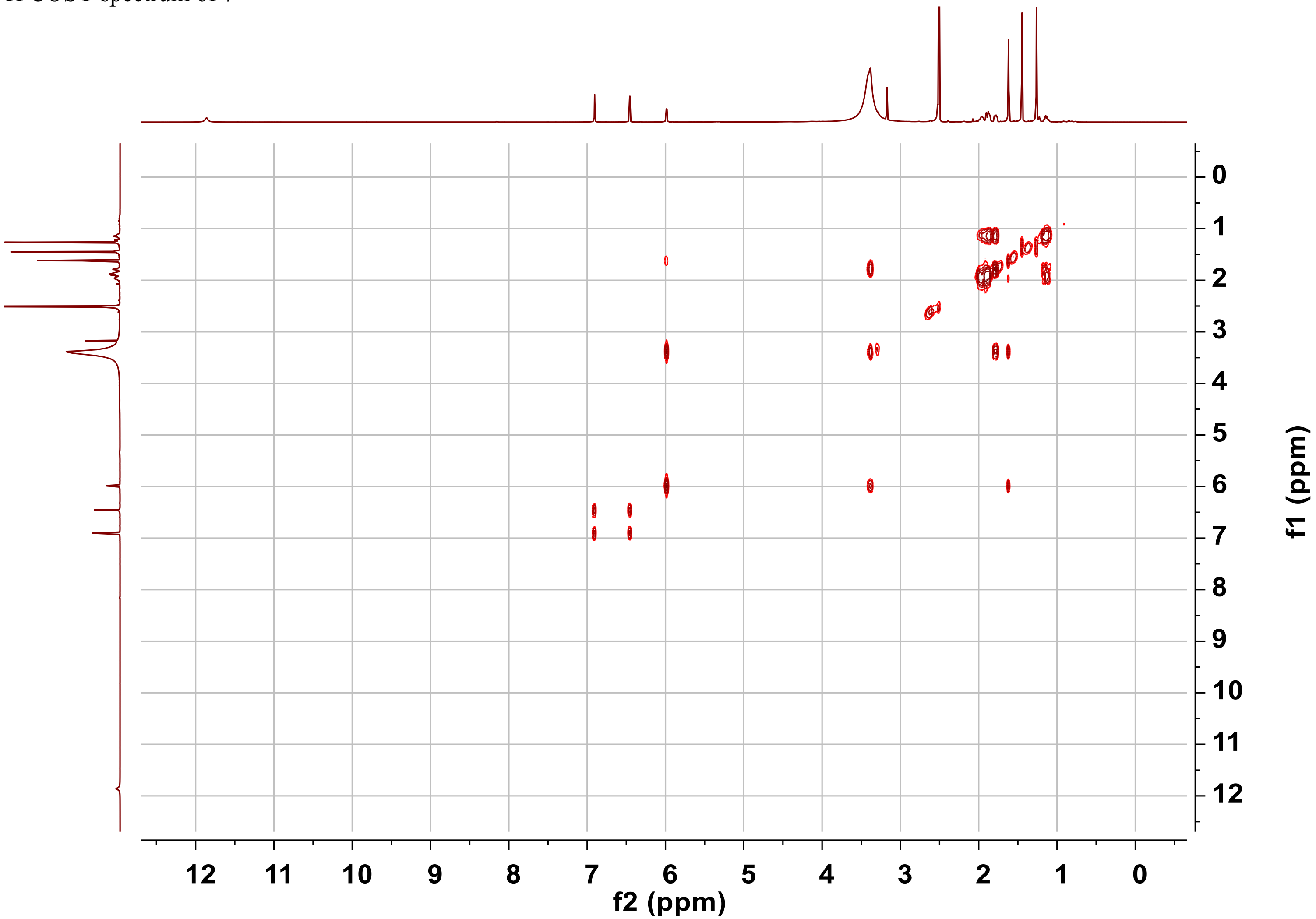

Figure S31 HRESIMS spectrum of **7**

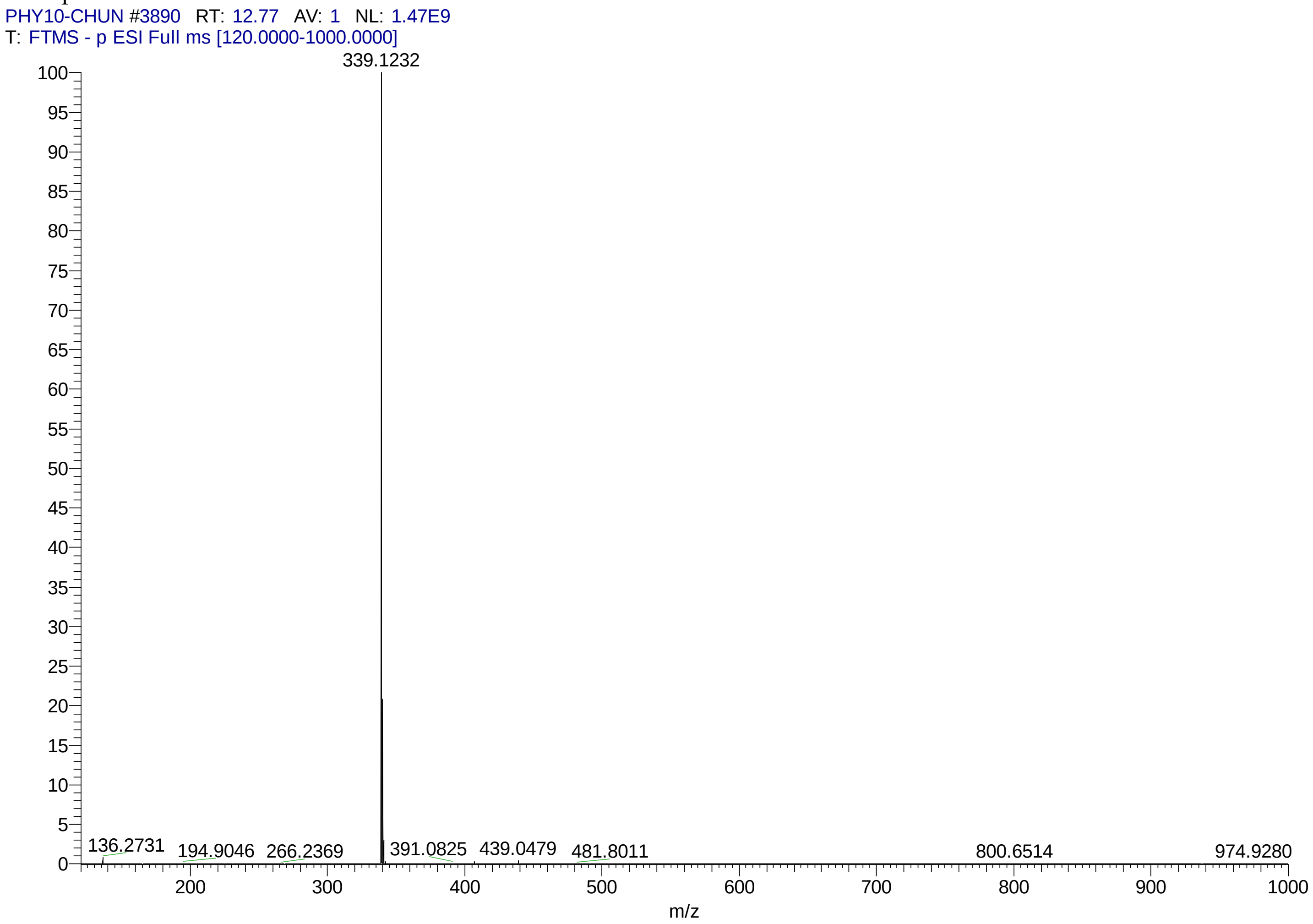

Figure S32 UV spectrum of **7**

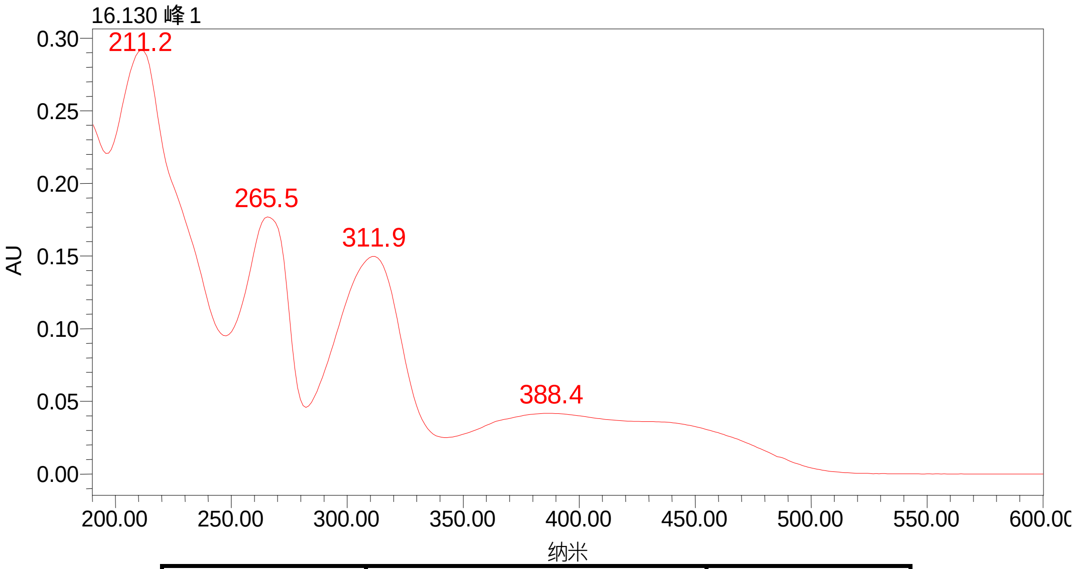

No.	Wavelength (nm)	Abs
1	211.2	0.292
2	265.5	0.177
3	312.0	0.150
4	388.4	0.042

Figure S33 ^{1}H NMR spectrum of **8** in DMSO-d_6 (600 MHz)

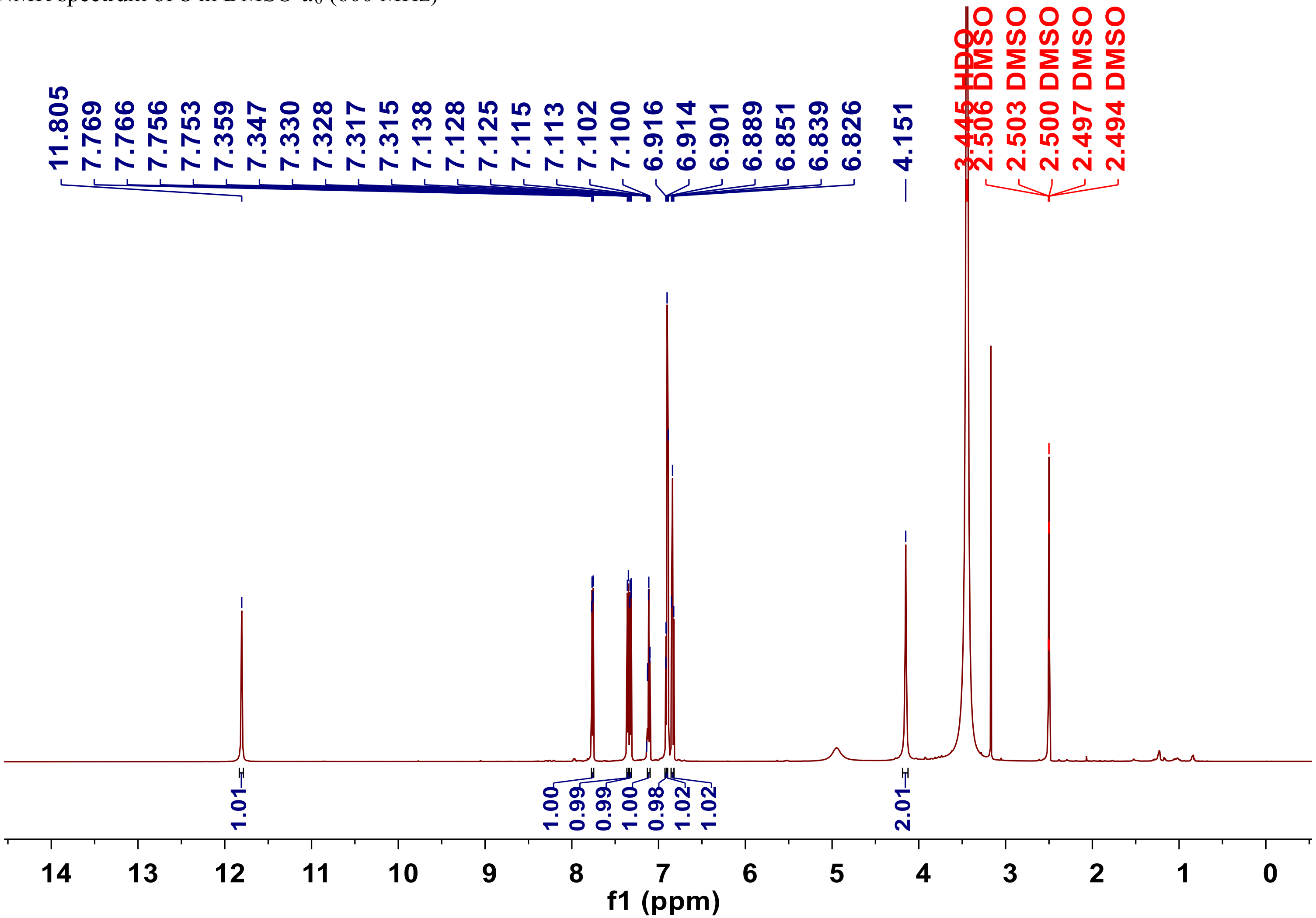

Figure S34 ^{13}C NMR spectrum of **8** in DMSO-d_6 (150 MHz)

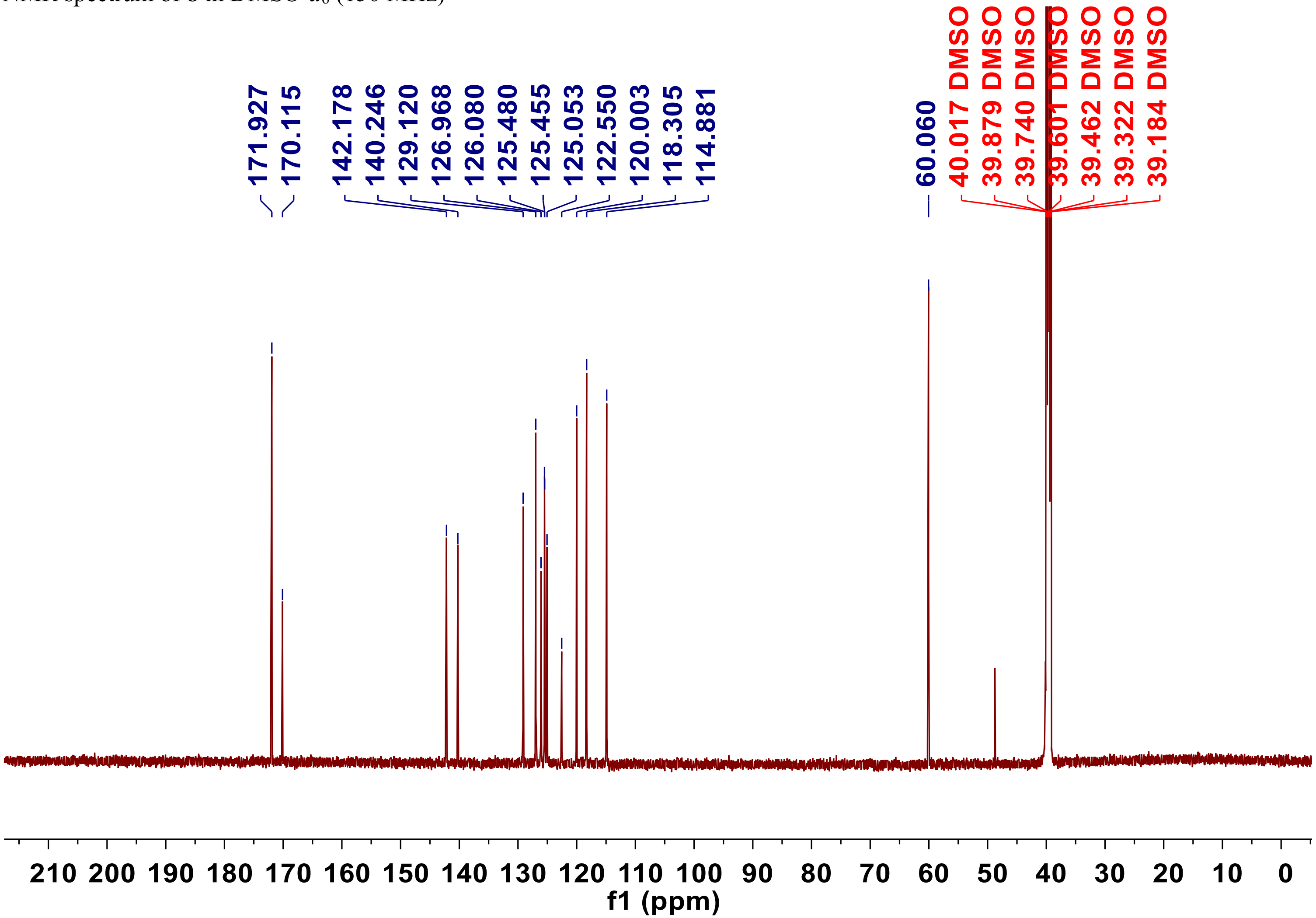

Figure S35 DEPT-135 spectrum of **8**

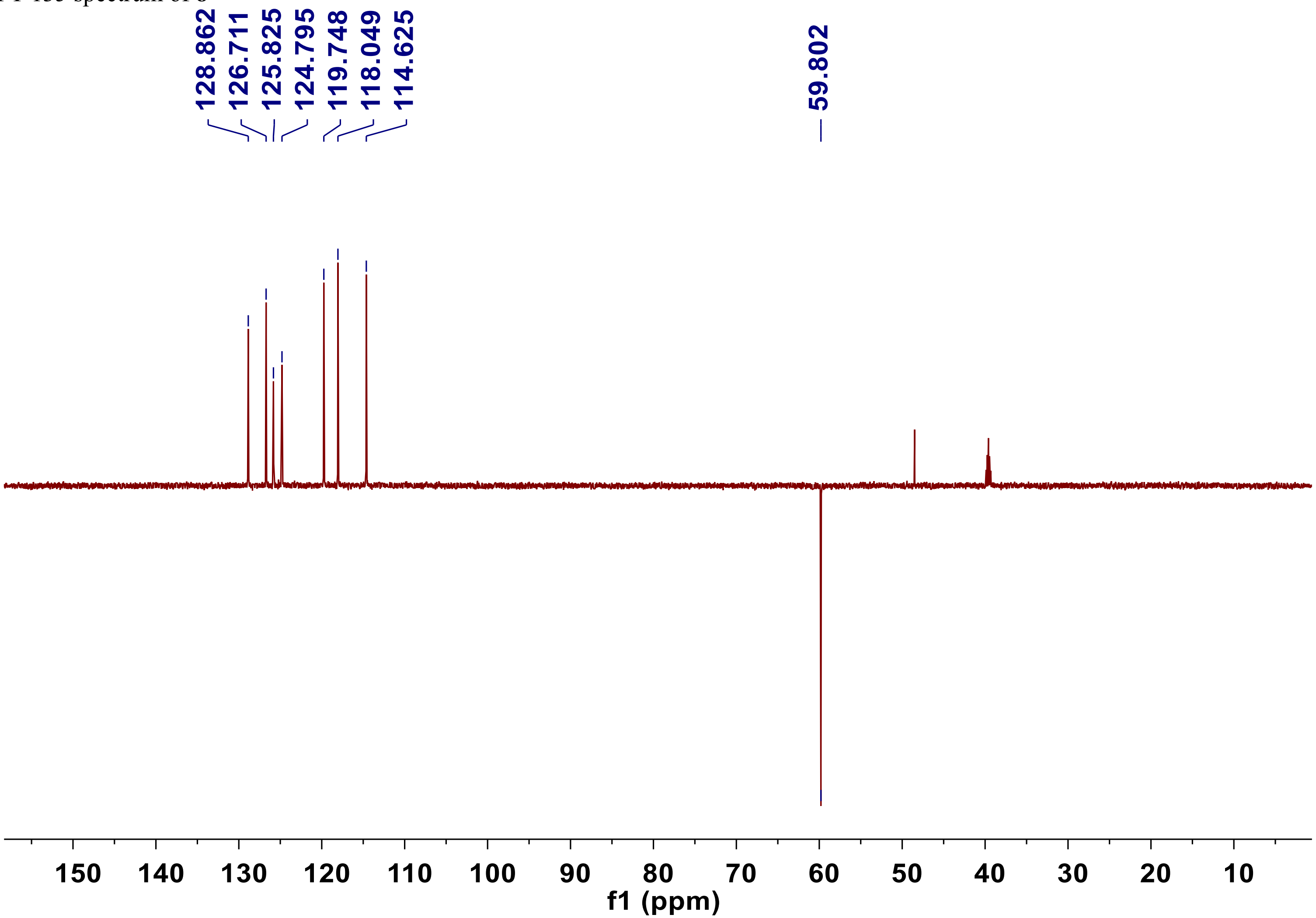

Figure S36 HSQC spectrum of **8**

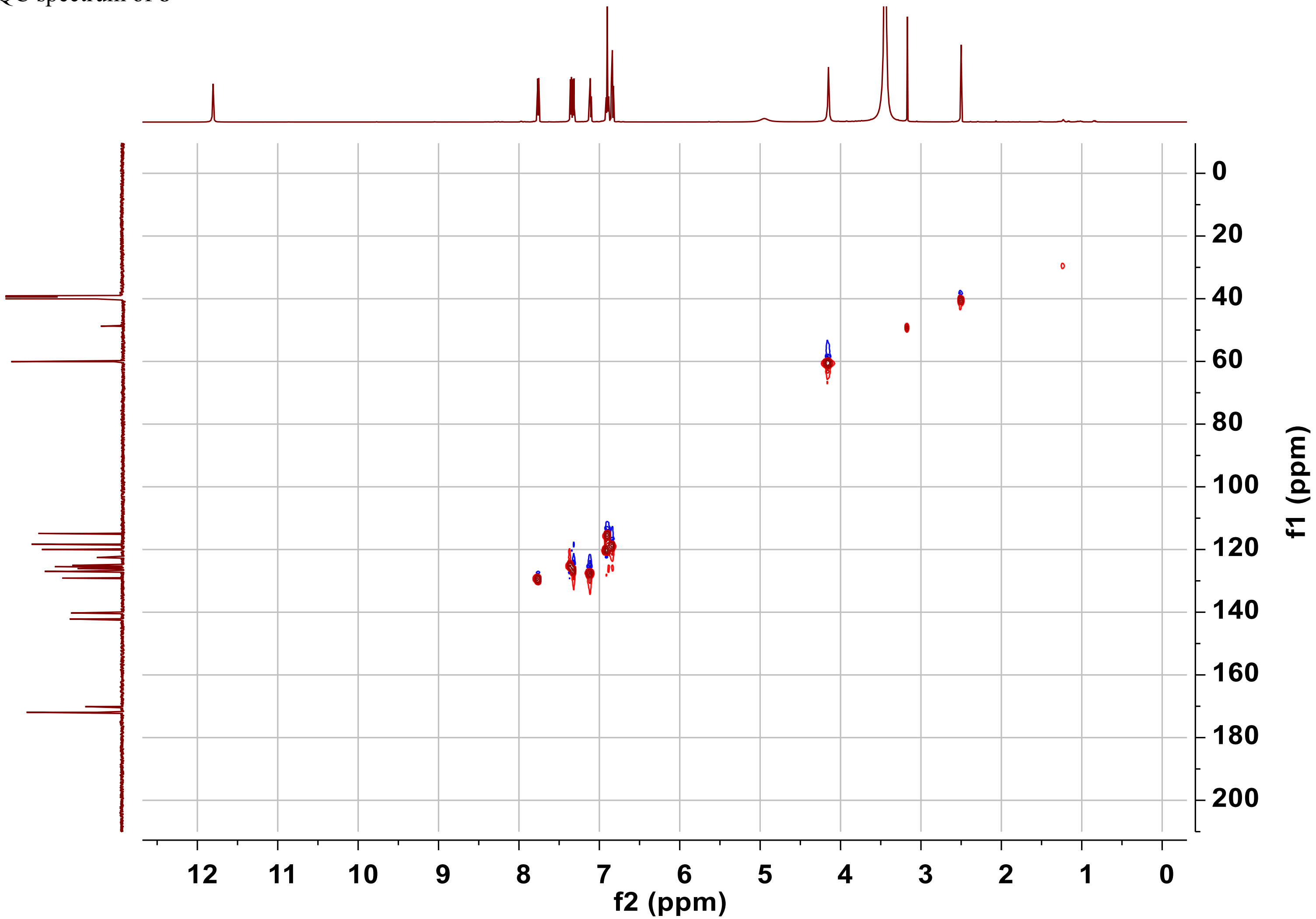

Figure S37 HMBC spectrum of **8**

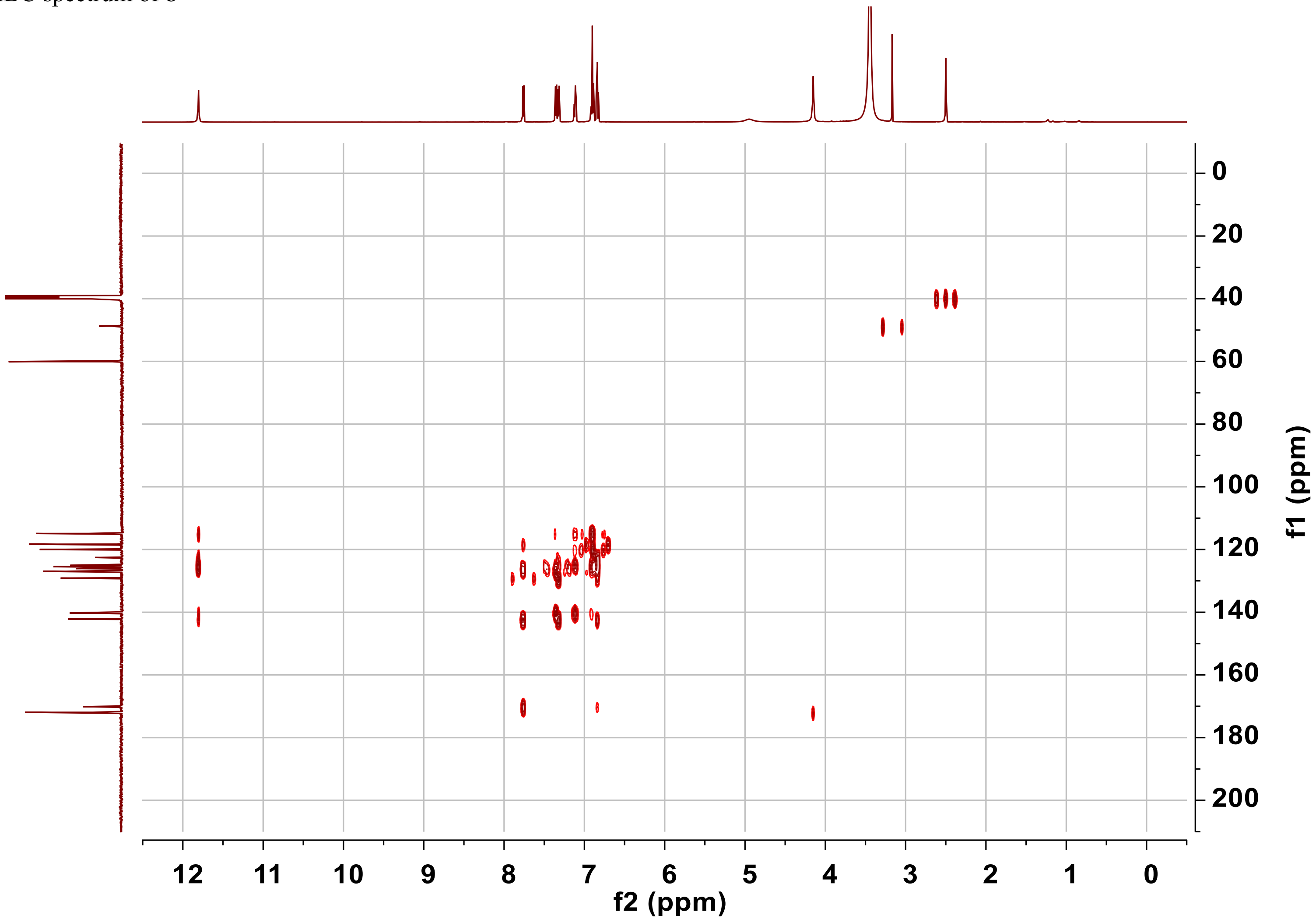

Figure S38 HRESIMS spectrum of **8**

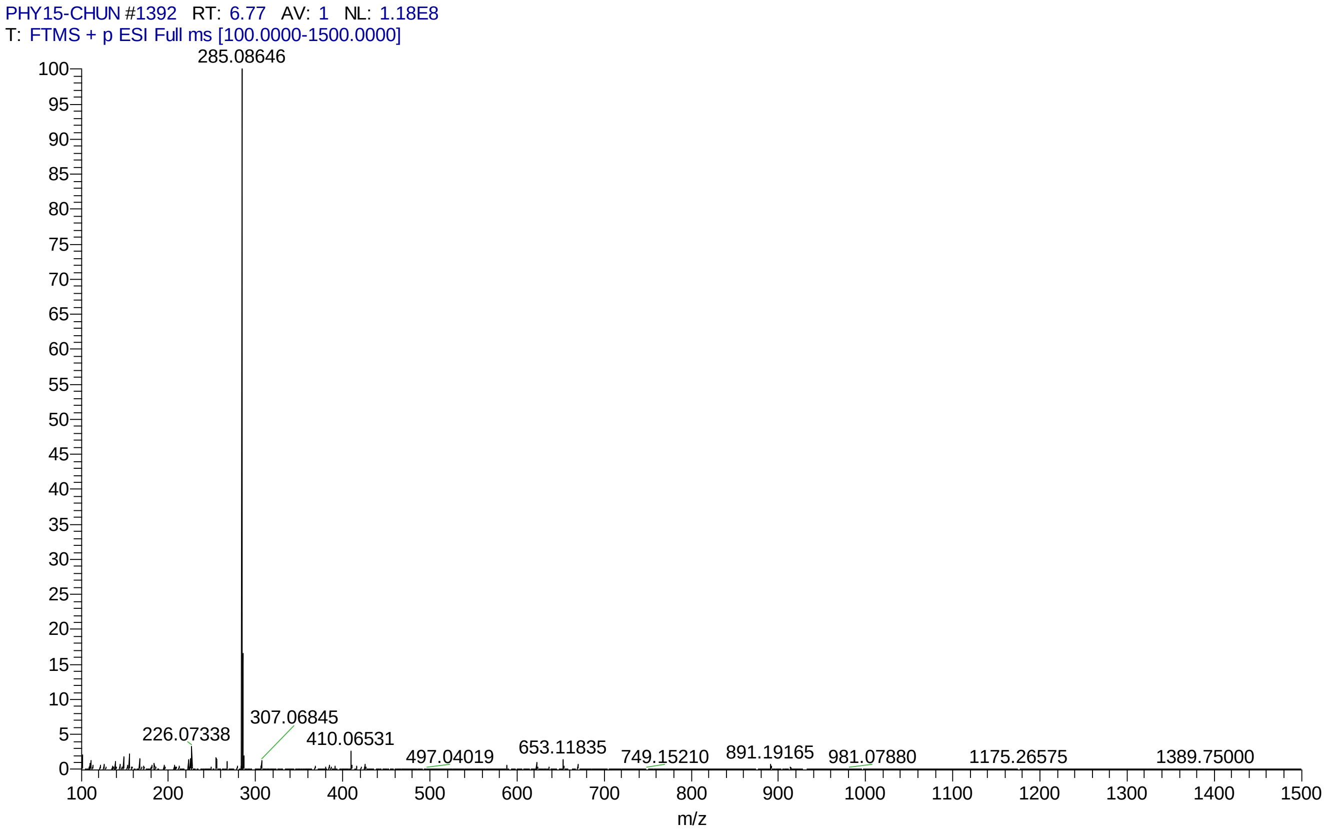

Figure S39 UV spectrum of **8**

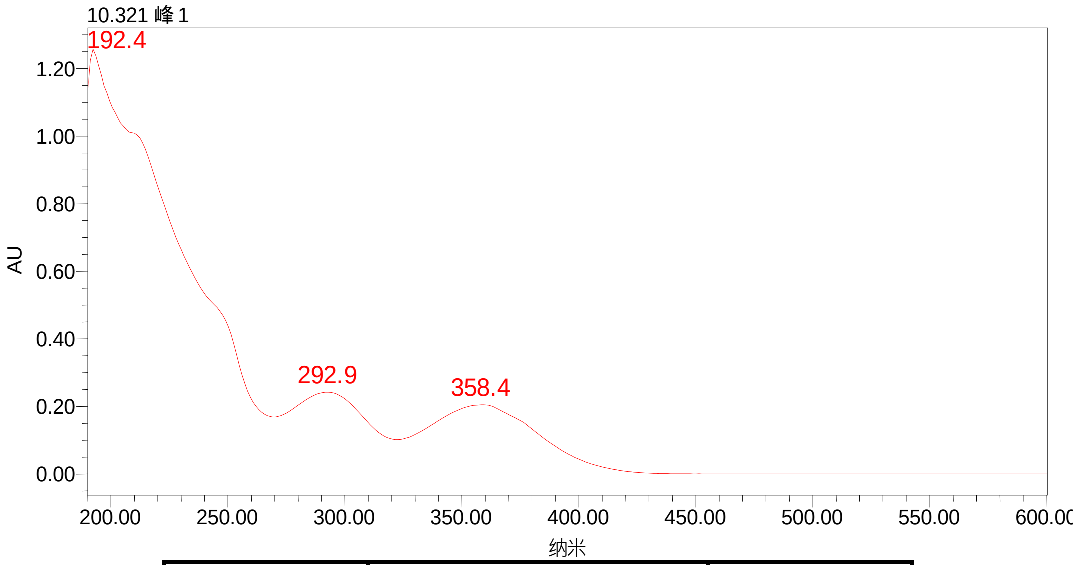

No.	Wavelength (nm)	Abs
1	192.37	1.25753
2	292.88	0.24223
3	358.42	0.20483

Figure S40 ^{1}H NMR spectrum of **9** in DMSO-d_6 (600 MHz)

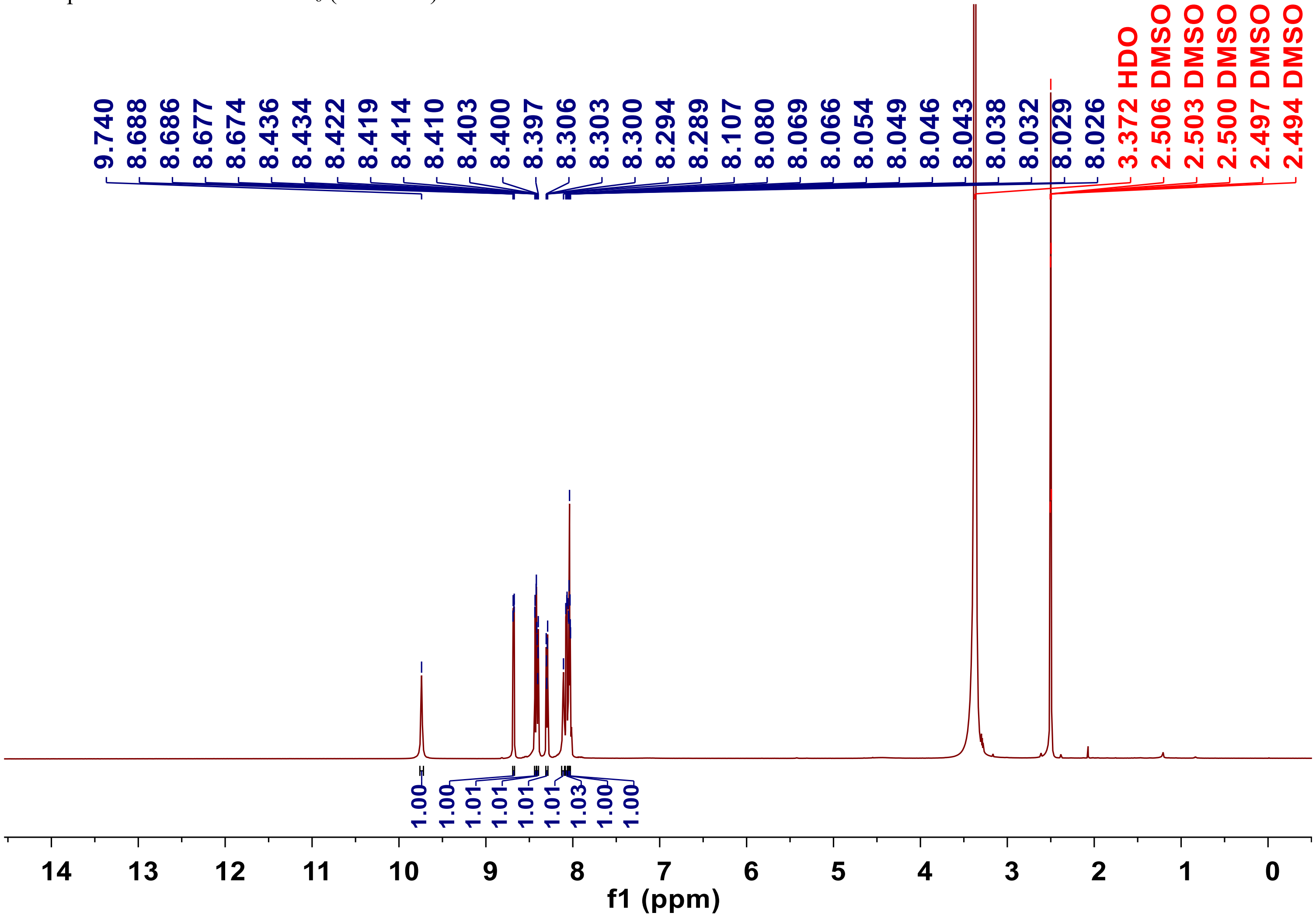

Figure S41 ^{13}C NMR spectrum of **9** in DMSO-d_6 (600 MHz)

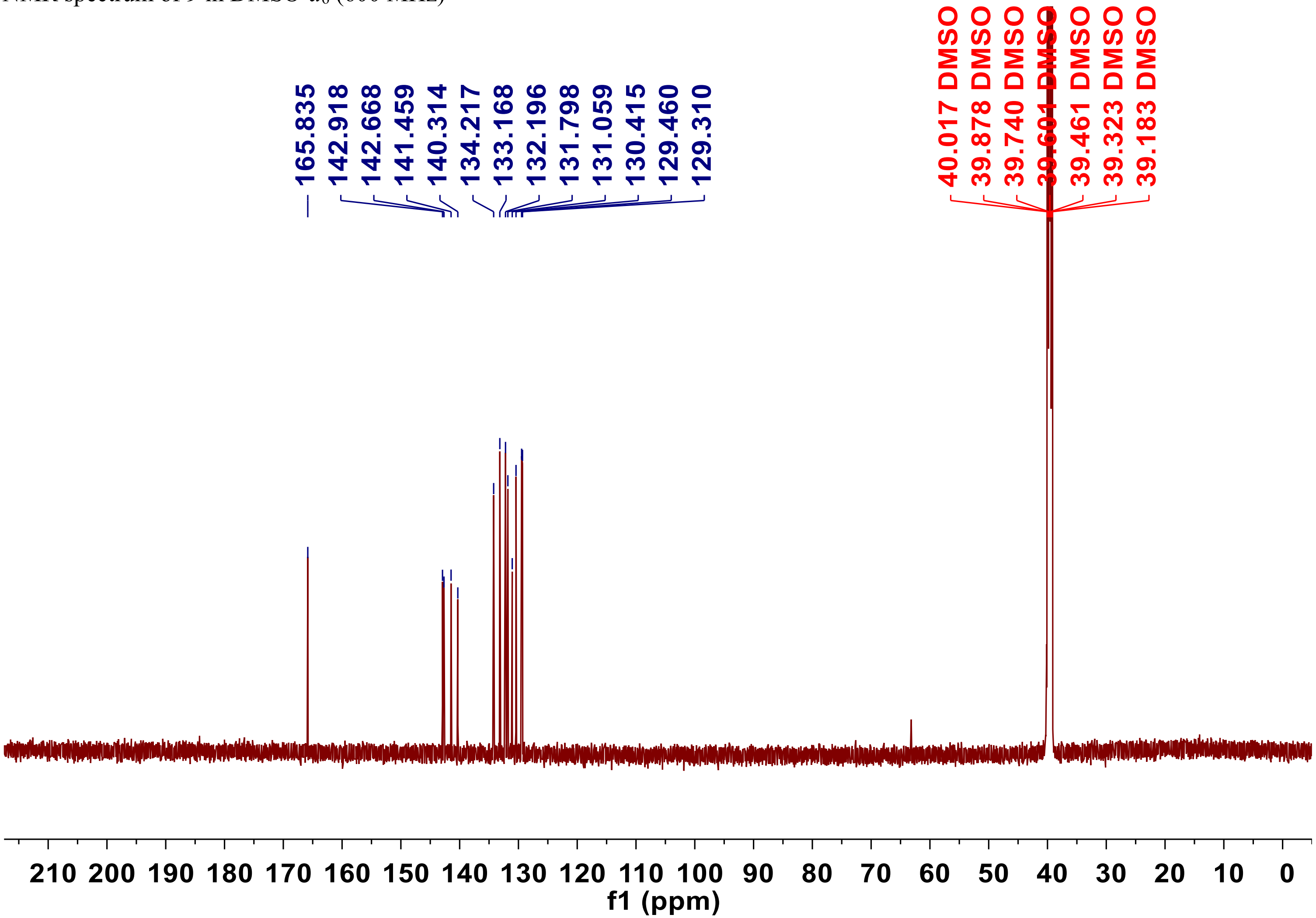

Figure S42 HRESIMS spectrum of **9**

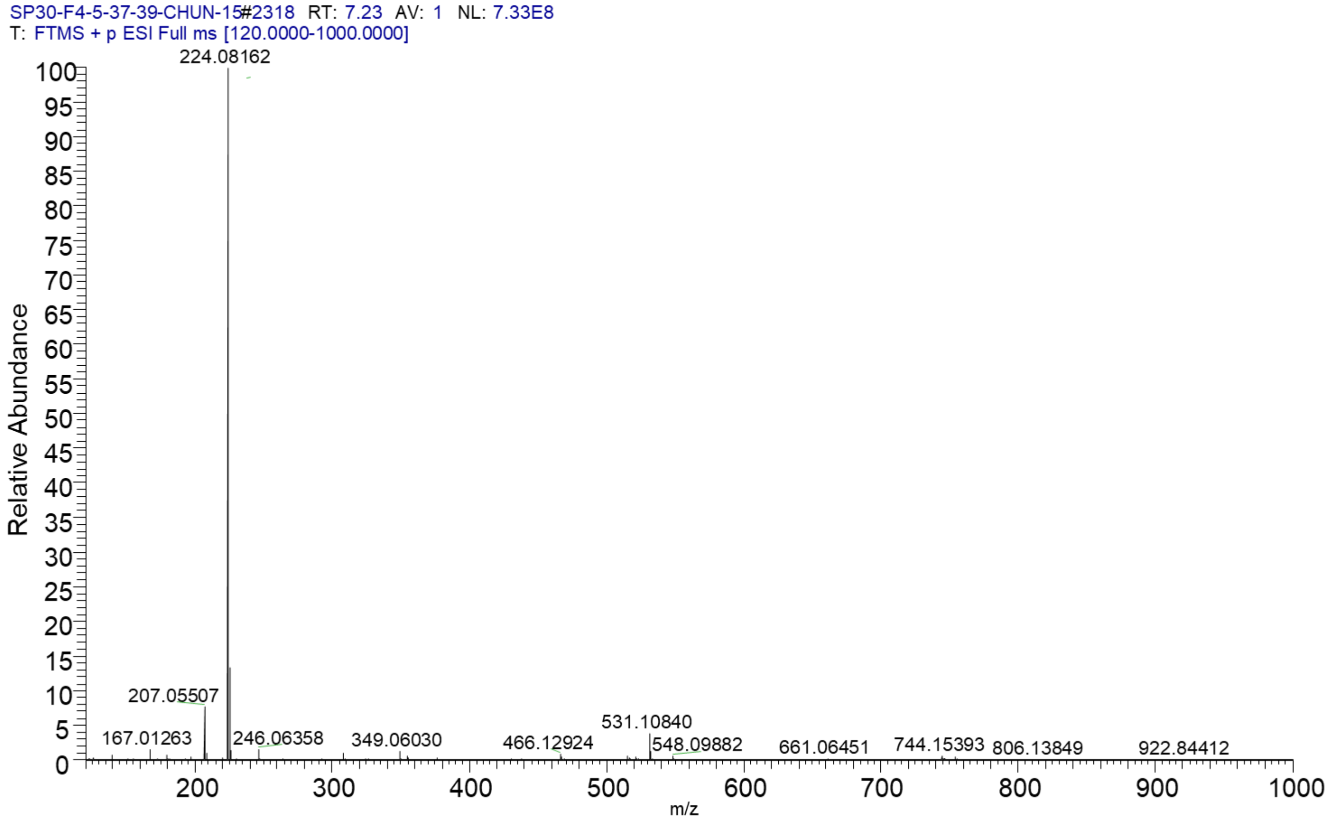

Figure S43 UV spectrum of **9**

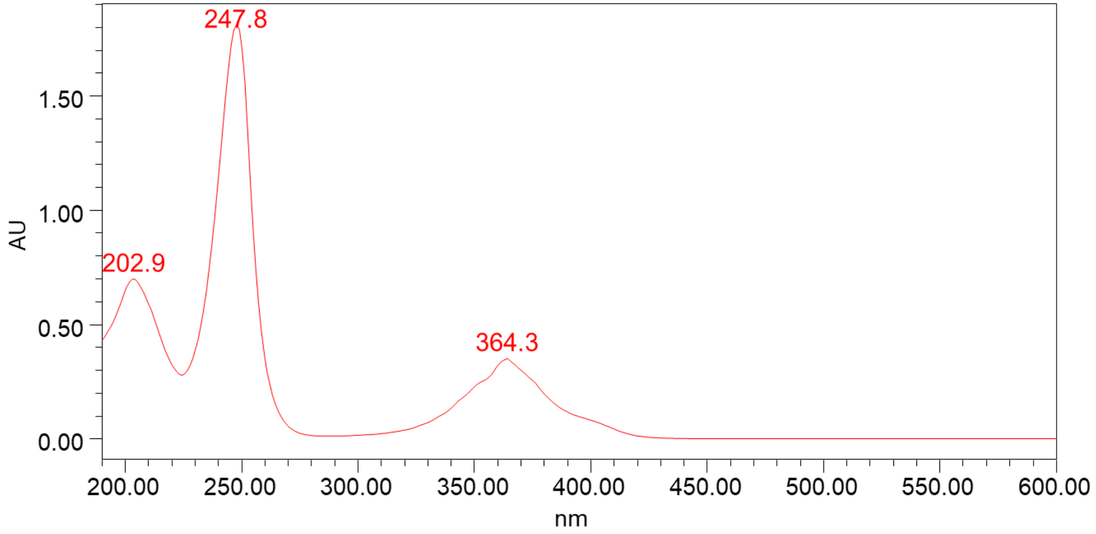

NO.	Wavelength	Abs
1	202.9	0.69835
2	247.8	1.81347
3	364.3	0.35122

Figure S44 ^{1}H NMR spectrum of **10** in DMSO-d_6 (600 MHz)

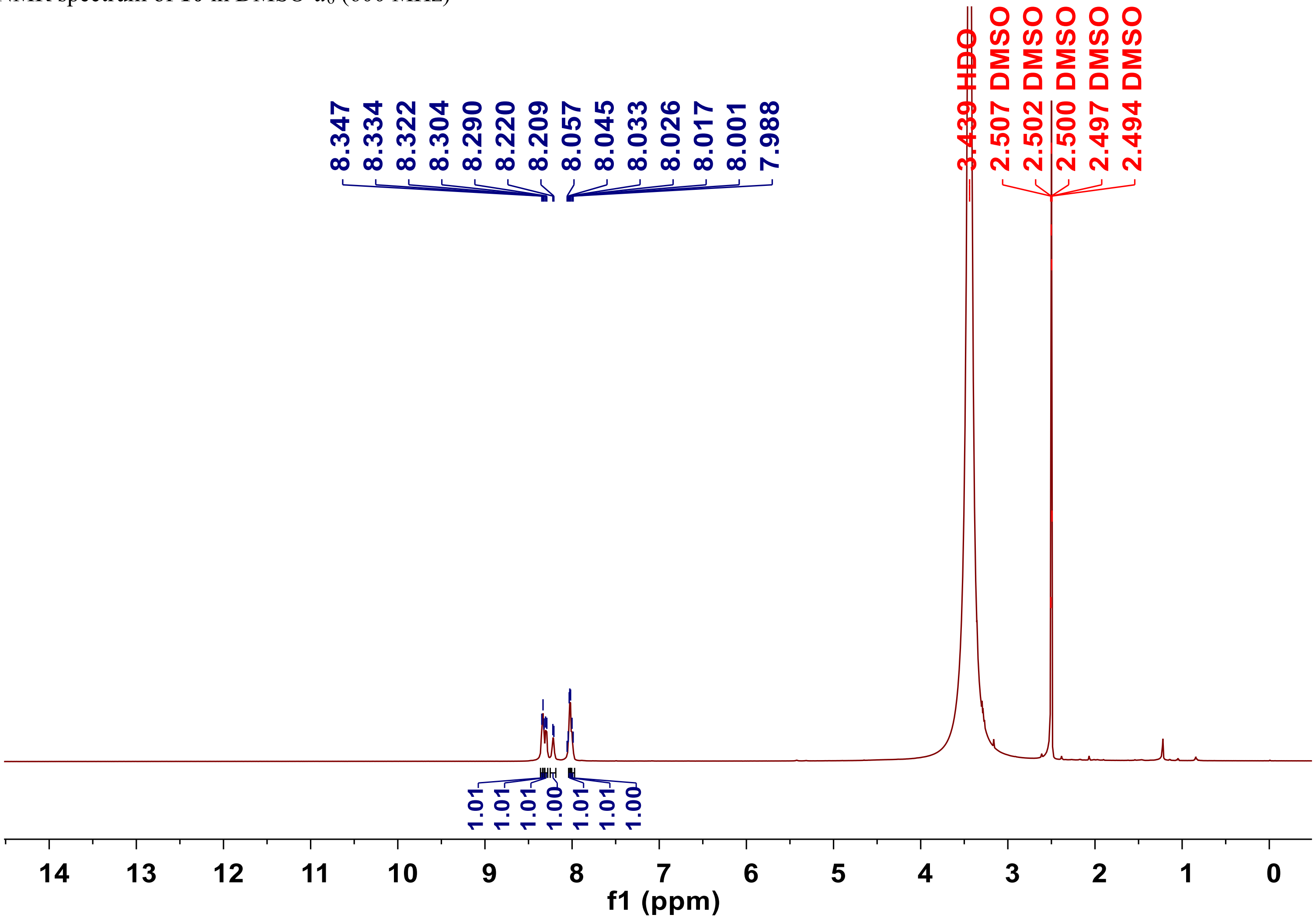

Figure S45 ^{13}C NMR spectrum of **10** in DMSO-d_6 (150 MHz)

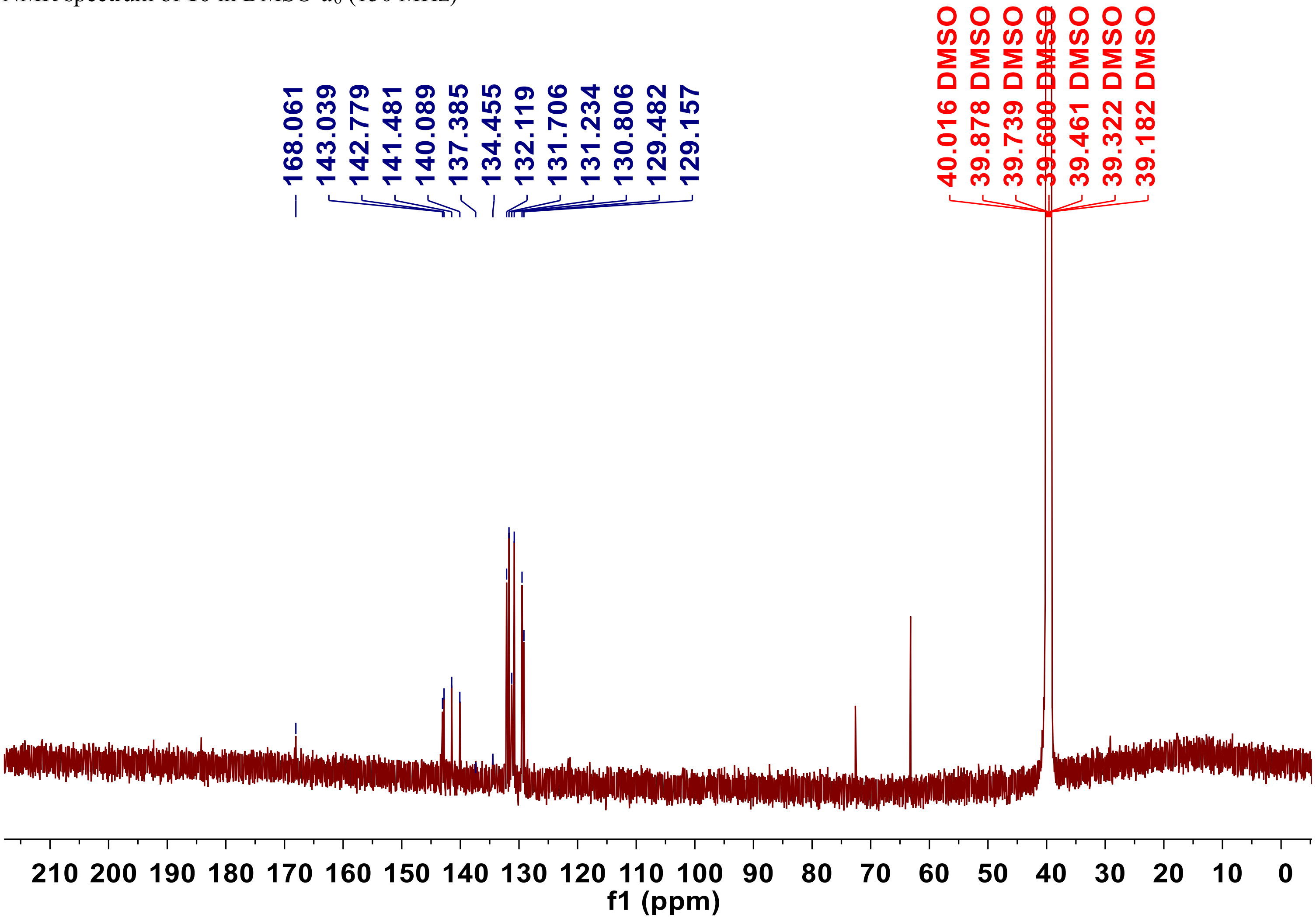

Figure S46 HMBC spectrum of **10**

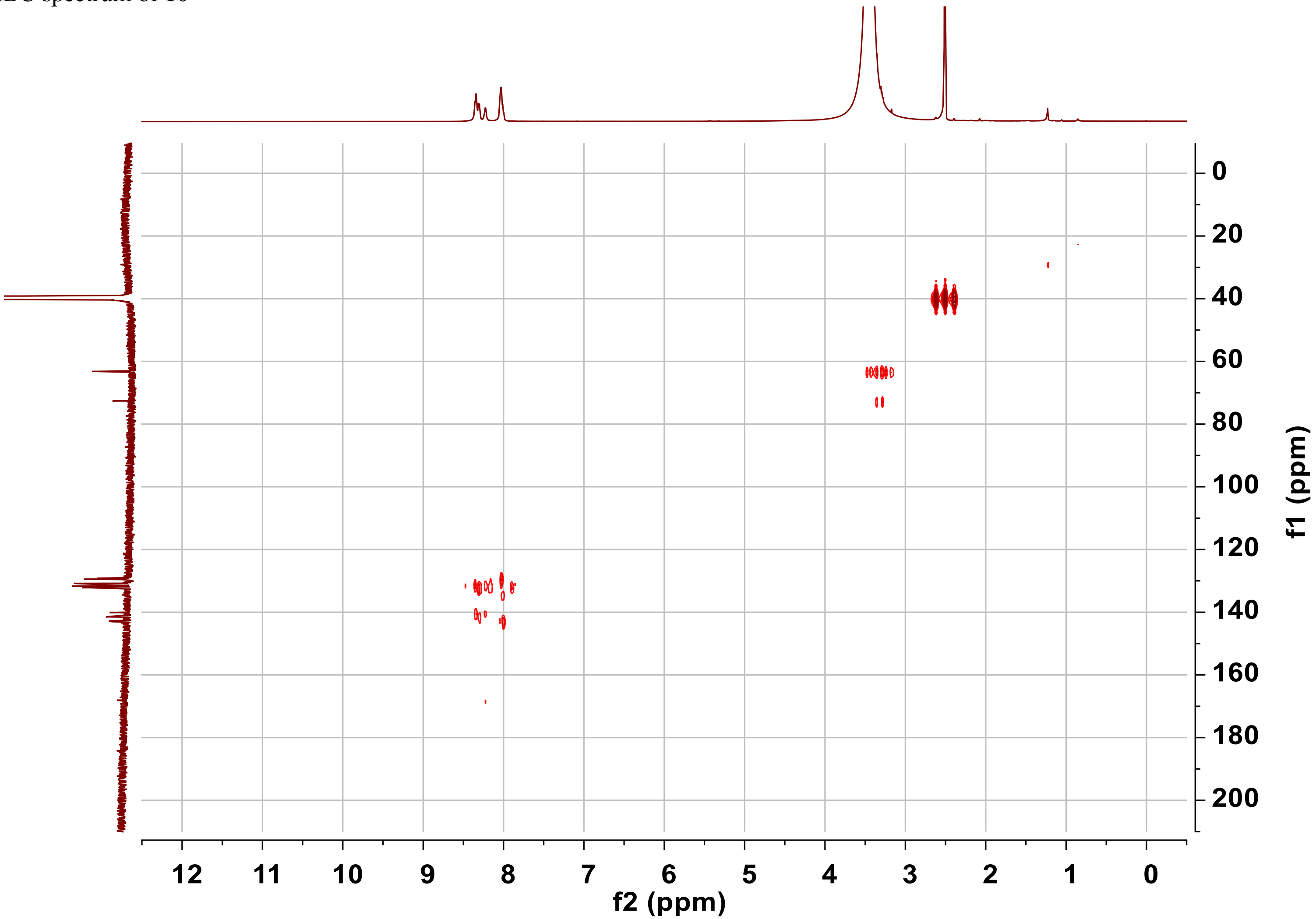

Figure S47 HRESIMS spectrum of **10**

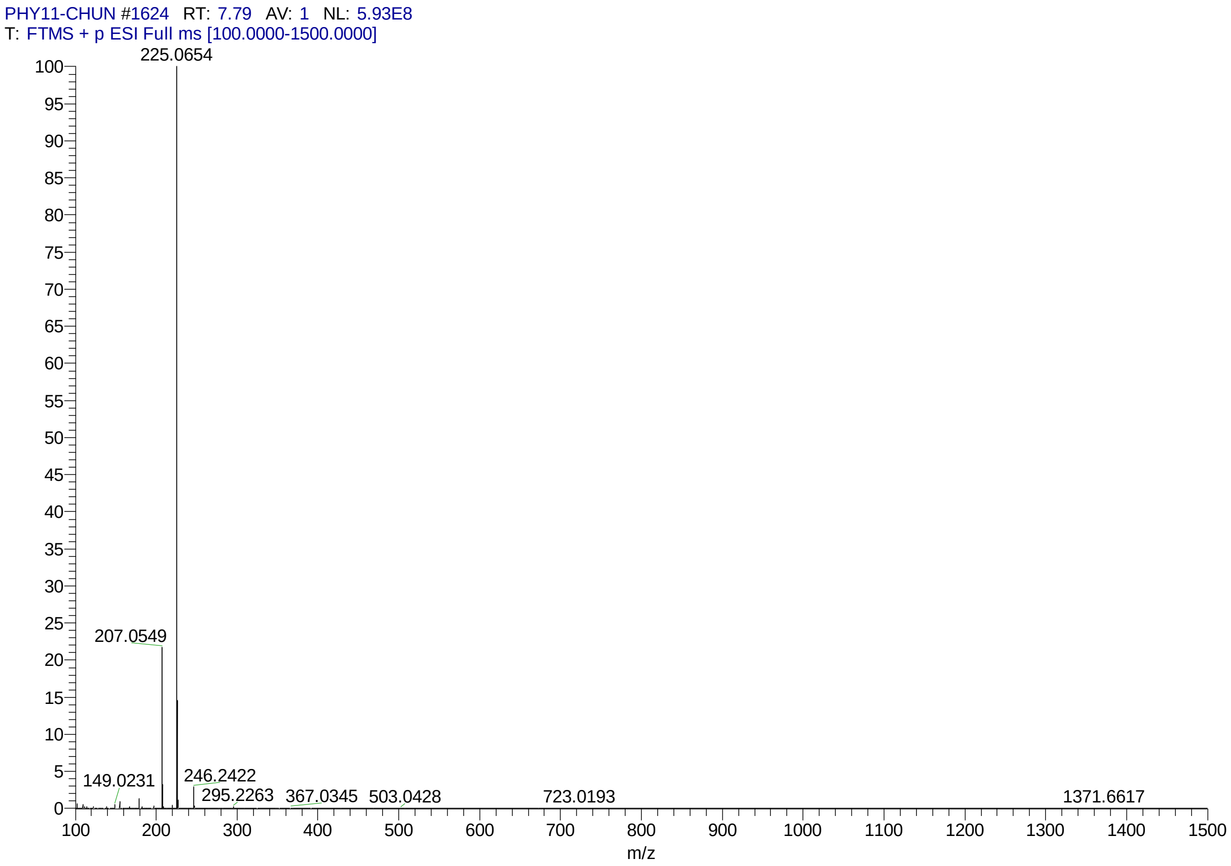

Figure S48 UV spectrum of **10**

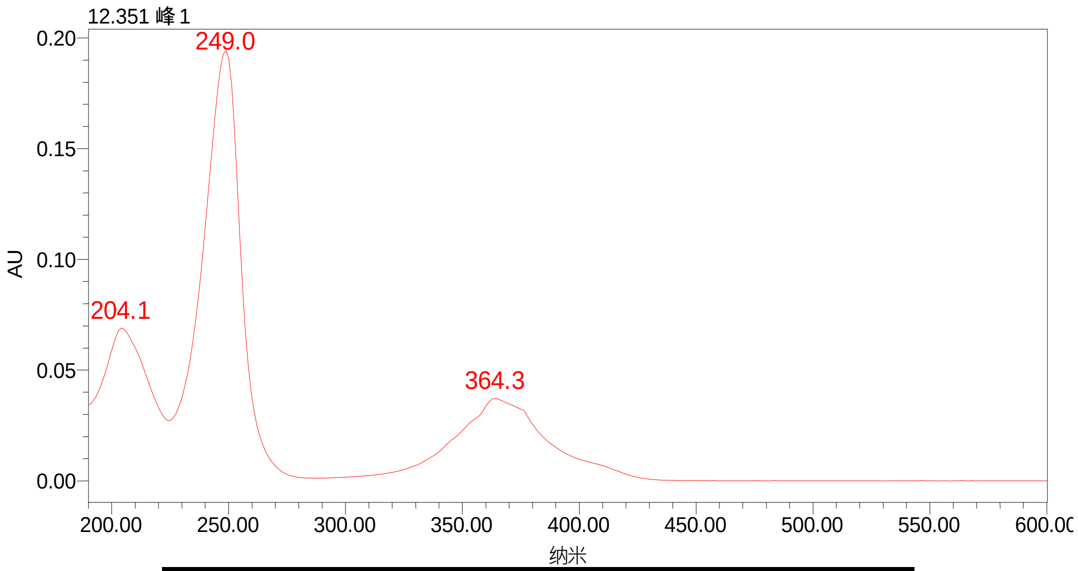

No.	Wavelength (nm)	Abs
1	204.12	0.06897
2	248.95	0.19429
3	364.32	0.03733

Figure S49 ^{1}H NMR spectrum of **11** in DMSO-d_6 (500 MHz)

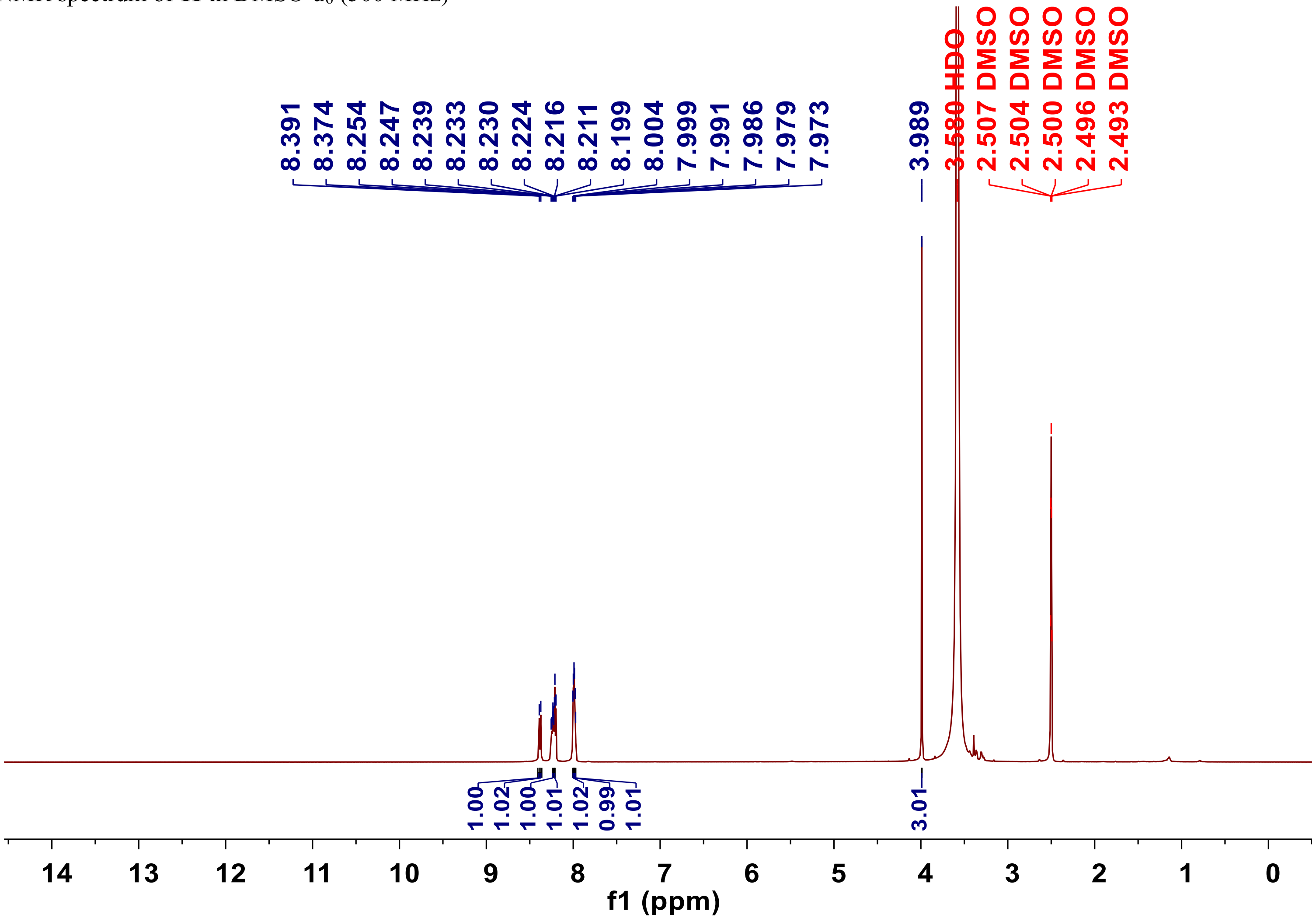

Figure S50 ^{13}C NMR spectrum of **11** in DMSO-d_6 (125 MHz)

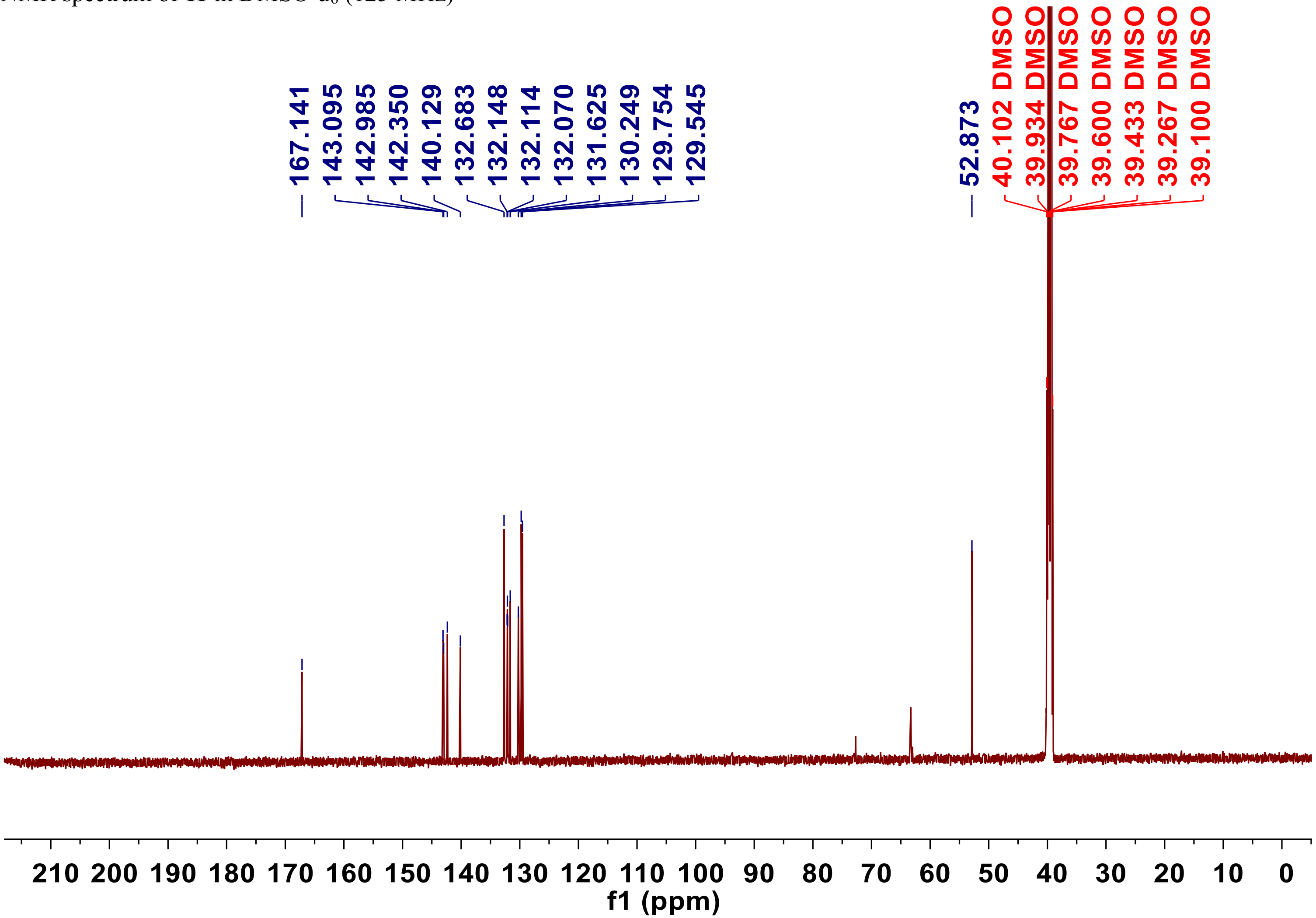

Figure S51 DEPT-90 spectrum of **11**

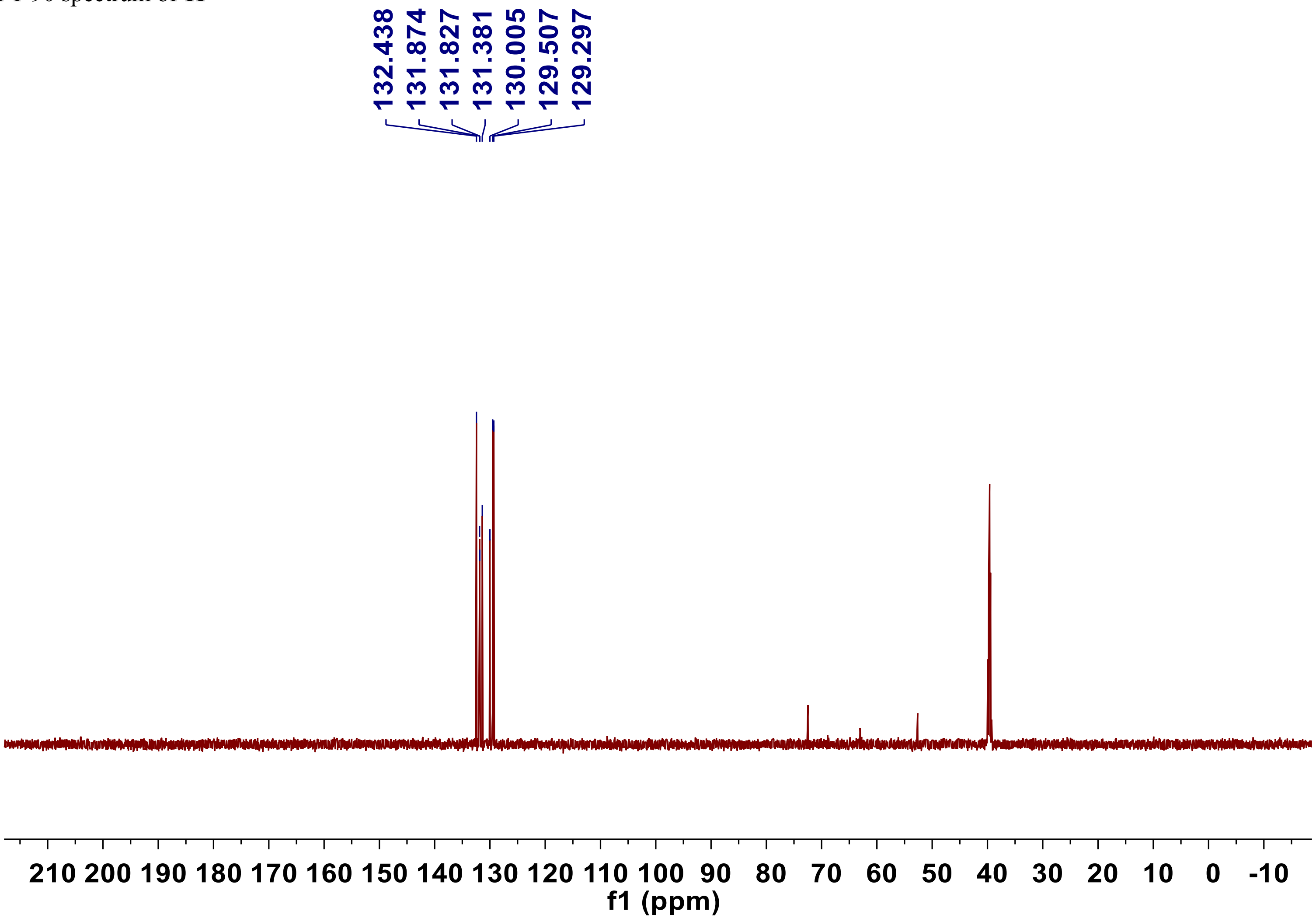

Figure S52 DEPT-135 spectrum of **11**

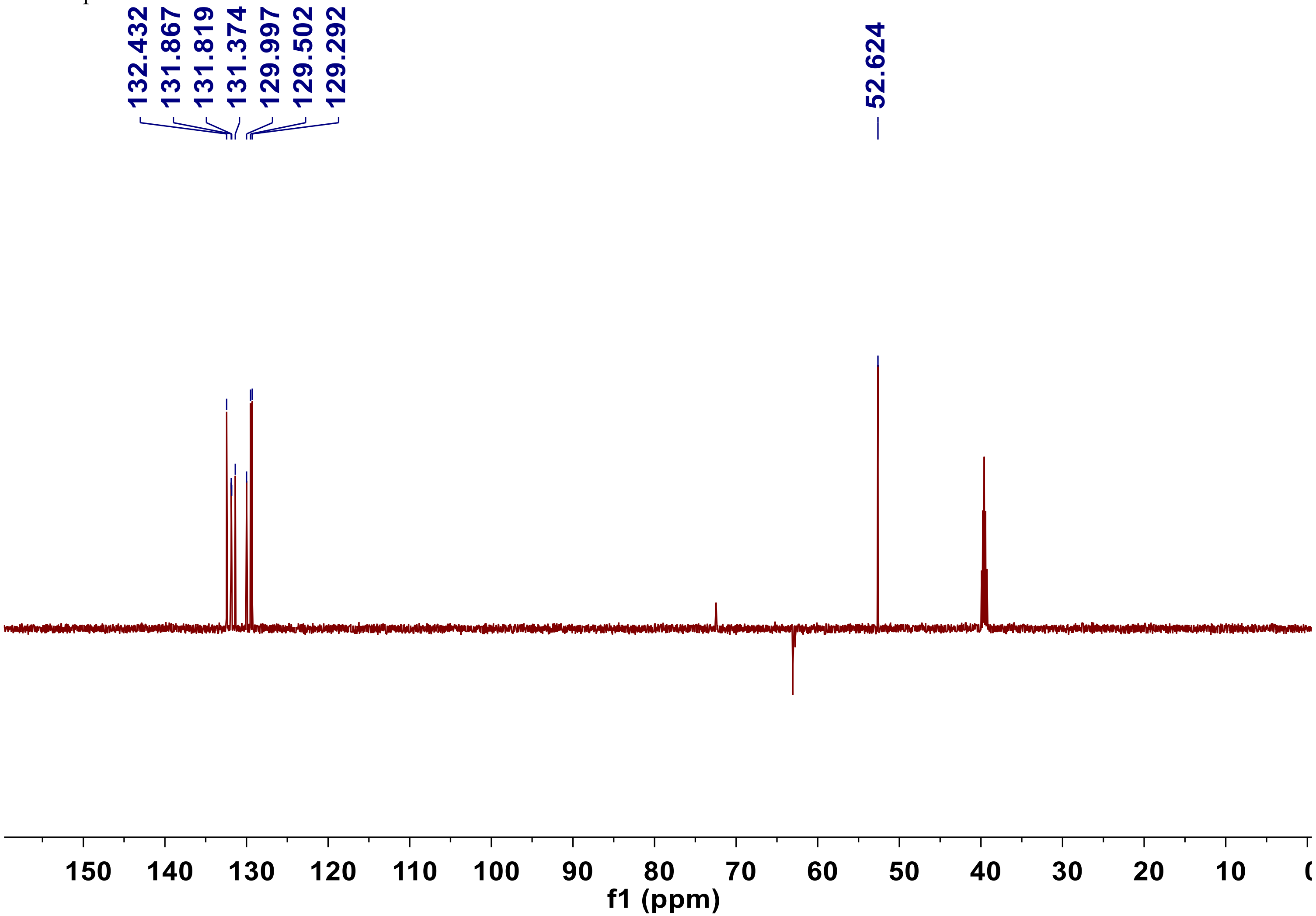

Figure S53 HSQC spectrum of **11**

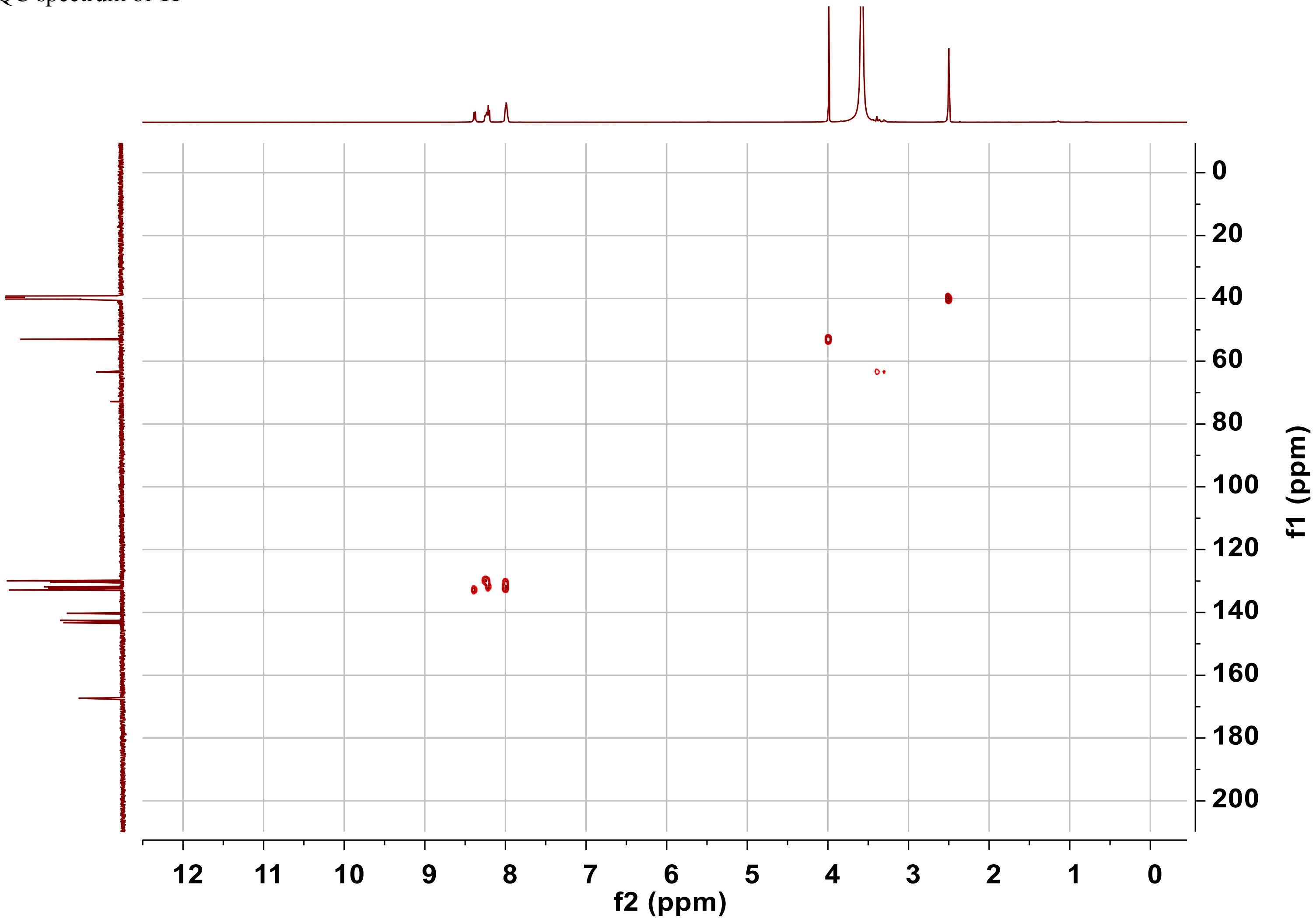

Figure S54 HMBC spectrum of **11**

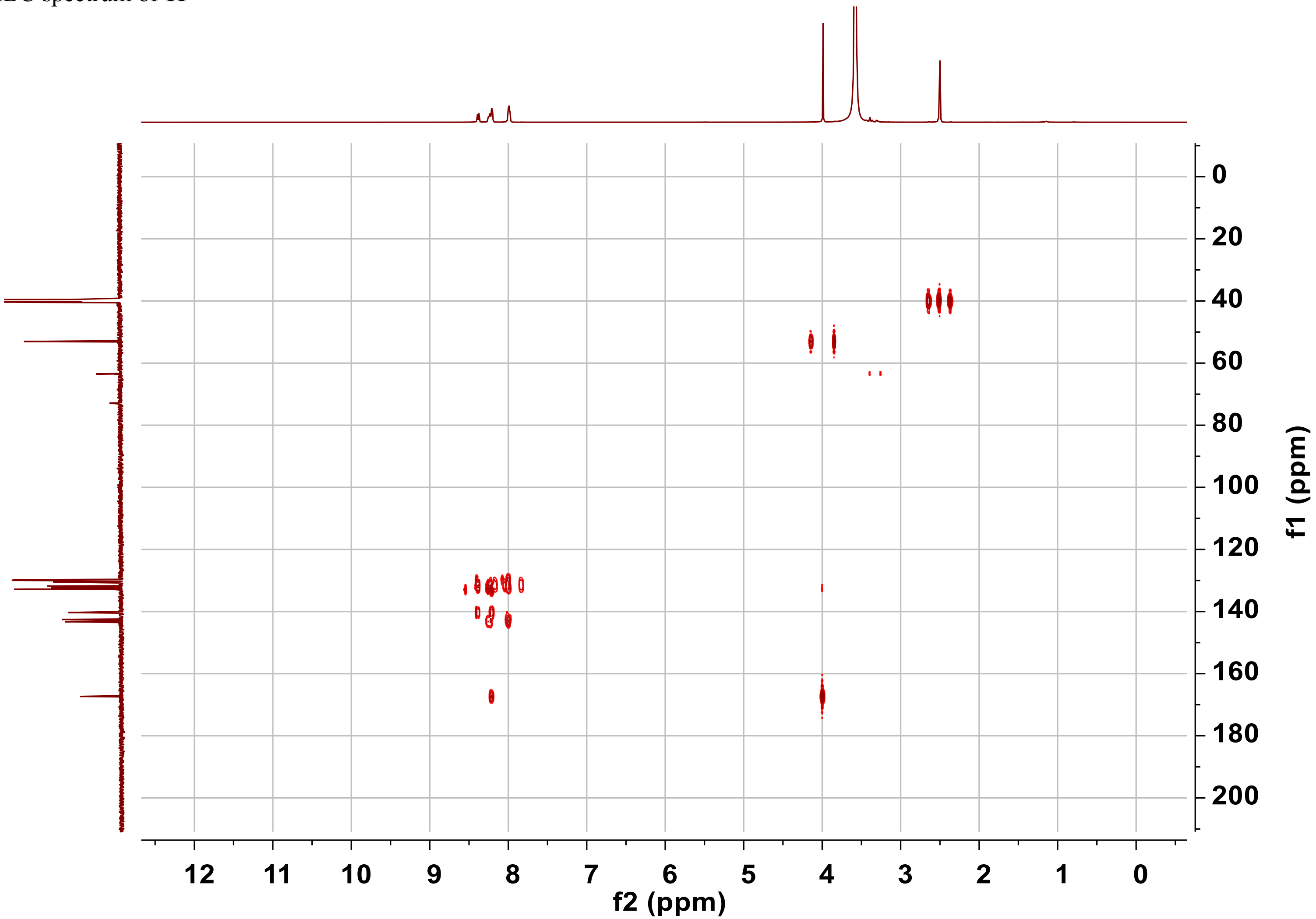

Figure S55 ^{1}H-^{1}H COSY spectrum of **11**

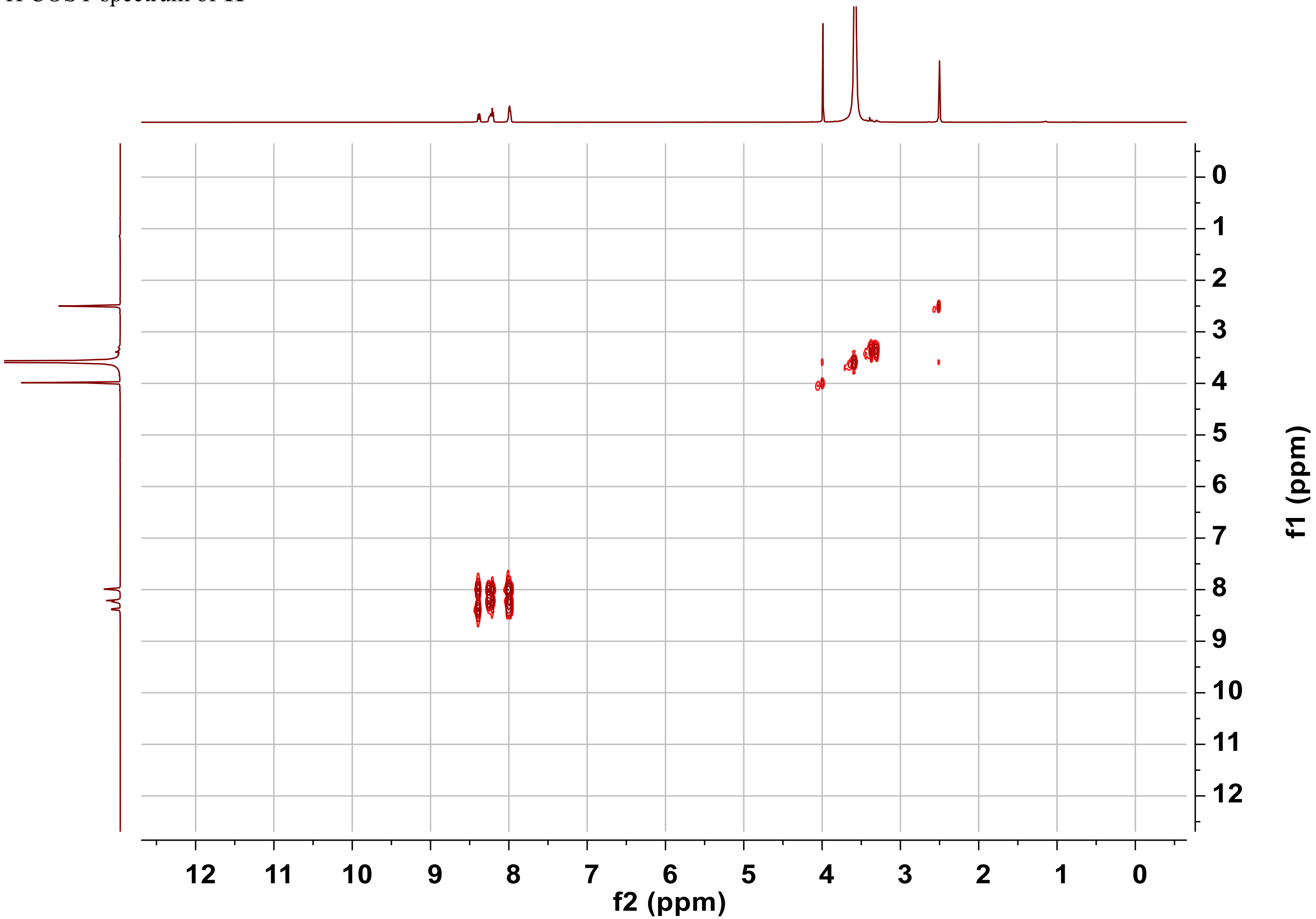

Figure S56 NOESY spectrum of **11**

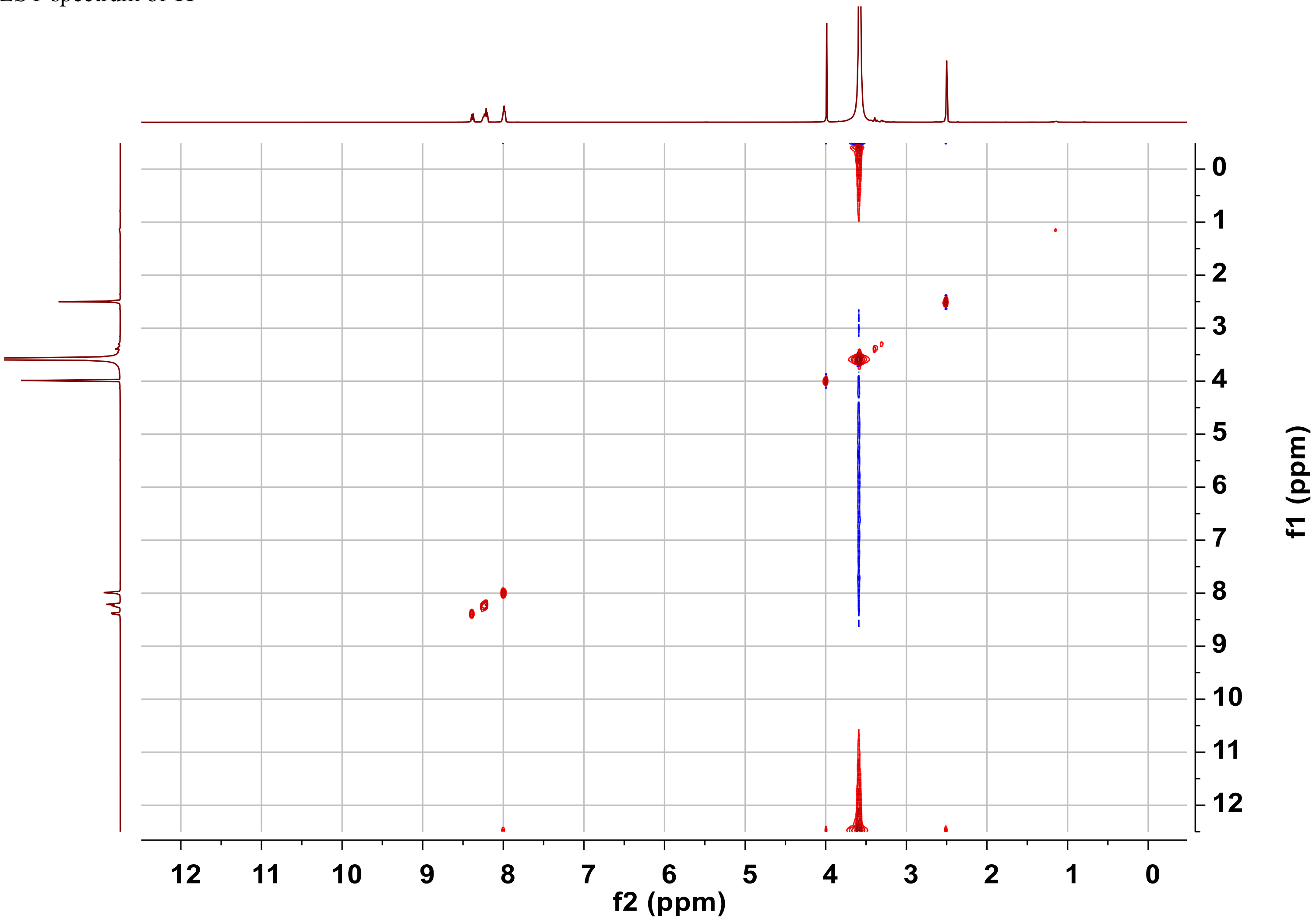

Figure S57 HRESIMS spectrum of **11**

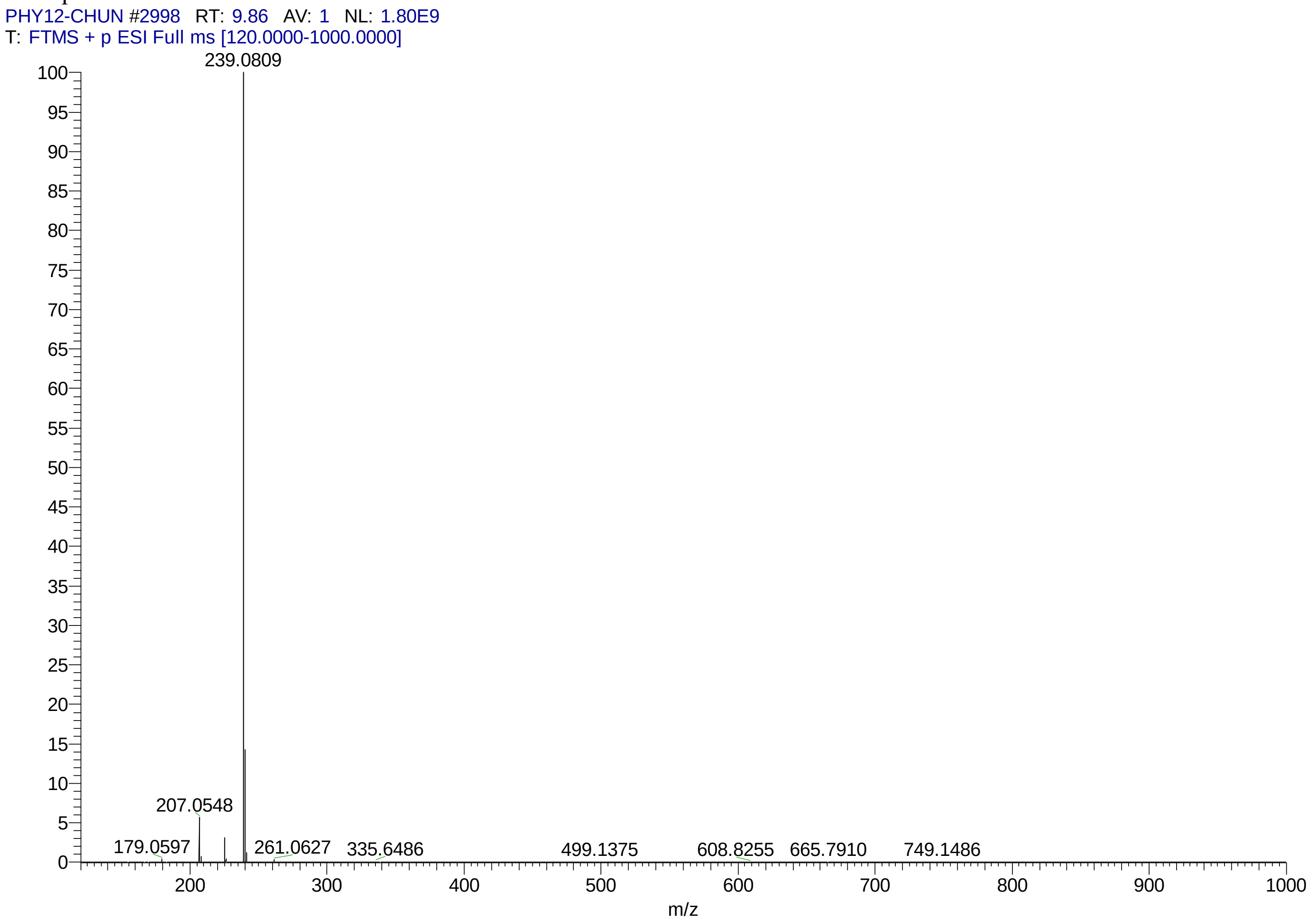

Figure S58 UV spectrum of **11**

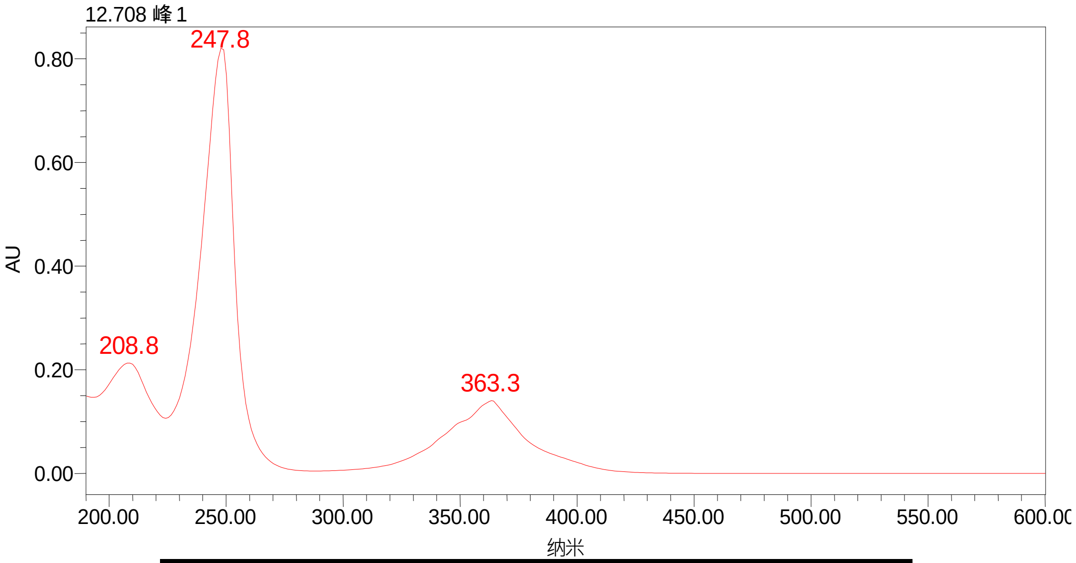

No.	Wavelength (nm)	Abs
1	208.83	0.21317
2	247.77	0.82076
3	363.32	0.14035

Figure S59 ^{1}H NMR spectrum of **12** in DMSO-d_6 (500 MHz)

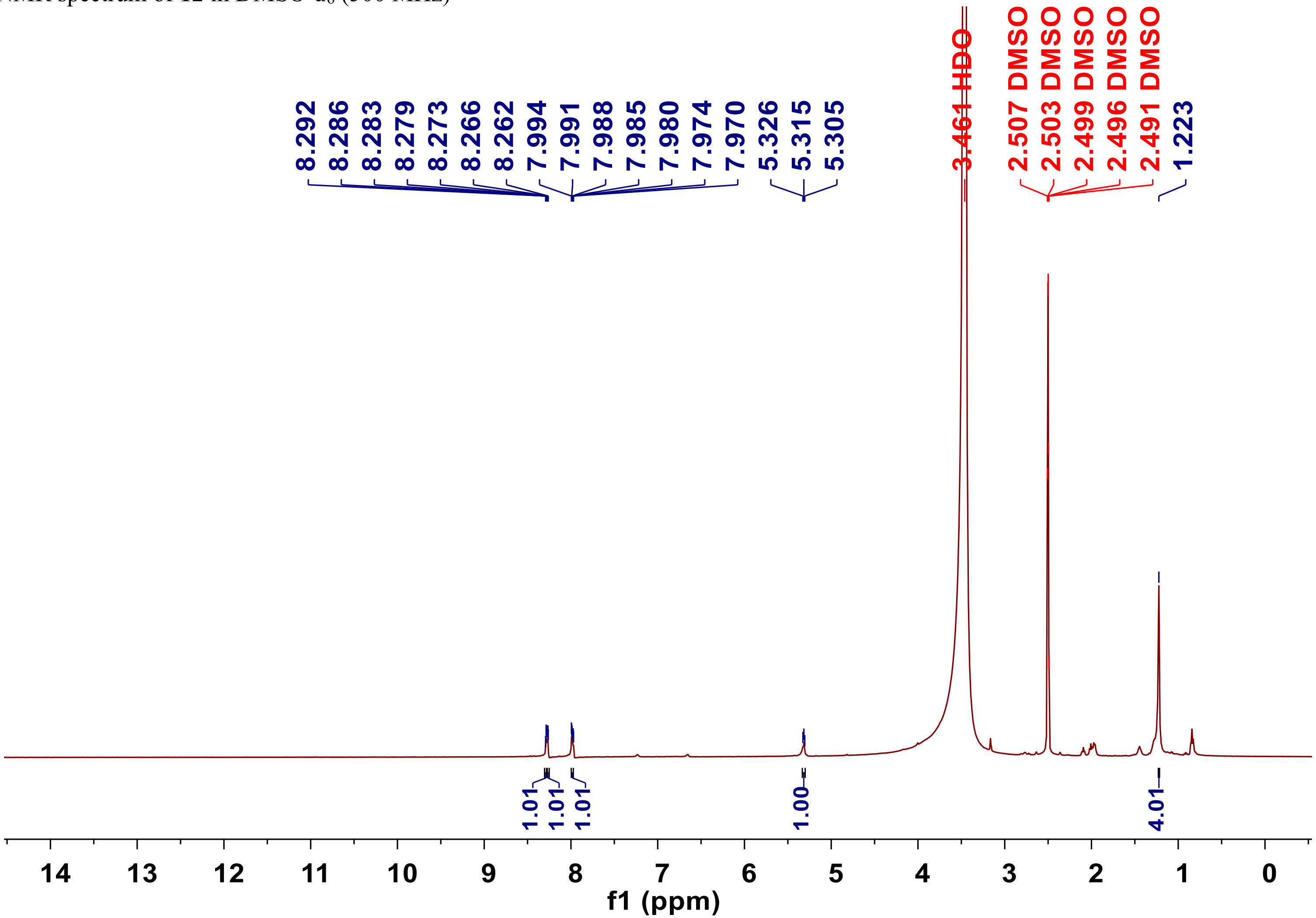

Figure S60 ^{13}C NMR spectrum of **12** in DMSO-d_6 (125 MHz)

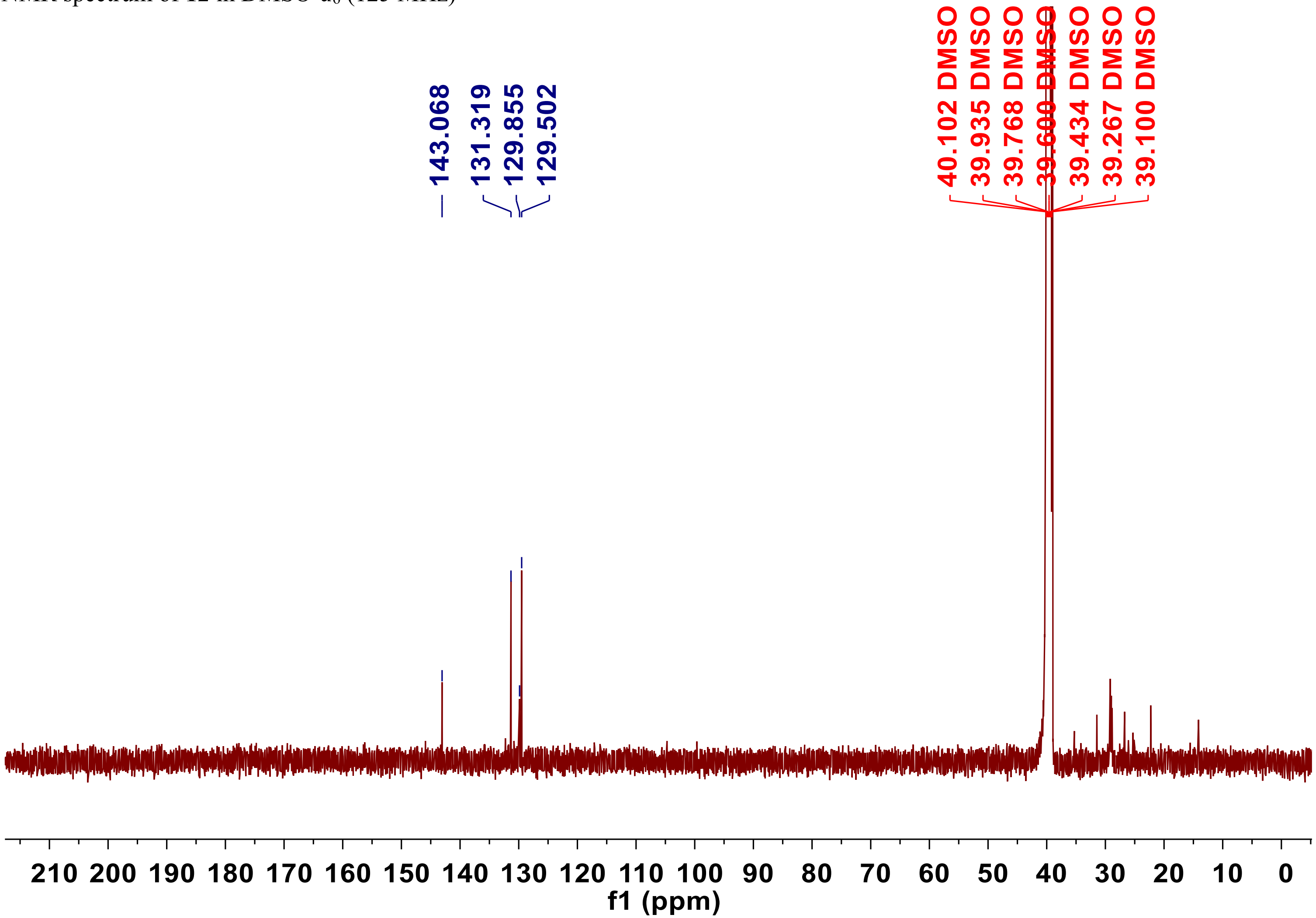

Figure S61 HRESIMS spectrum of **12**

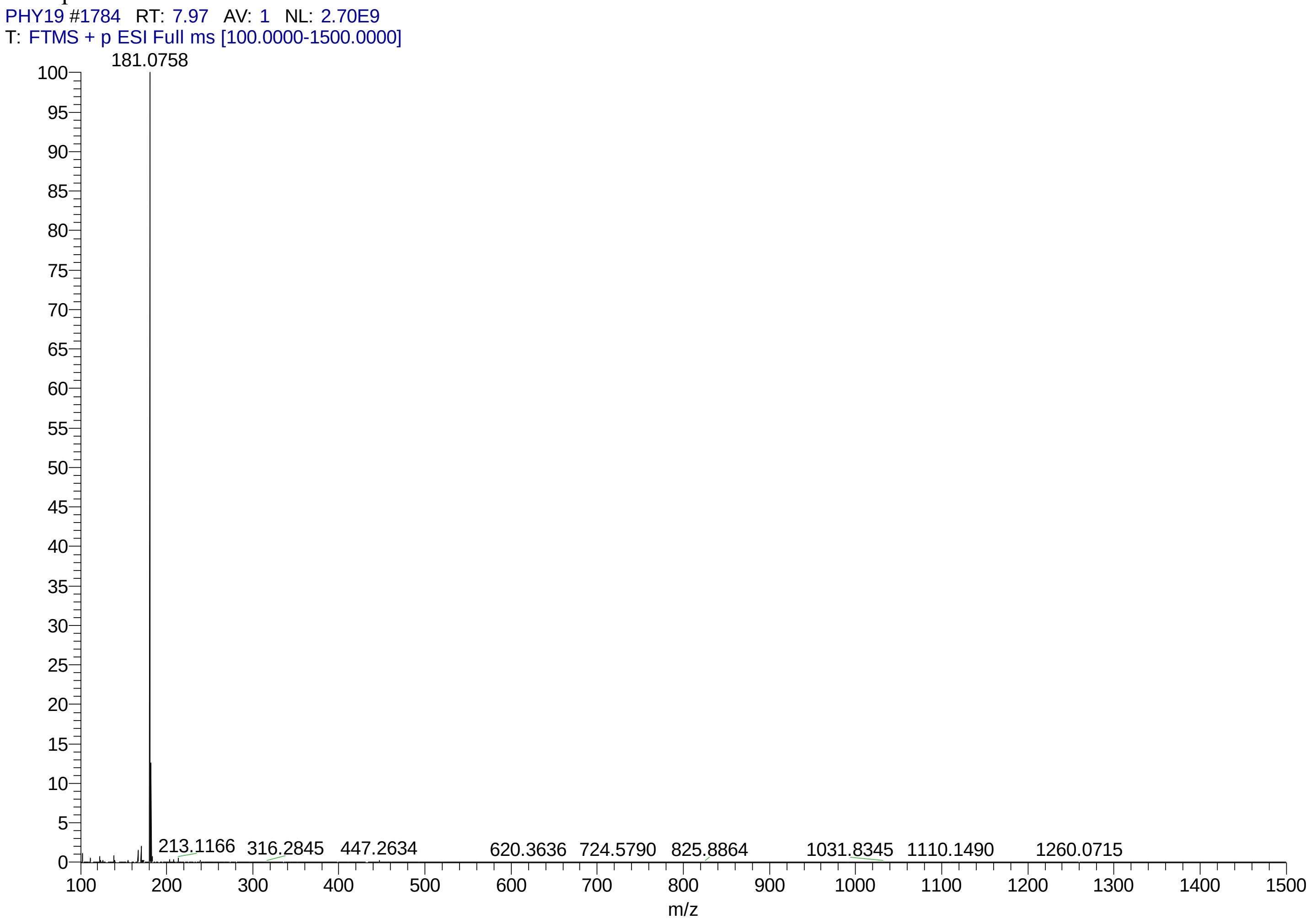

Figure S62 UV spectrum of **12**

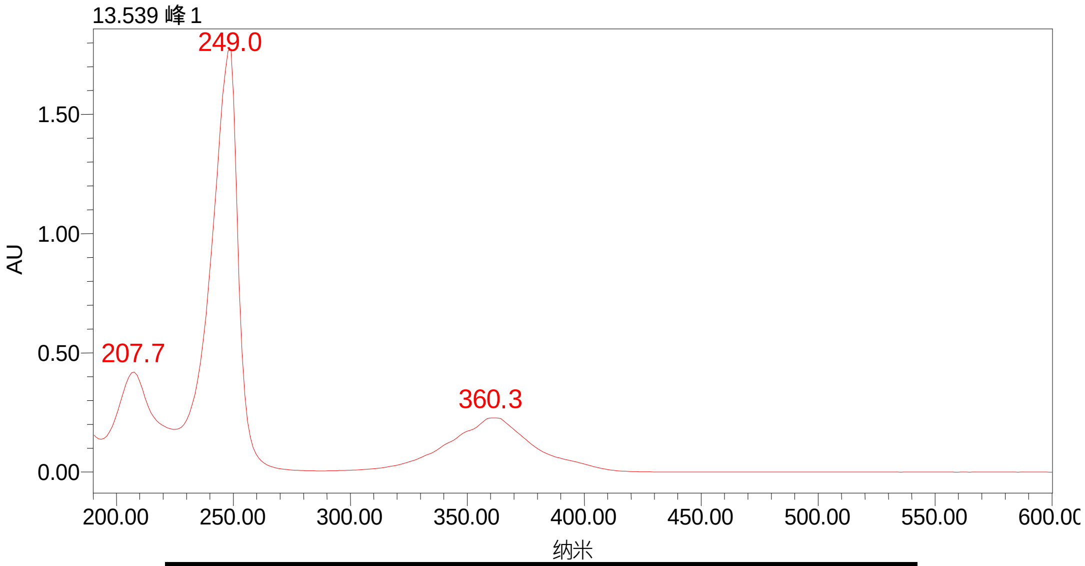

No.	Wavelength (nm)	Abs
1	207.65	0.41890
2	248.95	1.77004
3	360.32	0.22736

Figure S63 Docking poses and interactions of acarbose with α-glucosidase (PDB ID: 2QMJ)

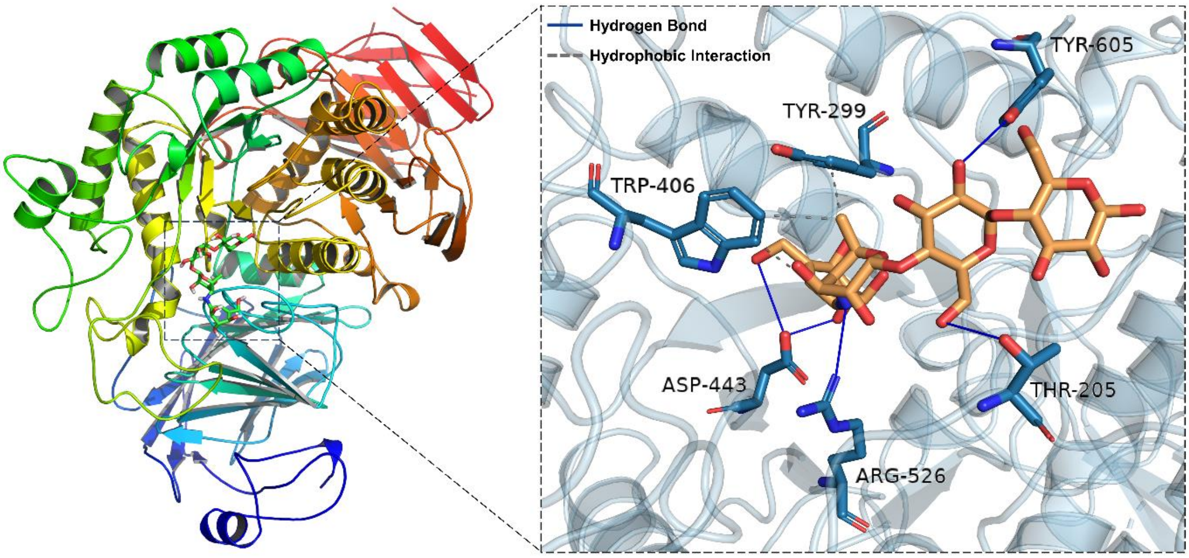

Figure S64 96-well plate assay of **5** – **12** against *Staphylococcus aureus* ATCC 29213 (A), MRSA (B), *Klebsiella pneumoniae* ATCC 13883 (C) and *Pseudomonas aeruginosa* ATCC 9027 (D) using the microbroth dilution method.

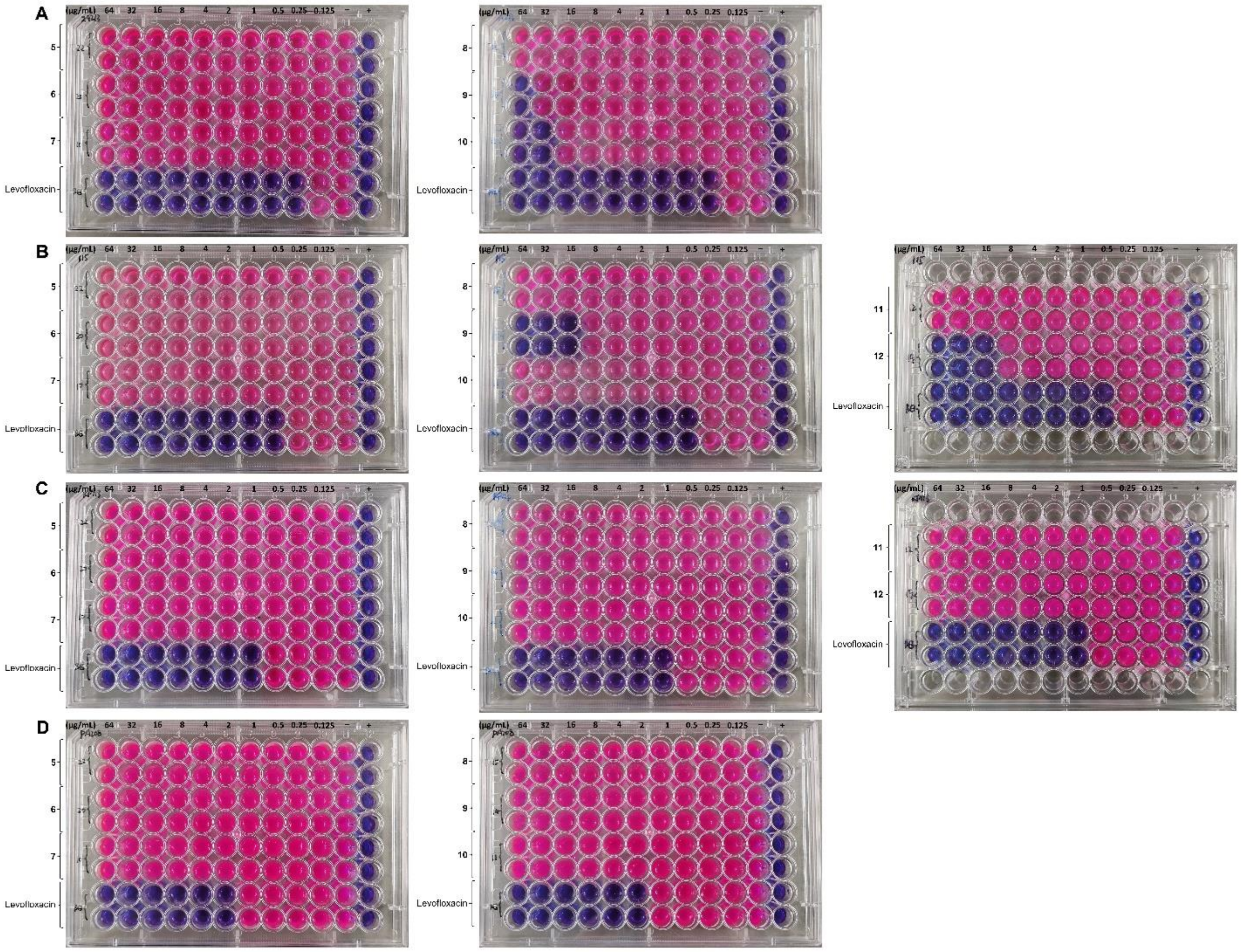

References

1. Bruhn, T.; Schaumlöffel, A.; Hemberger, Y.; Bringmann, G. SpecDis: Quantifying the Comparison of Calculated and Experimental Electronic Circular Dichroism Spectra. *Chirality* **2013**, *25*, 243–249, doi:10.1002/chir.22138.
2. Neese, F. The ORCA Program System. *WIREs Comput. Mol. Sci.* **2012**, *2*, 73–78, doi:10.1002/wcms.81.
3. Stephens, P.J.; Harada, N. ECD Cotton Effect Approximated by the Gaussian Curve and Other Methods. *Chirality* **2010**, *22*, 229–233, doi:10.1002/chir.20733.
4. Xu, X.-T.; Deng, X.-Y.; Chen, J.; Liang, Q.-M.; Zhang, K.; Li, D.-L.; Wu, P.-P.; Zheng, X.; Zhou, R.-P.; Jiang, Z.-Y.; et al. Synthesis and Biological Evaluation of Coumarin Derivatives as α-Glucosidase Inhibitors. *Eur. J. Med. Chem.* **2020**, *189*, 112013, doi:10.1016/j.ejmech.2019.112013.
5. Trott, O.; Olson, A.J. AutoDock Vina: Improving the Speed and Accuracy of Docking with a New Scoring Function, Efficient Optimization, and Multithreading. *J. Comput. Chem.* **2010**, *31*, 455–461, doi:10.1002/jcc.21334.
6. Sanner, M.F. Python: A Programming Language for Software Integration and Development. *J. Mol. Graph. Model.* **1999**, *17*, 57–61.
7. Morris, G.M.; Huey, R.; Lindstrom, W.; Sanner, M.F.; Belew, R.K.; Goodsell, D.S.; Olson, A.J. AutoDock4 and AutoDockTools4: Automated Docking with Selective Receptor Flexibility. *J. Comput. Chem.* **2009**, *30*, 2785–2791, doi:10.1002/jcc.21256.
8. Salomon-Ferrer, R.; Case, D.A.; Walker, R.C. An Overview of the Amber Biomolecular Simulation Package. *WIREs Comput. Mol. Sci.* **2013**, *3*, 198–210, doi:10.1002/wcms.1121.
9. Sagui, C.; Darden, T.A. Molecular Dynamics Simulations of Biomolecules: Long-Range Electrostatic Effects. *Annu. Rev. Biophys. Biomol. Struct.* **1999**, *28*, 155–179, doi:10.1146/annurev.biophys.28.1.155.
10. Kräutler, V.; Van Gunsteren, W.F.; Hünenberger, P.H. A Fast SHAKE Algorithm to Solve Distance Constraint Equations for Small Molecules in Molecular Dynamics Simulations. *J. Comput. Chem.* **2001**, *22*, 501–508, doi:10.1002/1096-987X(20010415)22:5<501::AID-JCC1021>3.0.CO;2-V.
11. Larini, L.; Mannella, R.; Leporini, D. Langevin Stabilization of Molecular-Dynamics Simulations of Polymers by Means of Quasisymplectic Algorithms. *J. Chem. Phys.* **2007**, *126*, doi:10.1063/1.2464095.
12. Hou, T.; Wang, J.; Li, Y.; Wang, W. Assessing the Performance of the MM/PBSA and MM/GBSA Methods. 1. The Accuracy of Binding Free Energy Calculations Based on Molecular Dynamics Simulations. *J. Chem. Inf. Model.* **2011**, *51*, 69–82, doi:10.1021/ci100275a.
13. Chen, Y.; Zheng, Y.; Fong, P.; Mao, S.; Wang, Q. The Application of the MM/GBSA Method in the Binding Pose Prediction of FGFR Inhibitors. *Phys. Chem. Chem. Phys.* **2020**, *22*, 9656–9663, doi:10.1039/D0CP00831A.
14. Genheden, S.; Ryde, U. The MM/PBSA and MM/GBSA Methods to Estimate Ligand-Binding Affinities. *Expert Opin. Drug Discov.* **2015**, *10*, 449–461, doi:10.1517/17460441.2015.1032936.
15. Rastelli, G.; Rio, A.D.; Degliesposti, G.; Sgobba, M. Fast and Accurate Predictions of Binding Free Energies Using MM-PBSA and MM-GBSA. *J. Comput. Chem.* **2010**, *31*, 797–810, doi:10.1002/jcc.21372.
16. Nguyen, H.; Roe, D.R.; Simmerling, C. Improved Generalized Born Solvent Model Parameters for Protein Simulations. *J. Chem. Theory Comput.* **2013**, *9*, 2020–2034, doi:10.1021/ct3010485.
17. Weiser, J.; Shenkin, P.S.; Still, W.C. Approximate Atomic Surfaces from Linear Combinations of Pairwise Overlaps (LCPO). *J. Comput. Chem.* **1999**, *20*, 217–230, doi:10.1002/(SICI)1096-987X(19990130)20:2<217::AID-JCC4>3.0.CO;2-A.
